# NEK2-AURKA axis drives ubiquitin-dependent IFT88 degradation to suppress ciliogenesis and promote basal-type bladder cancer

**DOI:** 10.1038/s41419-026-09203-0

**Published:** 2026-08-22

**Authors:** E Du, Wei Wang, Qiang Su, Wei Huang, Yuanjiong Qi, Weijie Tang, Zhihong Zhang, Changwen Zhang, Jingxian Li

**Affiliations:** 1 Tianjin Institute of Urology, The Second Hospital of Tianjin Medical University, Tianjin, China; 2 Department of Data Science and Analytics, BI Norwegian Business School, Oslo, Norway; 3 Present Address: Norwegian Centre for Molecular Biosciences and Medicine (NCMBM), University of Oslo, Oslo, Norway

**Keywords:** Cancer therapeutic resistance, Bladder cancer

## Abstract

Loss of primary cilia is common in cancer, but its relevance to molecular subtypes of muscle-invasive bladder cancer (MIBC) is unclear. Here, we combined transcriptomic and co-expression analyses with experiments in bladder cancer cell lines, human tumor specimens, and xenografts to investigate the basis of cilia loss in basal MIBC. Primary cilia were less frequent in basal than in luminal MIBC. Depletion of IFT88 reduced ciliogenesis and increased cell proliferation and migration. Conversely, IFT88 overexpression restored cilia, delayed the appearance of palpable xenografts, and limited xenograft growth. NEK2 was enriched in basal MIBC, and higher expression in TCGA was associated with shorter disease-free survival. In basal bladder cancer cells, NEK2 knockdown increased IFT88 abundance and cilia formation and reduced proliferation and migration; NEK2 overexpression had the opposite effects. Rescue experiments showed that these effects of NEK2 depended on IFT88. NEK2 interacted with IFT88, increased its ubiquitination, and accelerated its proteasomal turnover. AURKA knockdown reduced IFT88 ubiquitination after NEK2 overexpression and attenuated the associated cilia loss, proliferation, and migration, whereas AURKA overexpression reversed the effects of NEK2 knockdown. In a xenograft experiment, pretreating IFT88-overexpressing cells with the NEK2 inhibitor JH295 produced the greatest reduction in tumor establishment and growth among the four conditions tested. These data identify a NEK2-AURKA-IFT88 pathway that promotes ubiquitin-dependent IFT88 degradation and cilia loss in basal MIBC models, providing a preclinical basis for further testing of NEK2 targeting in cilia-deficient basal MIBC.

## Introduction

Primary cilia are microtubule-based, antenna-like organelles present on most vertebrate cells, serving as crucial signaling hubs for pathways essential to development and tissue homeostasis [1]. Loss or reduction of primary cilia (PC) has been observed in multiple cancer types, including prostate, bladder, and kidney cancers [2–5]. PC deficiency promotes tumor progression through diverse molecular mechanisms. For instance, elevated Wnt signaling has been documented in non-ciliated prostate cancer cells [6], and PC deletion driven by proteins such as STIL and AURKA enhances tumor proliferation via activation of the SHH pathway [2]. In a subset of ductal carcinomas, restoration of cilia by silencing SHCBP1 is considered a potential therapeutic strategy [7], while paradoxically, cilia loss may confer resistance to Smoothened inhibitor and extend cancer cell survival [8]. Cilia ablation can suppress BCC-like tumors induced by activated Smoothened, yet accelerate tumorigenesis driven by Gli2 activation—a dual role also observed in medulloblastoma [9, 10]. These findings underscore the complex and context-dependent functions of PC in different cancer types.

Bladder cancer remains a major global health concern, with 573,278 new cases diagnosed in 2020 and projections indicating this number will double by 2040 [11]. Early detection of muscle invasion is critical for disease management, but muscle-invasive bladder cancer (MIBC) is highly metastatic and associated with poor survival outcomes [12, 13]. Advances in molecular classification have defined six consensus subtypes of MIBC: luminal nonspecified (LumNS), luminal papillary (LumP), luminal unstable (LumU), basal/squamous (Ba/Sq), stroma-rich, and neuroendocrine-like (NE-like) [14]. This stratification has enabled personalized therapeutic approaches, with studies showing basal bladder cancer may be prioritized for neoadjuvant chemotherapy [15], while luminal subtypes expressing NECTIN4 and ERBB2 may be sensitive to Enfortumab vedotin and RC48, respectively [16, 17]. Our previous research indicates that PC loss underlies MIBC proliferation, and restoring primary cilia can abrogate tumor progression [2]. However, Iruzubieta et al. found that primary cilia presence may also be implicated in bladder cancer progression [18], suggesting that the heterogeneity of bladder cancer contributes to the paradoxical roles of PC and warrants further investigation into their functions across MIBC subtypes.

In this study, we reveal that primary cilia are significantly reduced in basal-type bladder cancer, and that promoting PC formation inhibits tumor progression. We further demonstrate that NEK2 is overexpressed in basal-type disease, suppressing IFT88 expression required for PC assembly. The NEK2-specific inhibitor JH295 selectively impairs basal cell viability, with limited effect on luminal cells. Mechanistically, we show that the NEK2/AURKA axis inhibits IFT88 expression through ubiquitination-mediated degradation. These results provide a theoretical foundation for individualized treatment of bladder cancer by restoring PC formation.

## Results

### Primary cilia are depleted in basal-type bladder cancer, facilitating tumor progression

**Fig. 1:**
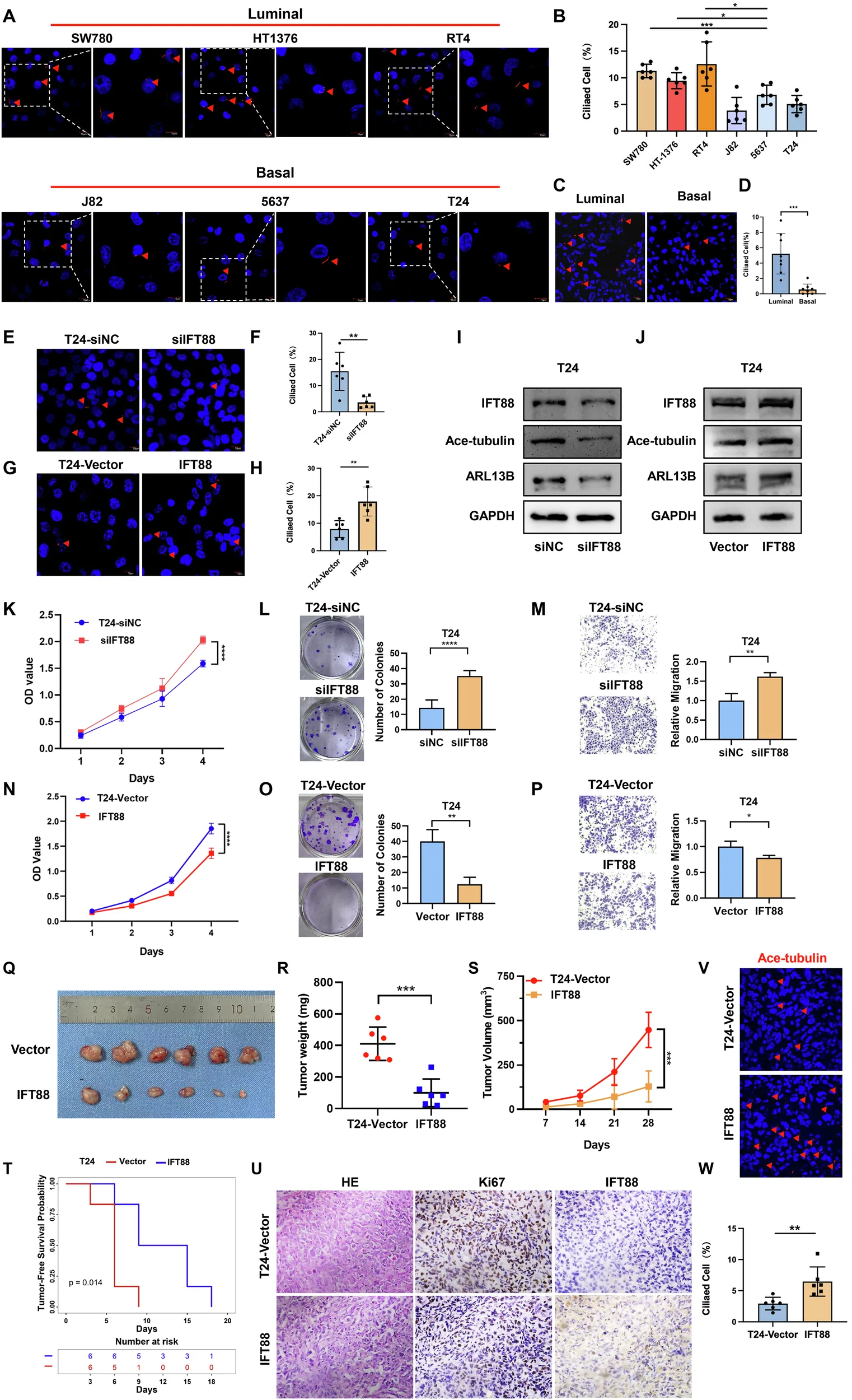
Loss of primary cilia in basal-type MIBC. **A**, **B** Luminal and basal bladder cancer cells were fixed and subjected to immunofluorescence staining with antibodies against acetylated tubulin (Ace-tubulin, red) and DAPI (blue). For each cell line, two randomly selected non-overlapping 800× microscopic fields were analyzed from each of three independent glass coverslips, resulting in six analyzed regions per condition. The percentage of ciliated cells was calculated as the number of cells with a visible acetylated tubulin-positive cilium divided by the total number of DAPI-positive cells in the same field. Dashed boxes indicate regions shown in high-magnification insets. **C**, **D** Primary cilia immunofluorescence staining was performed in a subset of classified MIBC tissues (*n* = 8 luminal tumors and *n* = 8 basal tumors). Tissues were stained with antibodies against acetylated tubulin (Ace-tubulin, red) and counterstained with DAPI (blue). For each tissue sample, two randomly selected non-overlapping microscopic fields (800× magnification) were analyzed, and the average percentage of ciliated cells was calculated. Cases were selected based on tissue availability and clear subtype assignment, rather than on the level of cilia staining. **E**–**H** Immunofluorescence staining for acetylated tubulin and DAPI in IFT88-silenced and IFT88-overexpressing T24 cells, with quantification of ciliated cell rates. For each experimental condition, two randomly selected non-overlapping 800× microscopic fields were analyzed from each of three independent glass coverslips, resulting in six analyzed regions per condition. The percentage of ciliated cells was calculated as described above. **I**, **J** Western blot analysis was performed to assess protein levels of IFT88, Ace-tubulin, and ARL13B in T24 control cells and cells with IFT88 knockdown or overexpression. **K**, **N** CCK8 assays evaluated the growth viability of T24 cells following IFT88 silencing or overexpression. **L**, **O** Colony formation assays assessed T24 cell growth in control, IFT88 knockdown, and IFT88 overexpression groups. **M**, **P** Transwell assays examined the migratory capacity of T24 cells in control, IFT88 knockdown, and IFT88 overexpression groups. **Q**–**S** Representative images of subcutaneous xenograft bladder tumors (left), statistical analysis of tumor weights (middle), and tumor volume measurements over 1–4 weeks (right). **T** Kaplan–Meier survival curves depict tumor-free survival rates between T24 vector and IFT88 overexpression groups. **U** H&E and immunohistochemical staining for Ki67 and IFT88 in T24 vector and IFT88 overexpression xenograft tissues. **V**, **W** T24 vector and IFT88 overexpression xenograft tissues were fixed and stained for Ace-tubulin (red) and DAPI (blue) for immunofluorescence analysis. For each tumor sample, two randomly selected non-overlapping microscopic fields were analyzed, and the average percentage of ciliated cells was calculated. Statistical significance: *, *P* < 0.05; **, *P* < 0.01; ***, *P* < 0.001; ****, *P* < 0.0001.

**Fig. 2:**
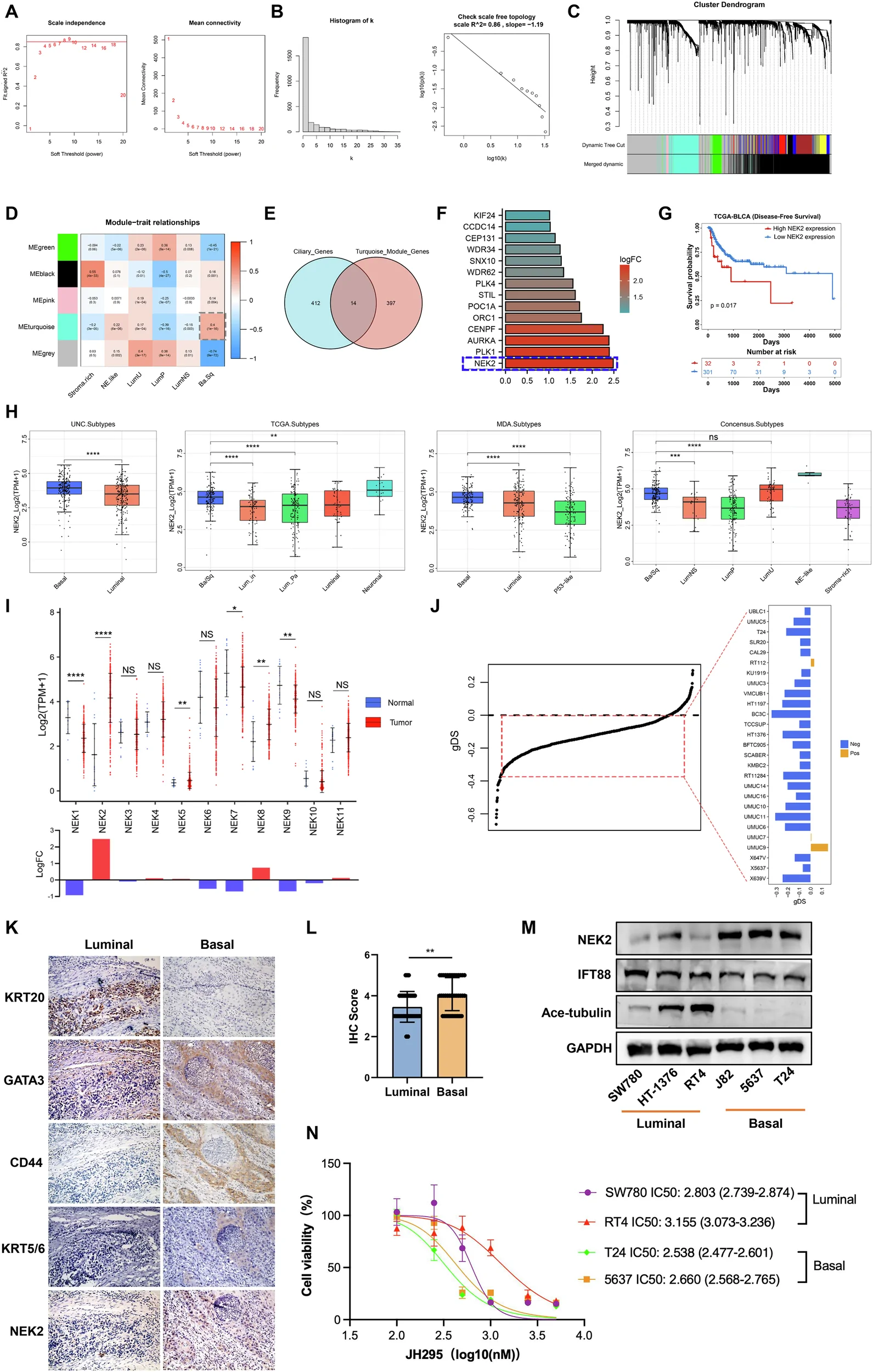
NEK2, a PC-related protein, is highly expressed in basal-type MIBC. **A** Scale-free fit index and mean connectivity analyses were performed across varying soft-thresholding powers in the TCGA-MIBC cohort to optimize network construction. **B** Evaluation of scale-free topology at soft threshold 8, where K denotes the logarithm of network connectivity and p(k) represents the logarithm of frequency distribution; p(k) is negatively correlated with K, and the correlation coefficient (*r*²) reflects the degree of scale-free topology. **C** Dendrogram illustrating the clustering of all differentially expressed genes (DEGs) based on dissimilarity (1-TOM). **D** Heatmaps depict correlations between module eigengenes and the six consensus molecular subtypes of MIBC (NE-like, LumU, LumP, LumNS, basal/squamous, stroma-rich), with p-values and correlation coefficients annotated for each module. **E** Venn diagram showing the overlap between ciliary genes and turquoise module genes, which are associated with basal/squamous MIBC; 14 overlapping PC-related genes were identified. **F** Barplot presenting the log fold change (logFC) of these 14 overlapping genes. **G** Kaplan-Meier survival curves reveal differences in disease-free survival (DFS) rates between high and low NEK2 expression groups; statistical significance was assessed by log-rank test. **H** Boxplots display NEK2 expression across MIBC subtypes as defined by UNC, TCGA, MDA, and Consensus classification systems. **I** Dotplot (top) showing mRNA expression levels of 11 NEK family members; barplot (bottom) illustrating logFC of these genes in TCGA-MIBC tumors versus adjacent normal tissue. **J** Dotplot (left) and barplot (right) indicating gene Deficiency Score (gDS) for NEK2 across 27 bladder cancer cell lines; blue bars denote gDS < 0 and orange bars denote gDS > 0. **K**, **L** Luminal (KRT20 and GATA3) and basal (CD44 and KRT5/6) MIBC subtypes were identified by their respective markers, and NEK2 expression was evaluated by immunohistochemistry; IHC scores were calculated and statistical significance determined by *t*-test. **M** Western blot analysis comparing NEK2, IFT88, and Ace-tubulin expression in luminal and basal cells. **N** The percentage of surviving cells following exposure to increasing concentrations of JH295 in two luminal (RT4, SW780) and two basal (5637, T24) cell lines. Statistical significance: NS, not significant; *, *P* < 0.05; **, *P* < 0.01; ***, *P* < 0.001; ****, *P* < 0.0001.

To investigate the distribution of primary cilia (PC) across muscle-invasive bladder cancer (MIBC) subtypes, we curated eight genes linked to PC degradation (AURKA, CENPF, KIF2A, KIF24, PLK1, PLK4, STIL, WDR62) and eight genes associated with PC formation (C21orf2, CATIP, CCDC151, DCDC2, IFT27, IFT88, NME5, TTC25) from published literature [3]. Expression profiling revealed that PC degradation genes are upregulated in basal/squamous MIBC relative to the three luminal subtypes, whereas PC formation genes are more highly expressed in luminal MIBC (Fig. S1A, B). This pattern suggested preferential PC loss in basal/squamous MIBC. To validate this hypothesis, we examined three luminal (SW780, HT-1376, RT4) and three basal (J82, 5637, T24) bladder cancer cell lines. Immunofluorescence analysis demonstrated a marked reduction in ciliated cells among basal cells compared to luminal counterparts (Fig. 1A, B). Extending this observation to patient samples, we analyzed 100 MIBC tissues and classified them using luminal markers (KRT20 and GATA3) and basal markers (CD44 and KRT5/6) (Fig. 2K). Among these cases, 32 tumors were identified as luminal and 39 as basal MIBC. Because serial FFPE sections were limited after subtype classification, primary cilia immunofluorescence was subsequently performed in a subset of cases with sufficient remaining material and unequivocal subtype-marker staining, including 8 luminal and 8 basal tumors. These cases were selected based on tissue availability and clear subtype assignment, rather than on the level of cilia staining. This analysis showed that primary cilia were significantly downregulated in basal MIBC compared with luminal MIBC (Fig. 1C, D). Collectively, these data indicate that primary cilia are deficient in basal-type bladder cancer.

Building on prior evidence that PC deficiency accelerates tumor progression, we engineered bladder cancer cell models with either suppressed or enhanced PC formation by silencing or overexpressing IFT88 (Figs. 1E–H and S1C–F). IFT88 knockdown reduced ace-tubulin and ARL13B expression, impairing PC assembly (Figs. 1I and S1G), while IFT88 overexpression elevated these markers and promoted cilia formation (Figs. 1J and S1H). Functionally, IFT88 silencing augmented basal cell proliferation, growth, and migration (Figs. 1K–M and S1I–K), whereas IFT88 overexpression suppressed these malignant phenotypes (Figs. 1N–P and S1L–N). In vivo, IFT88 overexpression attenuated tumor growth and prolonged tumor-free survival in mice (Fig. 1Q–T). Tumors derived from IFT88-overexpressing cells exhibited reduced Ki67 expression and a higher proportion of ciliated cells compared to controls (Fig. 1U–W). Together, these findings establish that loss of primary cilia is a hallmark of basal-type bladder cancer and acts as a driver of tumor progression.

### NEK2 is upregulated in basal-type bladder cancer

To elucidate the mechanisms underlying primary cilia (PC) degradation in basal-type muscle-invasive bladder cancer (MIBC), we performed differential gene expression and weighted gene coexpression network analysis (WGCNA). Differentially expressed genes (DEGs) were subjected to WGCNA, and a scale-free co-expression network was constructed using a soft-thresholding power of *β* = 8 in the TCGA-MIBC dataset (Fig. 2A). Scale-free topology was confirmed when the correlation between network connectivity (k) and its frequency distribution (p(k)) exceeded 0.85 (Fig. 2B). Highly similar modules were merged using a cut height of < 0.25 and a minimum module size of 30, based on the dynamic hybrid tree cut method (Fig. 2C). Among the identified modules, the turquoise module exhibited a strong positive correlation with basal-type MIBC (***r*** = 0.4, *p* = 1e-16; Fig. 2D). Intersecting ciliary genes with turquoise module members yielded 14 PC-related genes, with NEK2 emerging as significantly upregulated in MIBC (Fig. 2E, F). High NEK2 expression was associated with poor disease-free survival (Fig. 2G). Across various classification systems, NEK2 consistently displayed higher expression in basal-type compared to luminal-type bladder cancer (Fig. 2H). Further analysis of NEK family members revealed that NEK2 was the only member markedly overexpressed in MIBC relative to normal tissue (LogFC > 2, *P* < 0.0001; Fig. 2I). Gene Deficiency Score (gDS) analysis indicated that NEK2 is essential for cell viability in most bladder cancer cell lines (gDS < 0; Fig. 2J). Comparisons between luminal and basal MIBC tissues and cell lines confirmed elevated NEK2 expression in basal-type disease, whereas IFT88 and acetylated tubulin were downregulated in basal MIBC cells (Fig. 2K–M). In addition, NEK2 expression was significantly associated with basal and luminal subtype status, with high NEK2 expression observed predominantly in basal bladder cancer (Table 1). Notably, basal cells exhibited greater sensitivity to the NEK2 inhibitor JH295 than luminal cells, suggesting a potential therapeutic advantage for basal-type MIBC when treated with JH295 (Fig. 2N). However, INH1, which targets Hec1 and disrupts its interaction with NEK2, did not show a significant differential inhibitory effect between luminal and basal cells (Fig. S3A, B).

**Table 1:** Correlation between NEK2 expression and clinical information.

Parameters	Number of cases	NEK2 expression		*P* Value
		Low	High	
Age(Years)				1.0000
<60	9	4	5	
≥60	62	26	36	
Gender				0.0949
Male	56	27	29	
Female	15	3	12	
Grade				0.8834
Well	3	1	2	
Moderate	6	3	3	
Poor	62	26	36	
TNM stage				0.9684
T2	39	17	22	
T3	27	11	16	
T4	5	2	3	
Lymph node metastasis				1.0000
Negative	67	28	39	
Positive	4	2	2	
Molecular subtypes				0.0162
Luminal	32	19	13	
Basal	39	11	28	

### NEK2 promotes tumor progression and suppresses primary cilia formation

**Fig. 3:**
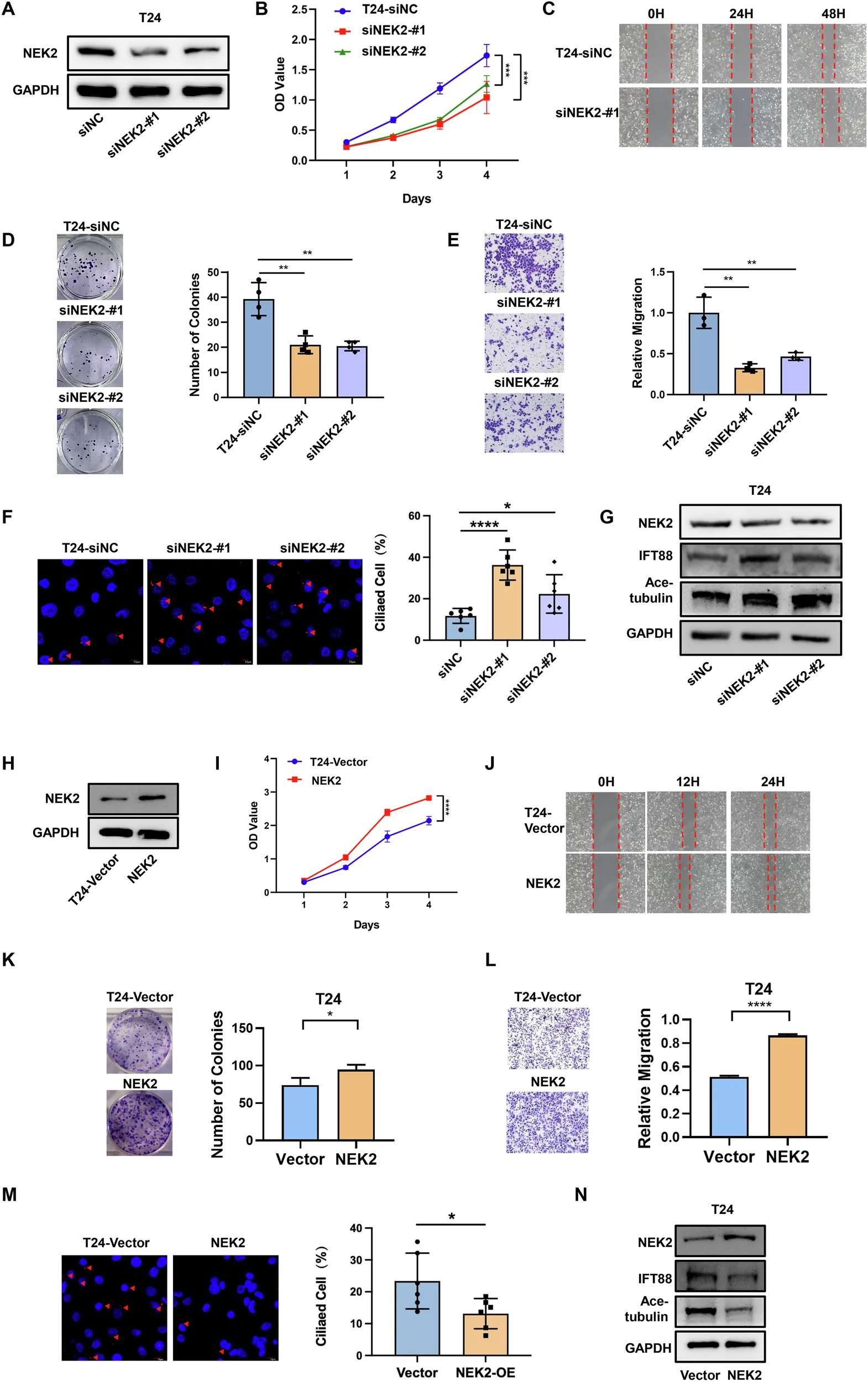
NEK2 promotes basal cell progression and inhibits primary cilia formation. **A** Western blot analysis confirms effective NEK2 knockdown in T24 cells. **B** CCK8 assays assess proliferation in T24-siNC, siNEK2-#1, and siNEK2-#2 groups over days 1–4. **C** Wound healing assays evaluate migration capacity in T24-siNC and T24-siNEK2-#1 cells at 0, 24, and 48 h. **D** Colony formation assays quantify tumor growth in T24-siNC, siNEK2-#1, and siNEK2-#2 groups, with colony numbers compared by *t*-test. **E** Transwell assays measure migration in T24-siNC, siNEK2-#1, and siNEK2-#2 groups; relative migration (number of cells per group/average number in control) was statistically analyzed by *t*-test. **F** Immunofluorescence staining for acetylated tubulin identifies the percentage of ciliated cells in T24-siNC, siNEK2-#1, and siNEK2-#2 groups. For each experimental condition, two randomly selected non-overlapping 800× microscopic fields were analyzed from each of three independent glass coverslips, resulting in six analyzed regions per condition. The percentage of ciliated cells was calculated as the number of cells with a visible acetylated tubulin-positive cilium divided by the total number of DAPI-positive cells in the same field. **G** Western blot detection of NEK2, IFT88, and acetylated tubulin protein levels in T24-siNC, siNEK2-#1, and siNEK2-#2 groups. **H** Western blot validates NEK2 overexpression in T24 cells. **I** CCK8 assays assess proliferation in T24-vector and NEK2-overexpression groups over days 1–4. **J** Wound healing assays evaluate migration in T24-vector and NEK2-overexpression groups at 0, 12, and 24 h. **K** Colony formation assays quantify tumor growth in T24-vector and NEK2-overexpression groups, with colony numbers compared by *t*-test. **L** Transwell assays measure migration in T24-vector and NEK2-overexpression groups; relative migration was statistically analyzed by *t*-test. **M** Immunofluorescence staining for acetylated tubulin identifies the percentage of ciliated cells in T24-vector and NEK2-overexpression groups. For each experimental condition, two randomly selected non-overlapping 800× microscopic fields were analyzed from each of three independent glass coverslips, resulting in six analyzed regions per condition. The percentage of ciliated cells was calculated as described above. **N** Western blot detection of NEK2, IFT88, and acetylated tubulin protein levels in T24-vector and NEK2-overexpression groups. Statistical significance: NS not significant; *, *P* < 0.05; **, *P* < 0.01; ***, *P* < 0.001; ****, *P* < 0.0001.

Previous studies have shown that serum starvation upregulates Ace-tubulin expression, indicative of enhanced primary cilia (PC) formation, whereas serum re-stimulation suppresses Ace-tubulin levels [2]. To clarify the role of NEK2 in basal bladder cancer, we first examined NEK2 expression under serum starvation and re-stimulation conditions. NEK2 levels progressively declined over 0, 24, and 48 h of serum starvation, but were restored following 24 h of serum re-stimulation (Fig. S2A), suggesting an inverse relationship between NEK2 and Ace-tubulin during cell cycle progression. To interrogate the functional consequences of NEK2 modulation, we silenced NEK2 using two distinct siRNAs in T24 and 5637 bladder cancer cell lines (Figs. 3A and S2B). NEK2 knockdown resulted in a marked reduction in cellular proliferation, growth, and migration (Figs. 3B–E and S2C–F). Importantly, NEK2 depletion promoted PC formation, accompanied by increased expression of IFT88 and Ace-tubulin (Figs. 3F, G and S2G, H). Conversely, NEK2 overexpression (Figs. 3H and S2I) enhanced cell proliferation, growth, and migration (Figs. 3I–L and S2J–M), while simultaneously suppressing PC formation and downregulating IFT88 and Ace-tubulin expression (Figs. 3M, N and S2N, O). Collectively, these findings establish NEK2 as a key driver of tumor progression in basal bladder cancer, acting through the inhibition of primary cilia assembly.

### NEK2 promotes tumor progression by regulating primary cilia formation

**Fig. 4:**
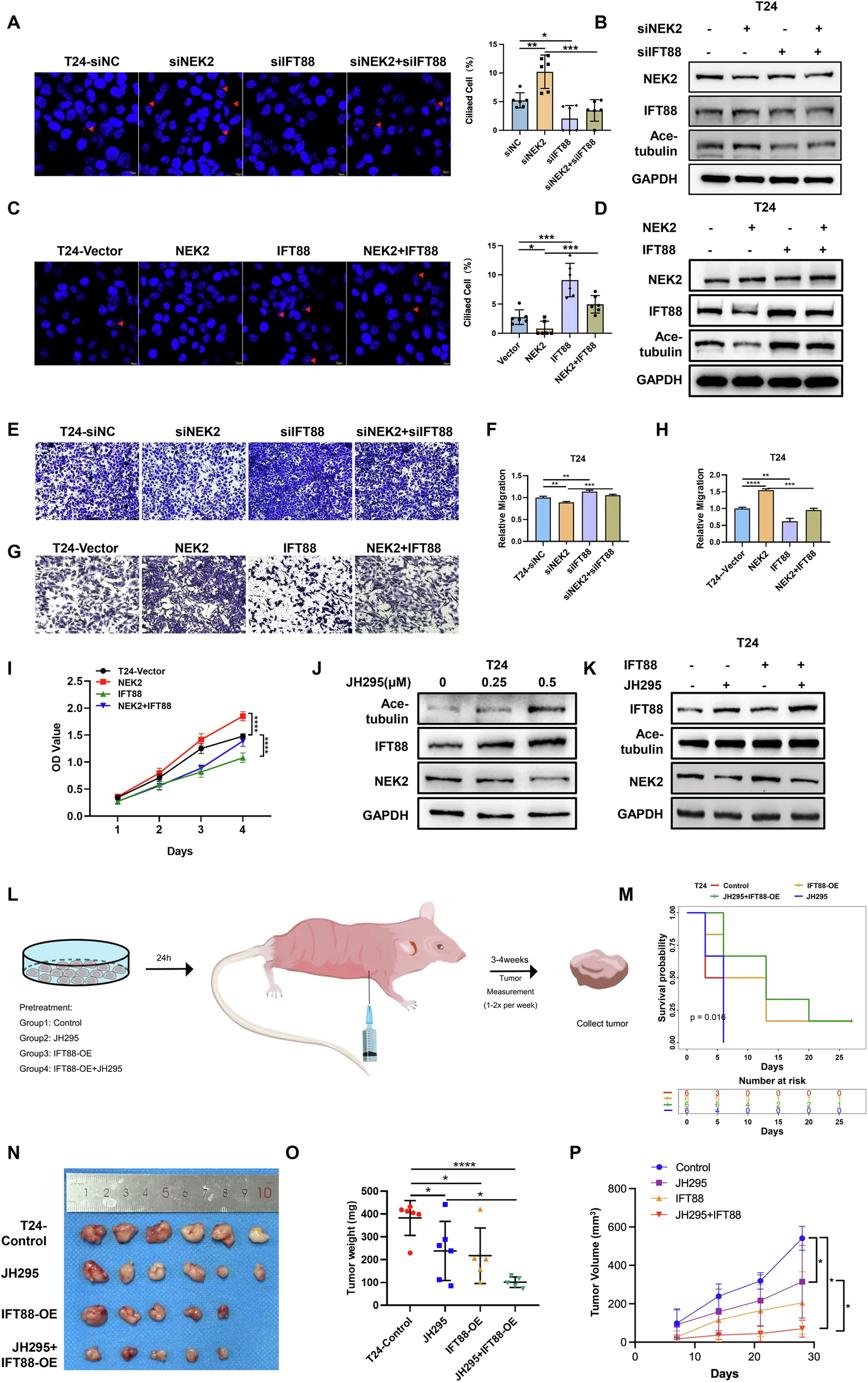
NEK2 inhibits primary cilia formation by downregulating IFT88 expression. Immunofluorescence assessed the percentage of ciliated cells in T24-siNC, siNEK2, siIFT88, and siNEK2+siIFT88 groups (**A**), and in T24-vector, NEK2-overexpression, IFT88-overexpression, and NEK2 + IFT88-overexpression groups (**C**). For each experimental condition, two randomly selected non-overlapping 800× microscopic fields were analyzed from each of three independent glass coverslips, resulting in six analyzed regions per condition. The percentage of ciliated cells was calculated as the number of cells with a visible acetylated tubulin-positive cilium divided by the total number of DAPI-positive cells in the same field. Western blot assays assessed NEK2, IFT88, and acetylated tubulin protein levels in T24-siNC, siNEK2, siIFT88, and siNEK2+siIFT88 groups (**B**), and in T24-vector, NEK2-overexpression, IFT88-overexpression, and NEK2 + IFT88-overexpression groups (**D**). **E**, **F** Transwell assays evaluated migration capacity in T24-siNC, siNEK2, siIFT88, and siNEK2+siIFT88 cells. **G**, **H** Transwell assays assessed migration in T24-vector, NEK2-overexpression, IFT88-overexpression, and NEK2 + IFT88-overexpression cells. **I** CCK8 assays measured malignant proliferation in T24-vector, NEK2-overexpression, IFT88-overexpression, and NEK2 + IFT88-overexpression groups. **J** Western blot analysis of acetylated tubulin, IFT88, and NEK2 expression following treatment with increasing concentrations of JH295 (0, 0.25, and 0.5 μM). **K** Western blot detection of IFT88, acetylated tubulin, and NEK2 in control and IFT88-overexpression cells with or without JH295 treatment. **L** Experimental flowchart for in vivo studies: T24-vector and IFT88-overexpression cells, with or without JH295 pretreatment, were injected subcutaneously into nude mice, and tumor growth and size were monitored periodically. Tumors were harvested 3–4 weeks post-injection. **M** Kaplan–Meier survival curves illustrate tumor-free survival rates among T24-control, JH295, IFT88-overexpression, and JH295 + IFT88-overexpression groups; log-rank tests determined statistical significance. **N** Representative images of tumors from T24-control, JH295, IFT88-OE, and JH295 + IFT88-OE groups. **O** Tumor weights (mg) for each experimental group, with *t*-tests used for group comparisons. **P** Tumor volumes recorded over weeks 1–4. Statistical significance: *, *P* < 0.05; **, *P* < 0.01; ***, *P* < 0.001; ****, *P* < 0.0001.

To dissect the mechanistic role of NEK2 in basal bladder cancer, we silenced IFT88 in NEK2 knockdown T24 and 5637 cells. Remarkably, the increase in PC formation resulting from NEK2 depletion was reversed upon IFT88 deletion (Figs. 4A and S4A). Similarly, the upregulation of IFT88 and Ace-tubulin induced by NEK2 knockdown was abrogated by concurrent IFT88 silencing (Figs. 4B and S4B). Conversely, NEK2 overexpression-mediated suppression of PC formation could be rescued by IFT88 overexpression (Figs. 4C and S4C), with restoration of IFT88 and Ace-tubulin levels (Figs. 4D and S4D). Functionally, the inhibition of cell migration and proliferation observed with NEK2 silencing was reversed by IFT88 knockdown, while NEK2-driven increases in cell migration and proliferation were counteracted by IFT88 overexpression (Figs. 4E–I and S4E–G). Pharmacological inhibition of NEK2 with JH295 resulted in decreased NEK2 expression and concomitant upregulation of Ace-tubulin and IFT88 (Figs. 4J and S4H). These effects were further potentiated in IFT88-overexpressing basal cells treated with JH295 (Figs. 4K and S4I). To evaluate these findings in vivo, T24 cells and IFT88-overexpressing T24 cells, with or without JH295 treatment, were injected subcutaneously into mice (Fig. 4L). Mice receiving IFT88-overexpressing cells treated with JH295 exhibited significantly prolonged tumor-free survival (Fig. 4M), and both tumor weight and volume were markedly reduced in the JH295 + IFT88-OE group compared to all other cohorts (Fig. 4N–P), indicating a synergistic effect between IFT88 overexpression and NEK2 inhibition in suppressing tumor growth.

### NEK2 promotes the ubiquitination and degradation of IFT88

**Fig. 5:**
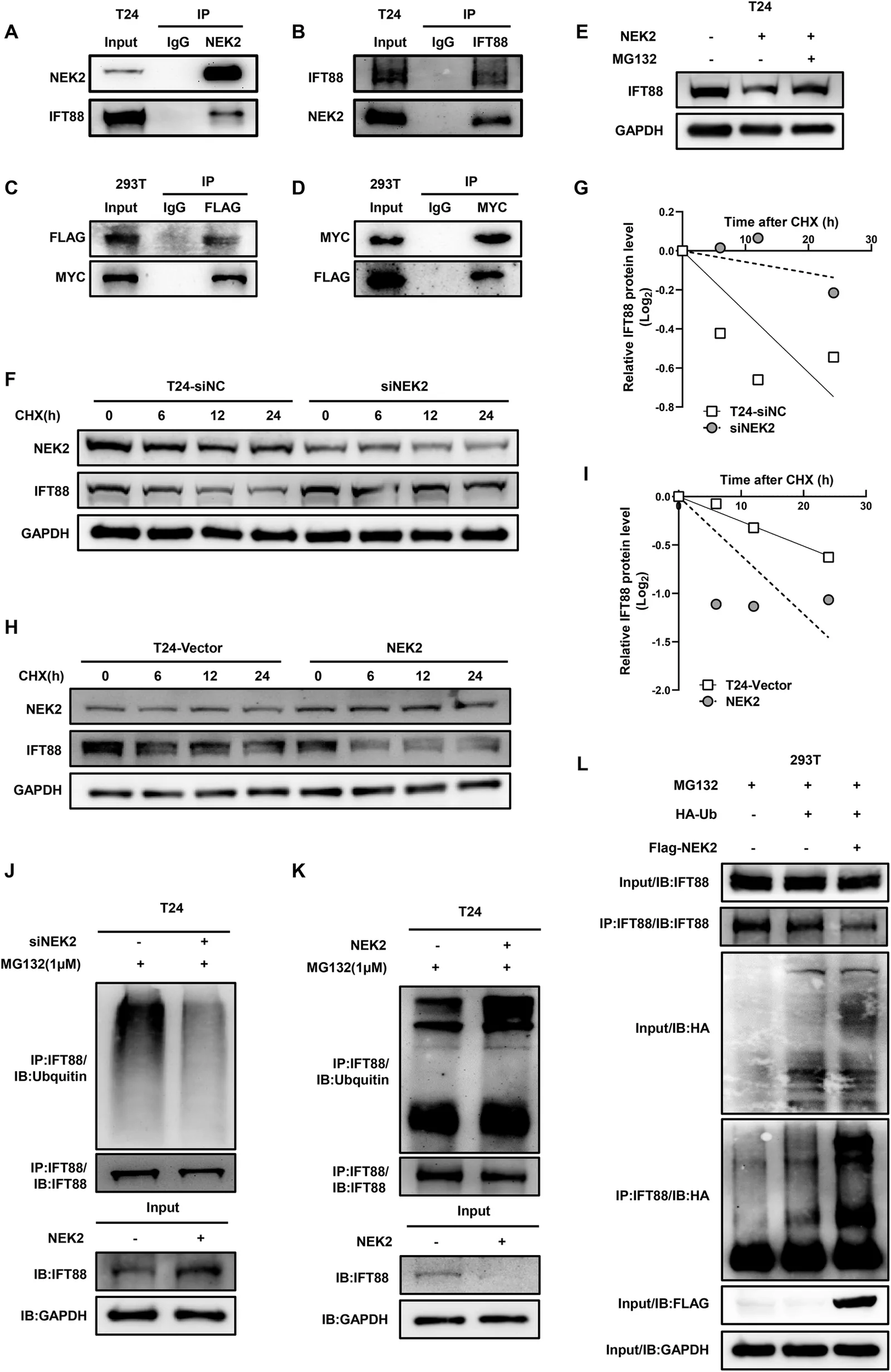
NEK2 promotes IFT88 degradation via ubiquitination. **A**, **B** Co-immunoprecipitation (Co-IP) and western blot analyses demonstrate a physical interaction between NEK2 and IFT88 in T24 cells. **C**, **D** Co-IP and western blot assays confirm the interaction between Flag-tagged NEK2 and Myc-tagged IFT88 in 293 T cells. **E** Western blot analysis of IFT88 expression in NEK2-overexpressing cells with or without MG132 treatment, indicating the role of proteasomal degradation. **F**, **G** Western blot analysis of NEK2 and IFT88 expression in T24-siNC and siNEK2 cells treated with cycloheximide (CHX) for varying durations; IFT88 degradation rates were subsequently quantified. **H**, **I** Western blot analysis of NEK2 and IFT88 in T24-vector and NEK2-overexpressing cells following CHX treatment over time, with quantification of IFT88 degradation rates. **J**, **K** Co-IP and western blot assays assess ubiquitination levels of IFT88 after NEK2 knockdown or overexpression in T24 cells. **L** 293T cells were transfected with HA-tagged ubiquitin (HA-Ub) and Flag-NEK2; Co-IP and western blot analyses were performed to detect IFT88 ubiquitination in control, HA-Ub, and HA-Ub+Flag-NEK2 groups.

To elucidate the molecular mechanism by which NEK2 regulates IFT88, we performed co-immunoprecipitation (Co-IP) assays and demonstrated that NEK2 interacts with IFT88 through both endogenous and exogenous mechanisms (Figs. 5A–D and S5A, S5B). Treatment of NEK2-overexpressing T24 and 5637 cells with the proteasome inhibitor MG132 restored IFT88 protein levels (Figs. 5E and S5C), suggesting that NEK2 facilitates proteasomal degradation of IFT88. Consistently, NEK2 silencing delayed IFT88 degradation, while NEK2 overexpression accelerated its degradation (Fig. 5F–I and S5D–G). Further analysis revealed that NEK2 modulates the ubiquitination status of IFT88: NEK2 silencing decreased IFT88 ubiquitination, whereas NEK2 overexpression enhanced it (Figs. 5J, K and S5H, I). In both T24 and 5637 cells treated with the NEK2 inhibitor JH295, reduced ubiquitination of IFT88 was observed (Fig. S5J, K). Moreover, co-transfection of HA-Ub and Flag-NEK2 plasmids into 293T cells led to a marked increase in IFT88 ubiquitination (Fig. 5L). We further explored the interaction domains between NEK2 and IFT88. IFT88 mainly interacted with the NEK2 1–300 domain, whereas NEK2 interacted with both the IFT88 1–400 and 401–800 domains (Fig. S6B). Collectively, these results indicate that NEK2 promotes the ubiquitination and subsequent proteasomal degradation of IFT88, thereby regulating primary cilia dynamics in bladder cancer cells.

### AURKA functions as a downstream effector of NEK2

**Fig. 6:**
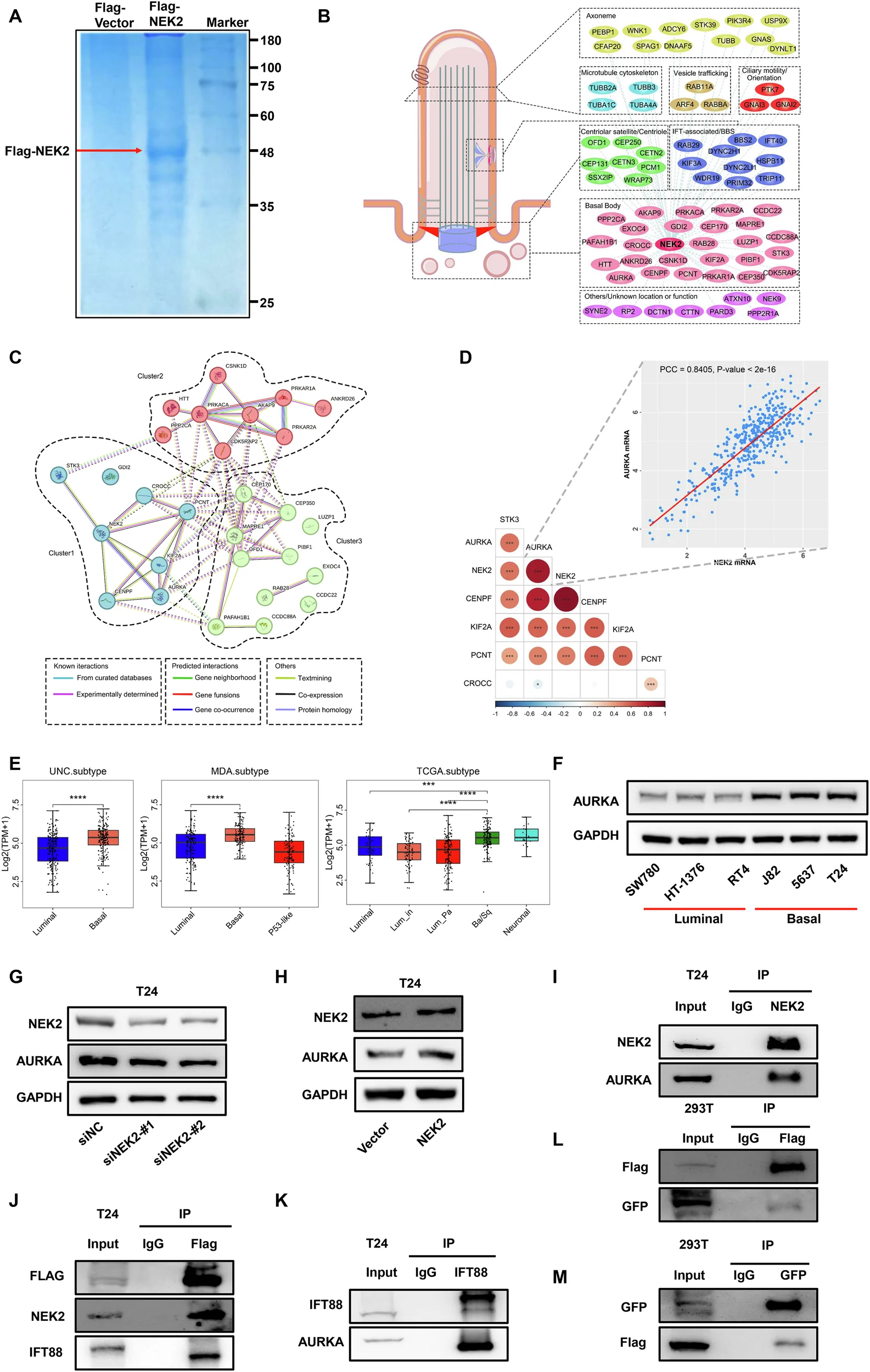
AURKA functions as a downstream effector of NEK2. **A** Coomassie Brilliant Blue staining identifies the position of Flag-NEK2 following immunoprecipitation in 293T cells transfected with Flag-NEK2 plasmid. **B** Network diagram visualizes 74 primary cilia (PC)-related proteins interacting with NEK2, grouped by function and subcellular localization within the cilium. **C** Basal body proteins interacting with NEK2 were analyzed using STRING, with K-means clustering highlighting proteins with the closest proximity to NEK2. **D** Co-expression analysis demonstrates mRNA correlations among basal body proteins interacting with NEK2. **E** Boxplots show AURKA expression across MIBC subtypes classified by UNC, MDA, and TCGA systems. **F** Western blot analysis compares AURKA expression in luminal versus basal MIBC. **G**, **H** Western blots reveal changes in AURKA expression following NEK2 silencing or overexpression in T24 cells. **I** Co-IP and Western blot assays confirm interactions between AURKA and NEK2 in T24 cells. **J** Co-IP and Western blot assays confirm interactions among Flag-tagged AURKA, IFT88, and NEK2 in T24 cells. **K** Co-IP and western blot assays confirm interactions between AURKA and IFT88 in T24 cells. **L**, **M** Co-IP and western blot analyses demonstrate interaction between GFP-tagged AURKA and Flag-tagged NEK2 in 293T cells. Statistical significance: *, *P* < 0.05; **, *P* < 0.01; ***, *P* < 0.001; ****, *P* < 0.0001.

To further delineate the mechanism by which NEK2 regulates IFT88, we performed mass spectrometry analysis (Fig. 6A), identifying 74 primary cilia (PC) proteins interacting with NEK2. These proteins were classified by subcellular localization and function-including axoneme, microtubule cytoskeleton, vesicle trafficking, ciliary motility/orientation, centriolar satellite/centriole, IFT-associated/BBS, basal body, and others [3]. Notably, 26 PC proteins and NEK2 were found to co-localize at the basal body of the primary cilium (Fig. 6B). Protein-protein interaction analysis using STRING revealed that six proteins-STK3, CROCC, PCNT, KIF2A, AURKA, and CENPF-directly interact with NEK2, as determined by K-means clustering (Fig. 6C). Co-expression analysis indicated a strong correlation between NEK2 and both AURKA and CENPF (Fig. 6D). Previous research has demonstrated that AURKA upregulation inhibits PC formation [2], and AURKA possesses dual functionality as both an E3 ubiquitin ligase and a kinase, implicating it in the ubiquitination-mediated regulation of downstream proteins [19]. These findings suggest that AURKA may modulate PC formation via ubiquitination. Further analysis revealed that AURKA is upregulated in basal-type bladder cancer (Fig. 6E), with higher expression observed in basal cell lines (J82, 5637, T24) compared to luminal lines (SW780, HT-1376, RT4) (Fig. 6F). Silencing NEK2 resulted in decreased AURKA expression, while NEK2 overexpression led to AURKA upregulation (Figs. 6G, H and S6C, D). Co-immunoprecipitation assays confirmed that AURKA, NEK2, and IFT88 interact in basal bladder cancer cells and 293T cells (Figs. 6I–M and S6E–G). Collectively, these results establish AURKA as a key downstream effector of NEK2 in the regulation of primary cilia dynamics in bladder cancer.

### NEK2 regulates primary cilia formation via AURKA

**Fig. 7:**
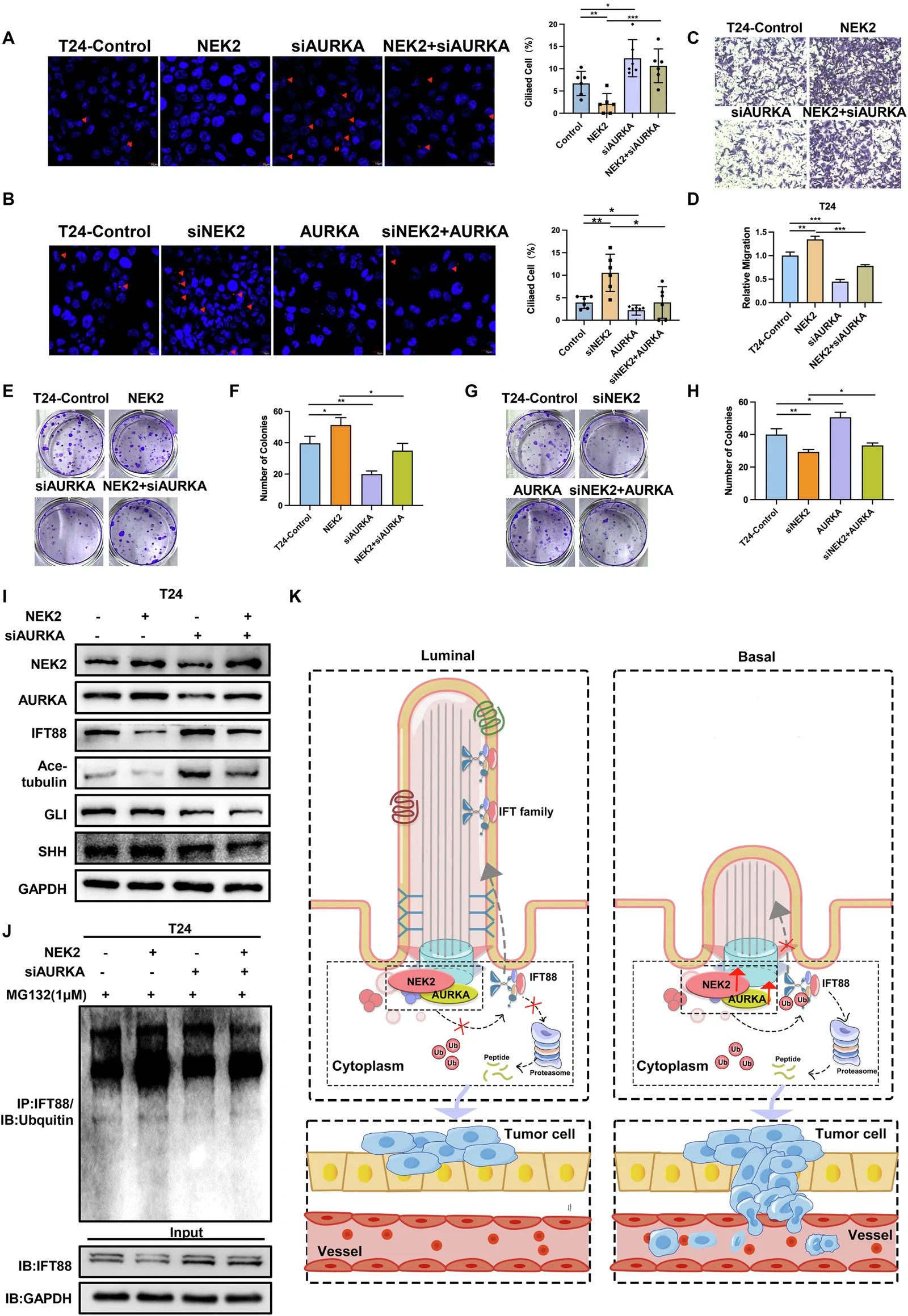
NEK2 regulates primary cilia formation and promotes tumor progression via AURKA. **A** T24-control, NEK2-overexpression, siAURKA, and NEK2-overexpression+siAURKA cells were fixed and stained for acetylated tubulin; the percentage of ciliated cells was quantified. For each experimental condition, two randomly selected non-overlapping 800× microscopic fields were analyzed from each of three independent glass coverslips, resulting in six analyzed regions per condition. The percentage of ciliated cells was calculated as the number of cells with a visible acetylated tubulin-positive cilium divided by the total number of DAPI-positive cells in the same field. **B** T24-control, siNEK2, AURKA-overexpression, and siNEK2+AURKA-overexpression cells were similarly stained and analyzed for ciliated cell percentage. For each experimental condition, two randomly selected non-overlapping 800× microscopic fields were analyzed from each of three independent glass coverslips, resulting in six analyzed regions per condition. The percentage of ciliated cells was calculated as described above. **C**, **D** Transwell assays assessed migration ability in T24-control, NEK2-overexpression, siAURKA, and NEK2-overexpression+siAURKA groups. **E**, **F** Colony formation assays evaluated cell growth in T24-control, NEK2-overexpression, siAURKA, and NEK2-overexpression+siAURKA groups. **G**, **H** Colony formation assays evaluated cell growth in T24-control, siNEK2, AURKA-overexpression, and siNEK2+AURKA-overexpression groups. **I** Western blot analysis of SHH pathway markers (GLI and SHH), acetylated tubulin, IFT88, NEK2, and AURKA in T24-control, NEK2-overexpression, siAURKA, and NEK2-overexpression+siAURKA groups. **J** Co-IP and western blot assays determined ubiquitination levels and expression of IFT88 in T24-control, NEK2-overexpression, siAURKA, and NEK2-overexpression+siAURKA groups. **K** Schematic diagram illustrating the NEK2/AURKA/IFT88 axis and its regulation of primary cilia and SHH pathway activation in promoting MIBC progression. Statistical significance: *, *P* < 0.05; **, *P* < 0.01; ***, *P* < 0.001; ****, *P* < 0.0001.

To clarify the functional interplay between NEK2 and AURKA in the regulation of primary cilia, we silenced AURKA in NEK2-overexpressing cells and observed that the NEK2-induced degradation of primary cilia was reversed by AURKA depletion (Figs. 7A and S7A, B). Conversely, the promotion of cilia formation resulting from NEK2 knockdown was abrogated by AURKA overexpression (Figs. 7B and S7C, D). Functionally, NEK2-driven tumor progression was suppressed by AURKA knockdown, while the inhibitory effects of NEK2 silencing on cell growth were rescued by AURKA overexpression (Figs. 7C–H and S7E–J). The suppression of IFT88 and Ace-tubulin expression by NEK2 was reversed upon AURKA knockdown. Furthermore, NEK2 also promoted the expression of SHH pathway components (SHH and GLI), an effect that was counteracted by AURKA depletion (Figs. 7I and S7K). Mechanistically, NEK2-mediated upregulation of IFT88 ubiquitination was inhibited by AURKA silencing (Figs. 7J and S7L), indicating that NEK2/AURKA axis regulates primary cilia formation through ubiquitin-dependent modification of IFT88. In summary, NEK2 and AURKA cooperate to inhibit primary cilia formation via ubiquitination of IFT88, thereby activating the SHH pathway and driving basal bladder cancer cell progression (Fig. 7K).

## Discussion

Bladder cancer is a highly heterogeneous malignancy characterized by distinct pathological and molecular features [20]. Traditional therapeutic approaches, which are primarily based on clinical and pathological staging, are limited by their lack of individualized precision. Recent advances in high-throughput sequencing technologies have enabled comprehensive molecular subtyping based on gene expression profiles, substantially deepening our understanding of bladder cancer biology. Initial studies by Bamrauer et al. employed consensus clustering across four gene expression array datasets, identifying two principal subtypes: basal-like and luminal-like [21]. Subsequent efforts have expanded these classifications, with The Cancer Genome Atlas (TCGA) defining luminal-papillary, luminal-infiltrated, luminal, basal/squamous, and neuronal subtypes; the MD Anderson (MDA) group identifying basal, luminal, and p53-like subtypes; and the Lund cohort delineating UroA-Prog, UroC, Uro-Inf, UroB, GU, GU-Inf, Ba/Sq-Inf, Ba/Sq, Mes-like, and Sc/NE-like subtypes, among others [22–24]. To harmonize these diverse schemes, a consensus classification was proposed, merging existing clusters and categorizing muscle-invasive bladder cancer (MIBC) into six distinct groups: luminal papillary, luminal nonspecified, luminal unstable, stroma-rich, basal/squamous, and neuroendocrine-like [14]. This framework provides a robust basis for the validation and application of predictive biomarkers in future prospective clinical trials.

Notably, all major classification systems converge on the identification of two core subtypes-luminal and basal-which together account for approximately 70% of MIBC cases. Emerging evidence suggests that these molecular subtypes are associated with differential therapeutic responses. For instance, the efficacy of Enfortumab Vedotin (EV) is mediated by NECTIN4 expression, which is predominantly elevated in luminal bladder cancer [16]. Similarly, RC48, an ERBB2-targeted agent, demonstrates enhanced tumor inhibition in luminal subtypes due to high ERBB2 expression [17]. Furthermore, comparative studies indicate that patients with basal tumors may derive greater benefit from neoadjuvant chemotherapy (NAC) [15]. Despite these advances, significant challenges remain in translating molecular subtyping into routine clinical management, primarily due to the lack of prospective validation. Consequently, integrating molecular subtype information into clinical decision-making for bladder cancer will require further research and validation before widespread adoption.

Although primary cilia (PC) play dual roles in different types of cancer, PC deficiency has been observed across multiple malignancies and is increasingly recognized as a therapeutic opportunity, particularly through restoration of PC function. In our previous study, we found that PC deletion occurs in bladder cancer-especially muscle-invasive bladder cancer (MIBC)-and that restoring PC significantly suppresses tumor proliferation [3]. Therefore, investigating PC formation and loss across MIBC molecular subtypes is essential. Although six molecular subtypes have been defined, basal and luminal types comprise the majority of MIBC cases, and most bladder cancer cell lines are classified as either basal or luminal [25]. Accordingly, our research focuses on the relationship between PC and these two subtypes.

Using immunofluorescence staining, we quantified the proportion of ciliated cells and discovered that PC loss predominates in basal-type bladder cancer. To further investigate the functional impact of PC deficiency, we established PC deletion and restoration models by silencing or overexpressing IFT88, a key ciliary transport protein. Overexpression of IFT88 inhibited MIBC progression, indicating that PC formation suppresses basal-type bladder cancer [26]. To further elucidate the basis of PC loss in basal bladder cancer, we performed WGCNA and identified 14 ciliary genes associated with the basal subtype. Among these, we also found NEK2 as highly expressed and strongly correlated with basal-type bladder cancer. NEK2 is a multifunctional protein involved in cell cycle regulation, spindle assembly checkpoint, phosphorylation of downstream targets, microtubule stabilization, and primary cilia assembly [27, 28]. As a central regulator of cilia biogenesis and resorption, NEK2 overexpression accelerates ciliary resorption [29], and it promotes cilia disassembly via Kif24-mediated microtubule depolymerization [28]. Our data suggest that NEK2 overexpression is a principal cause of PC degradation in basal-type bladder cancer. Silencing NEK2 not only promoted PC formation but also inhibited tumor progression. Pharmacological inhibition of NEK2 further validated its role: INH1, a HEC1/NEK2 interaction inhibitor [30], and JH295, a selective NEK2 kinase inhibitor [31], were tested. Notably, basal-type cells were more sensitive to JH295 than luminal-type cells, while both types showed similar responses to INH1, suggesting that NEK2-specific inhibition may offer clinical benefit for basal-type bladder cancer, rather than non-specific inhibitor. Mechanistically, NEK2 suppresses PC formation by regulating IFT88 expression. We explored the NEK2-IFT88 relationship and found that primary cilia are ubiquitous organelles [32] whose assembly and degradation are influenced by ubiquitination. NEK2 overexpression led to IFT88 degradation, which was reversed by the proteasome inhibitor MG132, indicating that NEK2 regulates IFT88 through ubiquitination modification. Co-immunoprecipitation assays confirmed that NEK2 enhances ubiquitination level of IFT88, while silencing NEK2 downregulates it. These results suggest that NEK2 inhibits primary cilia formation by promoting the ubiquitination-dependent degradation of IFT88.

To identify the E3 ubiquitin ligase mediating IFT88 ubiquitination, we performed mass spectrometry and bioinformatics analysis. We found that AURKA interacts and co-localizes with NEK2 at the basal body of primary cilia, with the highest mRNA correlation between the two. AURKA, a serine/threonine kinase, regulates diverse substrates through phosphorylation [33], and recent evidence indicates that it also functions as an E3 ubiquitin ligase [19]. AURKA is overexpressed in bladder cancer and promotes PC degradation [2]. Our data support that AURKA acts downstream of NEK2, interacting with both NEK2 and IFT88. Knockdown of AURKA reversed the malignant phenotype and IFT88 ubiquitination induced by NEK2 overexpression, indicating that the NEK2/AURKA axis inhibits IFT88 expression through ubiquitination-dependent regulation, suppresses PC formation, and promotes the proliferation and migration of basal bladder cancer cells.

In summary, PC loss is a hallmark of basal-type bladder cancer and its restoration inhibits tumor progression. NEK2 functions as an oncogene driving PC degradation in basal-type bladder cancer, and the NEK2-specific inhibitor JH295 preferentially suppresses basal-type cell viability. The NEK2/AURKA axis promotes IFT88 ubiquitination and downregulation, thereby degrading PC. Our findings present a novel therapeutic strategy for basal-type bladder cancer by restoring primary cilia and elucidate the mechanism by which PC regulates tumor biology in this subtype.

## Materials and methods

### Publicly available data collection and analysis

The mRNA expression profiles for muscle-invasive bladder cancer (MIBC) were retrieved from The Cancer Genome Atlas (TCGA) via the UCSC Xena platform (http://xena.ucsc.edu/), with gene expression values normalized and transformed to Log2(TPM + 1) units. Consensus classification of MIBC into six molecular subtypes-basal/squamous (Ba/Sq), luminal unstable (LumU), luminal nonspecified (LumNS), luminal papillary (LumP), neuroendocrine-like (NE-like), and stroma-rich-was performed according to established criteria [14]. Additional subtype assignments (UNC, TCGA, and MDA) were determined using the BLCAsubtyping R package [14]. Differential gene expression analysis was conducted by extracting mRNA levels for selected genes and comparing subtype-specific expression using t-tests. For broader differential expression analysis, tumor profiles were matched to adjacent normal tissues, and the limma R package was used to identify differentially expressed genes (DEGs) with thresholds of |logFC| > 1 and adjusted *P*-value < 0.05.

Weighted gene coexpression network analysis (WGCNA) was employed to delineate co-expression modules among DEGs, utilizing Pearson correlation to construct similarity matrices. Networks were generated using a power value (*β* = 8) to optimize connectivity and scale-free topology (correlation between p(k) and *k* > 0.85). Hierarchical clustering of the 1-TOM matrix facilitated module identification, and module eigengenes were correlated with clinical features, including the six consensus subtypes. Modules were considered significantly associated with subtypes if *R* > 0.4 or *R* ≤ −0.4.

Gene dependency scores (gDS) were incorporated from previous studies [34], with gDS < 0 indicating gene essentiality for cell viability and gDS > 0 suggesting dispensability. This metric was used to assess the functional importance of NEK2 in bladder cancer cells. For survival analysis, samples were stratified into high and low expression groups using optimal cut-off values determined by the surv_cutpoint function in R, and Kaplan-Meier survival curves were generated to compare survival probabilities.

Protein-protein interaction networks were constructed by submitting gene sets to the STRING database (https://string-db.org), selecting “Homo sapiens” as the organism, and applying K-means clustering to visualize network architecture. For co-expression analysis, mRNA expression levels were extracted, and inter-gene correlations were calculated using the Hmisc R package and the Pearson method.

### Antibodies

Antibodies were sourced as follows: anti-NEK2 (24171-1-AP, Proteintech Group, 1:1000; 66632-1-Ig, Proteintech Group, 1:1000), anti-Ubiquitin (3933, Cell Signaling Technology, 1:1000; 10201-2-AP, Proteintech Group, 1:1000), anti-IFT88 (13967-1-AP, Proteintech Group, 1:1000), anti-Acetylated tubulin (T6793, Sigma-Aldrich, 1:1000), anti-GAPDH (60004-1-Ig, Proteintech Group, 1:1000), anti-KRT20 (17329-1-AP, Proteintech Group, 1:1000), anti-GATA3 (66400-1-Ig, Proteintech Group, 1:1000), anti-CD44 (15675-1-AP, Proteintech Group, 1:1000), anti-KRT5/6 (ab17133, Abcam, 1:1000), anti-Flag (20543-1-AP, Proteintech Group, 1:1000), anti-AURKA (91590, Cell Signaling Technology, 1:1000), anti-GFP (50430-2-AP, Proteintech Group, 1:1000), anti-GLI (66905-1-Ig, Proteintech Group, 1:1000), anti-SHH (20697-1-AP, Proteintech Group, 1:1000), HRP-conjugated Goat Anti-Mouse IgG(H + L) (SA00001-1, Proteintech, 1:3000), HRP-conjugated Goat Anti-Rabbit IgG(H + L) (SA00001-2, Proteintech, 1:3000), ARL13B (17711-1-AP, Proteintech, 1:1000), Rhodamine (TRITC)–conjugated Goat Anti-Rabbit IgG(H + L) (SA00007-2, Proteintech Group, 1:500), and FITC Monoclonal antibody (68132-1-Ig, Proteintech Group, 1:1000).

### Reagents, kits, and plasmids

Chemicals, kits, and reagents were obtained from the following suppliers: Puromycin (ant-pr-1, Invivogen), cell lysis buffer for Western blotting (P0013, Beyotime), Pierce™ Classic Magnetic IP/Co-IP Kit (88804, ThermoFisher Scientific), Lipo8000™ Transfection Reagent (C0533-1.5 ml, Beyotime), MG132 (HY-13259, MedChemExpress), human NEK2 plasmids (OBiO Technology (Shanghai) Corp., Ltd), c-Myc tag plasmid (OBiO Technology (Shanghai) Corp., Ltd), Flag tag plasmid (OBiO Technology (Shanghai) Corp., Ltd), GFP tag plasmid (OBiO Technology (Shanghai) Corp., Ltd), HA tag plasmid (OBiO Technology (Shanghai) Corp., Ltd), and cycloheximide (HY-12320, MedChemExpress).

### Cell culture

Luminal (SW780, RT4, HT1376) and basal (T24, 5637, J82) bladder cancer cell lines, as well as 293T cells, were purchased from Procell Life Science & Technology Co. Ltd. (Wuhan, China). All cell lines were authenticated by short tandem repeat (STR) profiling and confirmed to be free of mycoplasma contamination before use. According to the manufacturer’s protocols, T24 and 5637 cells were cultured in 1640 medium (GE Healthcare Life Sciences SH30027.0); J82 and HT1376 cells in MEM medium (Procell, PM150410); RT4 cells in McCoy’s 5A medium (Procell, PM150710); and SW780 and 293T cells in DMEM medium (Procell, PM150210). All media were supplemented with 10% fetal bovine serum (FBS; Biological Industries 04-001-1) and 1% Penicillin/Streptomycin (Cienry CR-15140). Cells were maintained in a humidified incubator at 37 °C with 5% CO₂.

### Immunohistochemistry

Formalin-fixed, paraffin-embedded (FFPE) muscle-invasive bladder cancer (MIBC) tissue biopsy specimens were collected from the Second Hospital of Tianjin Medical University. The study involving human specimens was approved by the Ethics Committee of the Second Hospital of Tianjin Medical University (approval no. KY2023K063), and written informed consent was obtained from all patients. All procedures involving human participants were conducted in accordance with the Declaration of Helsinki. Luminal markers (GATA3 and KRT20) and basal markers (KRT5/6 and CD44) were assessed in 100 MIBC samples to classify tumors into luminal and basal subtypes. Based on marker expression, 32 luminal and 39 basal bladder cancer tissues were identified, while the remaining cases were excluded from subtype-specific analysis because they showed overlapping expression of luminal and basal markers. Owing to limited serial FFPE material after subtype-marker staining, primary cilia immunofluorescence was performed on a subset of cases with sufficient remaining sections and unequivocal subtype assignment, including 8 luminal and 8 basal tumors. These cases were selected based on tissue availability and clear subtype assignment, rather than on the level of cilia staining. Immunostaining for NEK2 was then performed on the 71 subtype-classified MIBC specimens. Sections were deparaffinized and rehydrated, followed by antigen retrieval in sodium citrate buffer and blocking with 3% H_2_O_2_ and avidin/biotin. Specimens were incubated with primary antibodies overnight, then with appropriate secondary antibodies. Diaminobenzidine (DAB) was used as the chromogen, and hematoxylin was applied for counterstaining. Immunohistochemistry (IHC) staining was quantified using ImageJ software, with scores calculated by multiplying staining intensity (0, negative; 1, weak; 2, moderate; 3, strong) by the proportion of immunopositive cells (1, <25%; 2, 25–50%; 3, 50–75%; 4, >75%).

### Immunoblotting and immunoprecipitation

For immunoblotting, treated cells were lysed in RIPA buffer (Boster, Wuhan) supplemented with 10% phenylmethanesulfonyl fluoride (PMSF, Solarbio, Beijing) for 30 min on ice. Lysates were centrifuged at 12,000 × *g* for 15 min, and the supernatant containing soluble proteins was collected. Protein concentrations were determined using a bicinchoninic acid (BCA) assay (Solarbio, Beijing). Samples were heated to 95 °C in NuPAGE LDS sample buffer (4×) (Solarbio, Beijing) for 5 min, separated by SDS-PAGE, and transferred onto PVDF membranes (ThermoFisher, China). Membranes were blocked with 5% skimmed milk and incubated with primary antibodies overnight at 4 °C. Bound antibodies were detected with HRP-conjugated secondary antibodies and visualized using Immobilon™ Western Chemiluminescent HRP Substrate. Immunoreactive bands were detected with the ECL Western blotting detection system.

Co-immunoprecipitation (Co-IP) assays were performed according to the ThermoScientific Pierce Co-IP Kit (88804) protocol. Treated cells were lysed in ice-cold IP Lysis/Wash Buffer containing 1% PMSF and incubated on ice for 5 minutes. Lysates were centrifuged at 13,000 × *g* for 15 min, and the supernatant was collected. After protein quantification by BCA assay, 1000 μg of total protein was incubated with 2–5 μg of primary antibody overnight at 4 °C with gentle agitation, followed by incubation with 25 μl Pierce Protein A/G Magnetic Beads for 1–2 h at room temperature. Immunocomplexes were collected using a magnet, washed three times with IP Lysis/Wash Buffer, resuspended in Lane Marker Sample Buffer (5×), boiled for 5 min at 95 °C, and subjected to SDS-PAGE and immunoblotting analysis.

### Drug dose–response curve and CCK-8 assay

Cells were seeded into 96-well plates in quintuplicate or sextuplicate. After 24 h, the NEK2 inhibitor JH295 (HY-116423, MedChemExpress) and INH1 (HY-16660, MedChemExpress) was added at various concentrations and incubated for 48 h. CCK-8 reagent (APE×BIO, USA) was then added at a 10:1 ratio and incubated for 2–4 h. Cell viability was assessed by measuring optical density at 450 nm, and IC50 values for each group were calculated using GraphPad Prism 10 software.

### Colony formation assay

Cells were seeded into 12-well plates at a density of 500 cells per well and cultured in the appropriate medium supplemented with 10% FBS for 10–14 days. Colonies were then fixed with paraformaldehyde and stained with crystal violet. The number of colonies was quantified using ImageJ software. All experiments were performed in triplicate or more.

### Immunofluorescence

Cells were seeded onto three glass coverslips (15 mm diameter) and fixed in 4% paraformaldehyde (PFA) for 10 min at room temperature. After fixation, cells were washed three times with PBS (5 min each), permeabilized with 0.5% Triton X-100 for 5 min, and blocked with 1% bovine serum albumin (BSA) in PBS for 10–15 min. Cells were then incubated overnight at 4 °C with ciliary marker antibodies against acetylated tubulin (1:500 dilution, Sigma-Aldrich) in PBS, followed by incubation with TRITC-labeled anti-mouse secondary antibody (1:200 dilution, Southern Biotech) for 2 h. Nuclei were counterstained with 4′6-diamidino-2-phenylindole (DAPI, Solarbio). For each experimental condition of cells, two randomly selected non-overlapping 800× microscopic fields were analyzed from each of three independent glass coverslips, resulting in six analyzed regions per condition. The percentage of ciliated cells was calculated as the number of cells with a visible acetylated tubulin-positive cilium divided by the total number of DAPI-positive cells in the same field. For human tissue immunofluorescence, 8 luminal and 8 basal MIBC cases were analyzed as described above. For each tissue sample, two randomly selected non-overlapping microscopic fields were evaluated, and the average percentage of ciliated cells was calculated. For mouse tissue immunofluorescence, the average percentage of ciliated cells was similarly calculated from two randomly selected non-overlapping fields per sample.

### Wound healing assay

Cells were seeded into 6-well plates at a density of 1 × 10^5^ cells per well and incubated for 24 h. Upon reaching 80% confluence, linear wounds were created in the cell monolayer using 1000 μl pipette tips. Cells were then washed with PBS and cultured in their respective medium supplemented with 1% FBS. Images of the same wound sites were captured at 0, 12, 24, and 48 h to monitor wound closure.

### Transfection

T24 cells were used as the primary basal-like bladder cancer model for mechanistic perturbation experiments because they showed relatively high endogenous NEK2 expression, low baseline ciliogenesis, and stable performance in transfection-based assays. Key findings were further validated in 5637 cells. T24 and 5637 cells were seeded into 6-well plates (2 × 105 cells per well) and incubated overnight. Cells were then transfected with 60 nM siRNA (GenePharma) using RNAi-Mate (GenePharma) according to the manufacturer’s instructions. The siRNA sequences were as follows: siNEK2-#1, sense 5′-GAUGCAAUUUGGUCAUUAATT-3′, antisense 5′-UUA AUGACCAAAUUGCAU CTT-3′; siNEK2-#2, sense 5′-GAAAGGCAAUACUUAGAUGTT-3′, antisense 5’-CAUCUAAGUAUUGCCUUUCTT-3′; siAURKA, sense 5′-GCAGAG AACUGC UACUUAUTT-3′, antisense 5′-AUAAGUAGCAGUUCUCUGCTT-3′; siIFT88, sense 5′-GCCAUUAAAUUC UACCGAATT-3′, antisense 5′-UUCGGUAGAAUUUAAUGGCTT-3′.

For in vitro overexpression experiments, the appropriate plasmids were transfected into T24 and 5637 cells using Lipo8000™ Transfection Reagent (Beyotime) according to the manufacturer’s instructions. Cells were cultured to approximately 50% confluence, and the medium was replaced with Opti-MEM before transfection. After 24 h, the medium was replaced with fresh complete medium, and overexpression efficiency was confirmed by western blotting. For xenograft experiments, stable IFT88-overexpressing T24 cells were generated using lentiviral constructs purchased from OBiO Technology (Shanghai) Corp., Ltd. Stable cells were selected with 10 μg/ml puromycin (InvivoGen, San Diego, CA, USA) for 1 week, and overexpression efficiency was confirmed by western blotting before injection.

### Animal care

Four- to six-week-old male Balb/c nude mice (24–26 g; HFK Bioscience Co. Ltd., Beijing, China) were used for xenograft tumor growth assays. Mice were housed in a specific-pathogen-free (SPF) environment at the Experimental Animal Center of the Second Hospital of Tianjin Medical University. All animal experimental protocols were approved by the Institutional Animal Care and Use Committee of Yi Shengyuan Gene Technology (Tianjin) Co., Ltd. (approval no. YSY-DWLL-2025623). All methods involving animals were performed in accordance with the approved protocol and all relevant guidelines and regulations. Efforts were made throughout the study to minimize or prevent animal suffering and to ensure optimal welfare.

### Animal procedures

Mice were randomly assigned to experimental groups to minimize selection bias. T24-IFT88 overexpression cells and T24-WT cells were seeded at a density of 1 × 10⁶ cells in 100 μl serum-free 1640 medium and injected subcutaneously into 4- to 6-week-old nude mice (*n* = 6 per group). Tumor incidence and growth were monitored, with tumor size measured using calipers. Tumor-free survival was analyzed using Kaplan–Meier survival curves. Mice were euthanized by cervical dislocation at the experimental endpoint (4 weeks), or earlier if humane endpoint criteria were met, such as when the maximum tumor diameter reached 1.5 cm. Tumor tissues were then immediately harvested for subsequent analyses. The tumor tissues were fixed in 10% neutral buffered formalin, paraffin-embedded, and sectioned for immunohistochemistry (IHC) and immunofluorescence analyses.

For combination treatment studies, T24-WT and T24-IFT88 cells were seeded at a density of 1 × 10^6^ cells in 100 μl serum-free 1640 medium, with or without pretreatment with NEK2 inhibitor (JH295, HY-116423, MedChemExpress; 2 μM) for 24 h. Cells were injected subcutaneously into 4- to 6-week-old nude mice (*n* = 6 per group). Tumor incidence and growth were recorded, and tumor-free survival was assessed using Kaplan–Meier survival curves across the four experimental groups. Mice in which no palpable tumor was observed during the monitoring period were excluded from tumor weight and volume analyses but were retained in tumor incidence and tumor-free survival analyses.

### Statistical analyses

Statistical analyses were conducted using RStudio and GraphPad Prism version 10.0 (GraphPad Inc). Differences between two groups were assessed using Student’s *t*-test. Correlations between variables were evaluated with the Pearson method. Survival curve differences were analyzed using the Kaplan–Meier method and log-rank test. A *P*-value < 0.05 was considered statistically significant. No formal blinding was applied during the experiments or outcome assessment. Data are presented as mean ± SD unless otherwise indicated. For comparisons analyzed by Student’s *t*-test, data were treated as approximately normally distributed with comparable variance; formal normality and equal-variance tests were not performed. No formal a priori power calculation was performed; sample sizes were chosen based on prior similar studies and experimental feasibility, with exact *n* reported in the Methods and figure legends.

### Ethics approval and consent to participate

The study involving human specimens was approved by the Ethics Committee of the Second Hospital of Tianjin Medical University (approval no. KY2023K063), and written informed consent was obtained from all patients. All procedures involving human participants were conducted in accordance with the Declaration of Helsinki. All animal experimental protocols were approved by the Institutional Animal Care and Use Committee of Yi Shengyuan Gene Technology (Tianjin) Co., Ltd. (approval no. YSY-DWLL-2025623). All methods involving animals were performed in accordance with the approved protocol and all relevant guidelines and regulations.

## Supplementary information

original data

Supplemental materials

## Data Availability

The datasets analyzed in this study are publicly available. TCGA-BLCA/MIBC data were obtained from the UCSC Xena platform. No new sequencing or omics datasets were generated in this study. All other data are included in the article and supplementary materials.

## Code availability

Custom code used for bioinformatic analyses is available from the corresponding author upon reasonable request.
