# Supplementary material for: NEK2-AURKA axis drives ubiquitin-dependent IFT88 degradation to suppress ciliogenesis and promote basal-type bladder cancer
Source: Cell Death Dis. 2026 Aug 22;17(1):903. doi: 10.1038/s41419-026-09203-0 (PMC13690740; doi:10.1038/s41419-026-09203-0)

Figure 1I

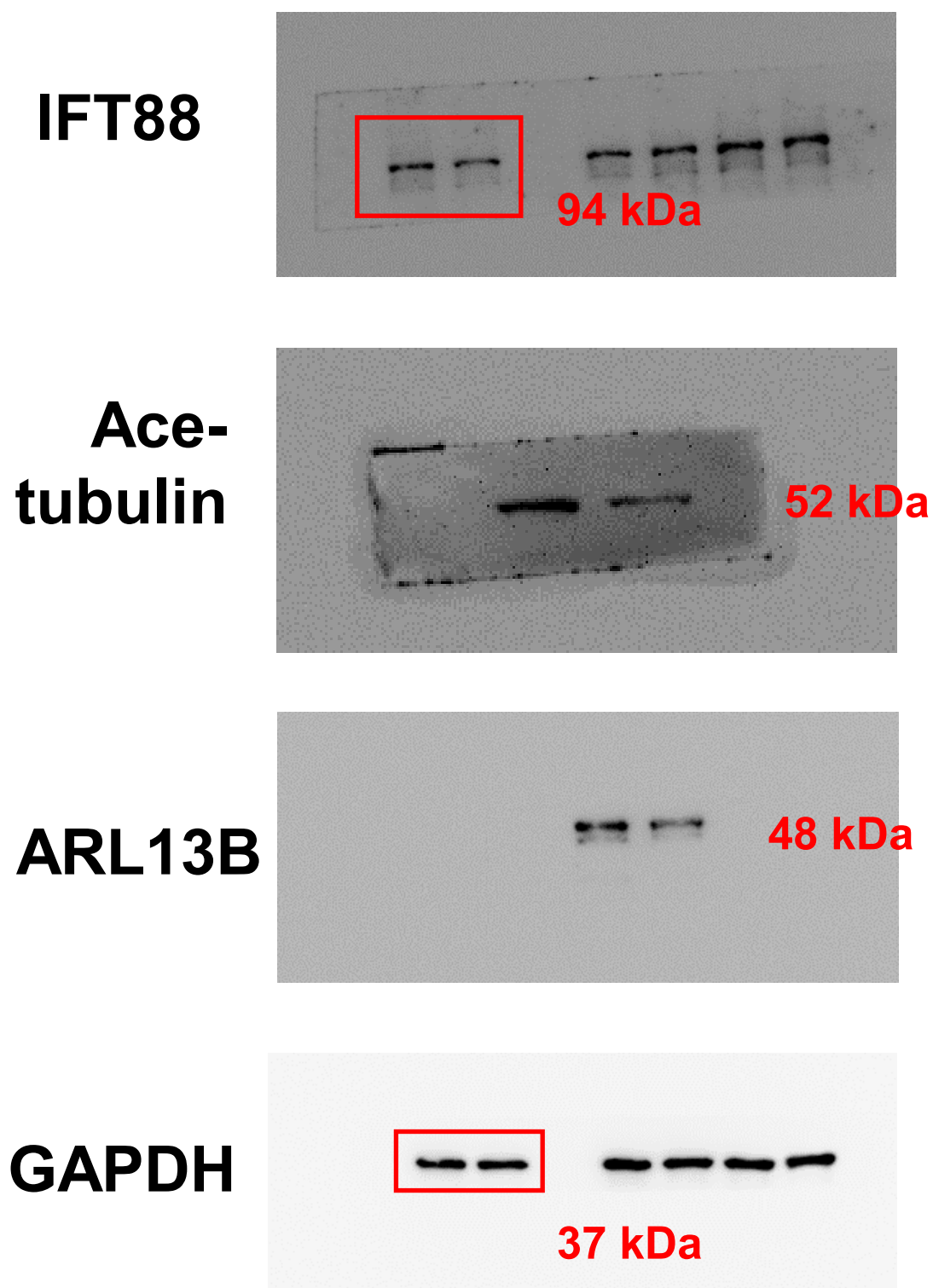

Figure 1J

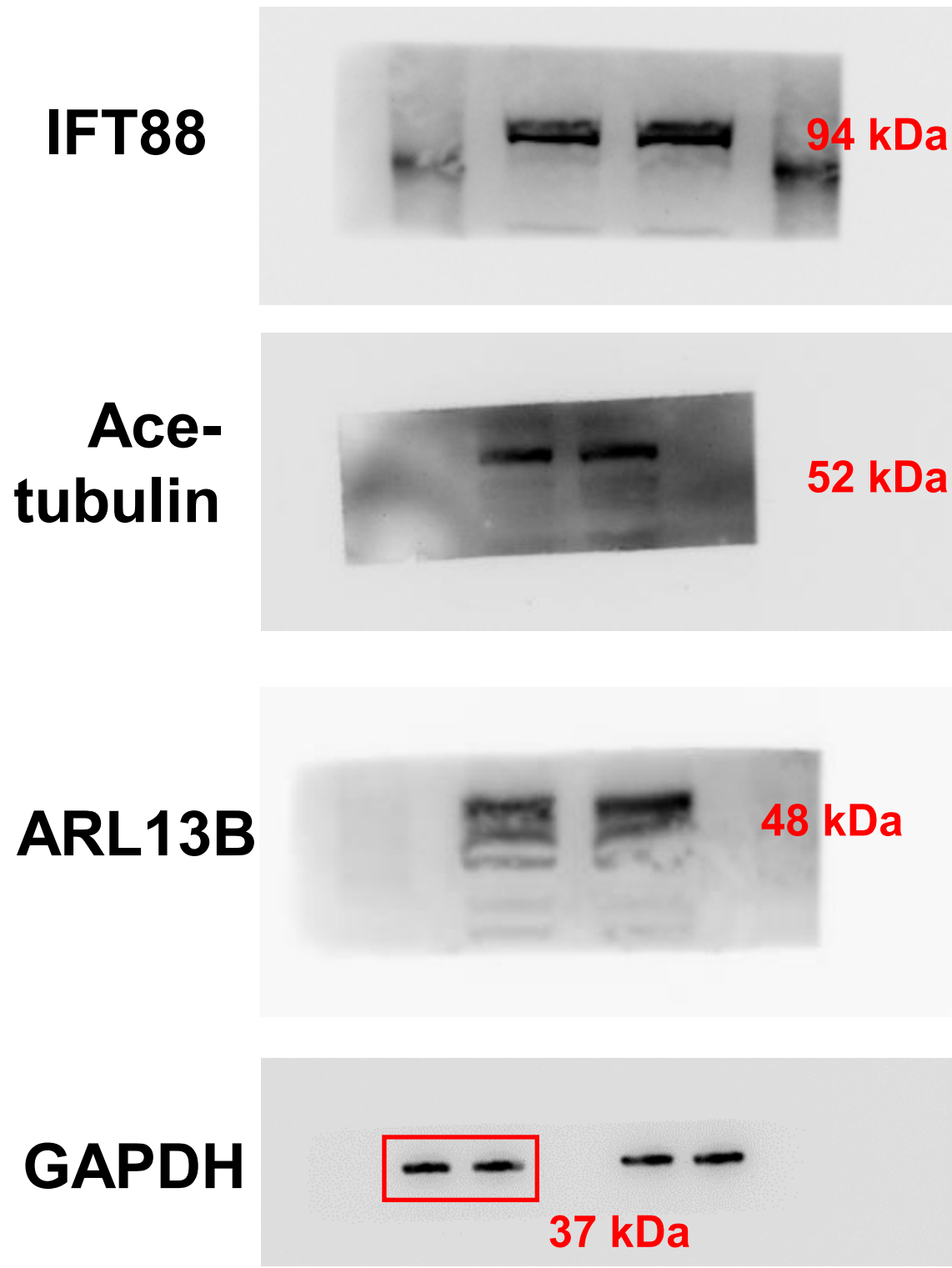

Figure 2M

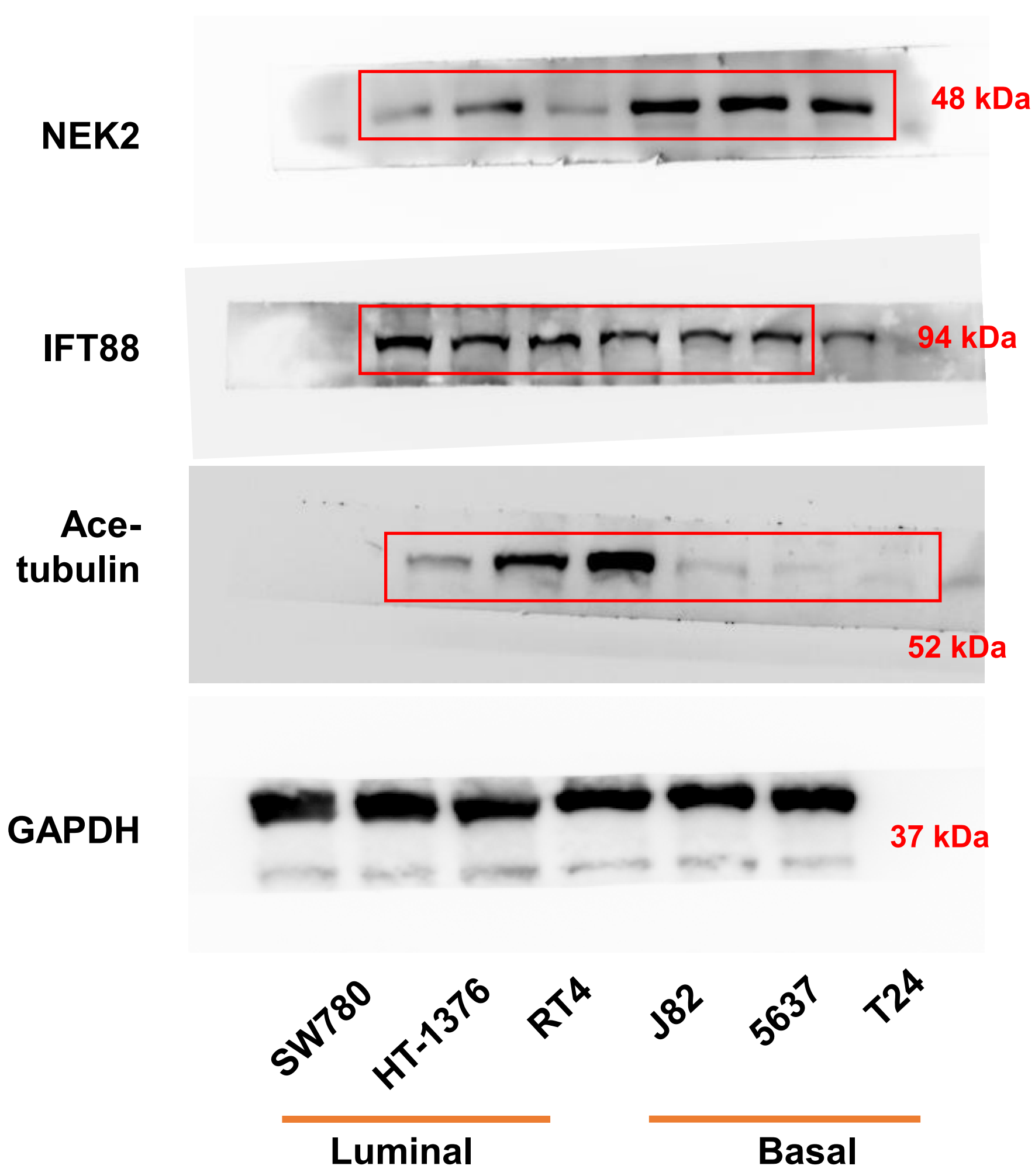

Figure 3A

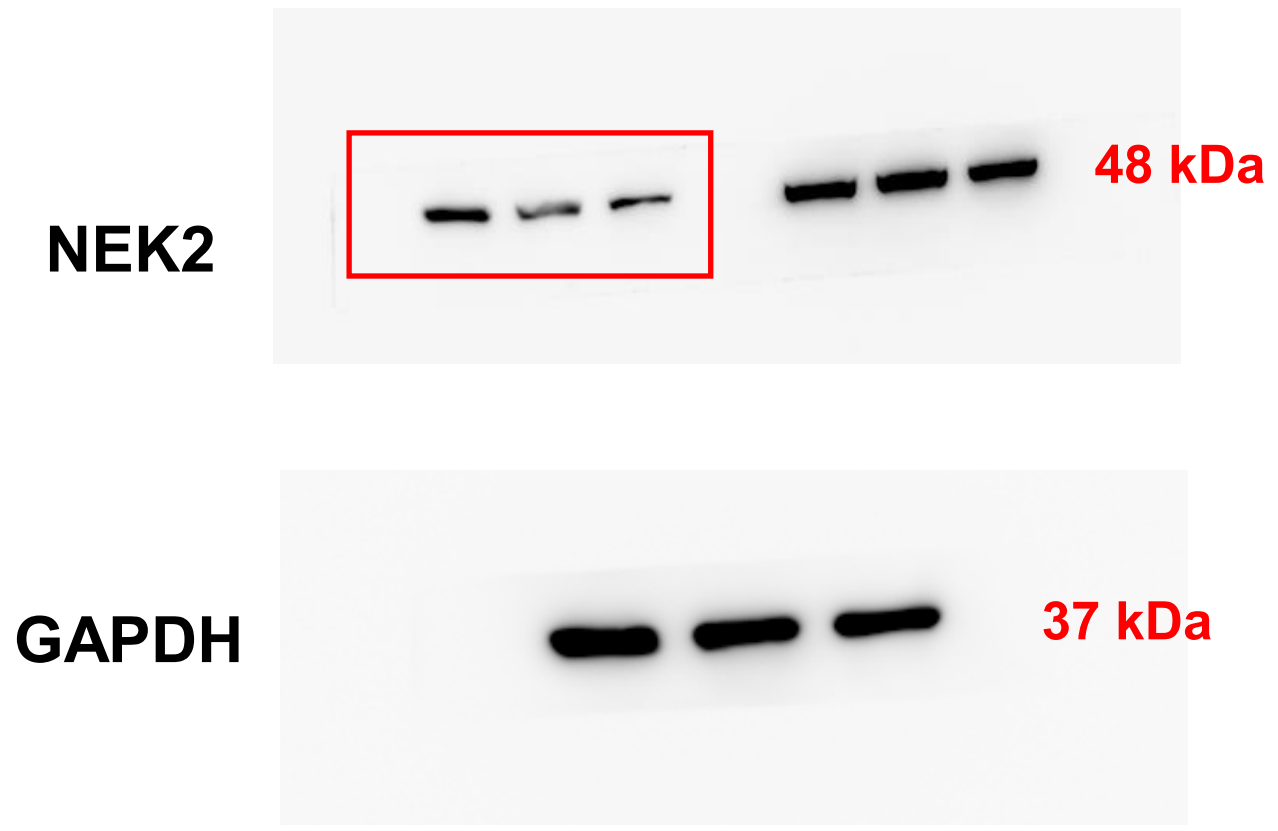

Figure 3H

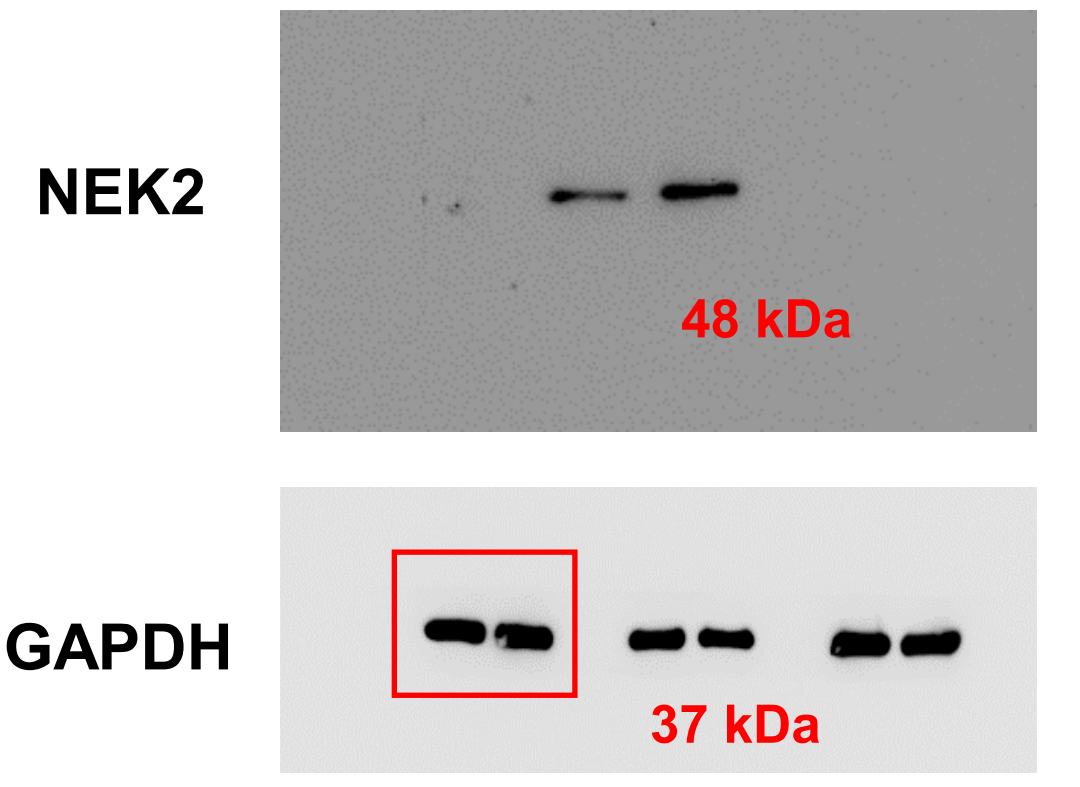

Figure 3G

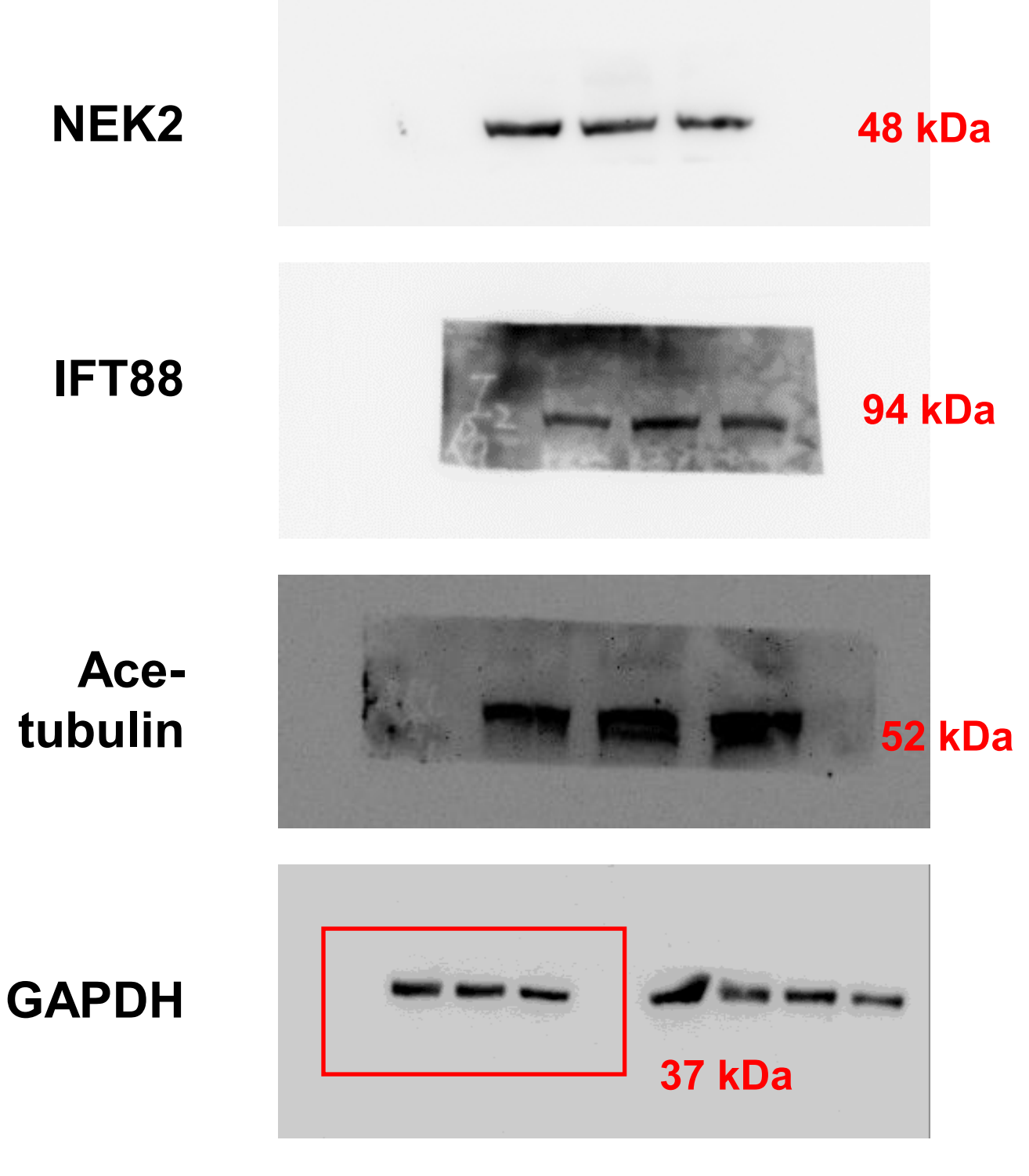

Figure 3N

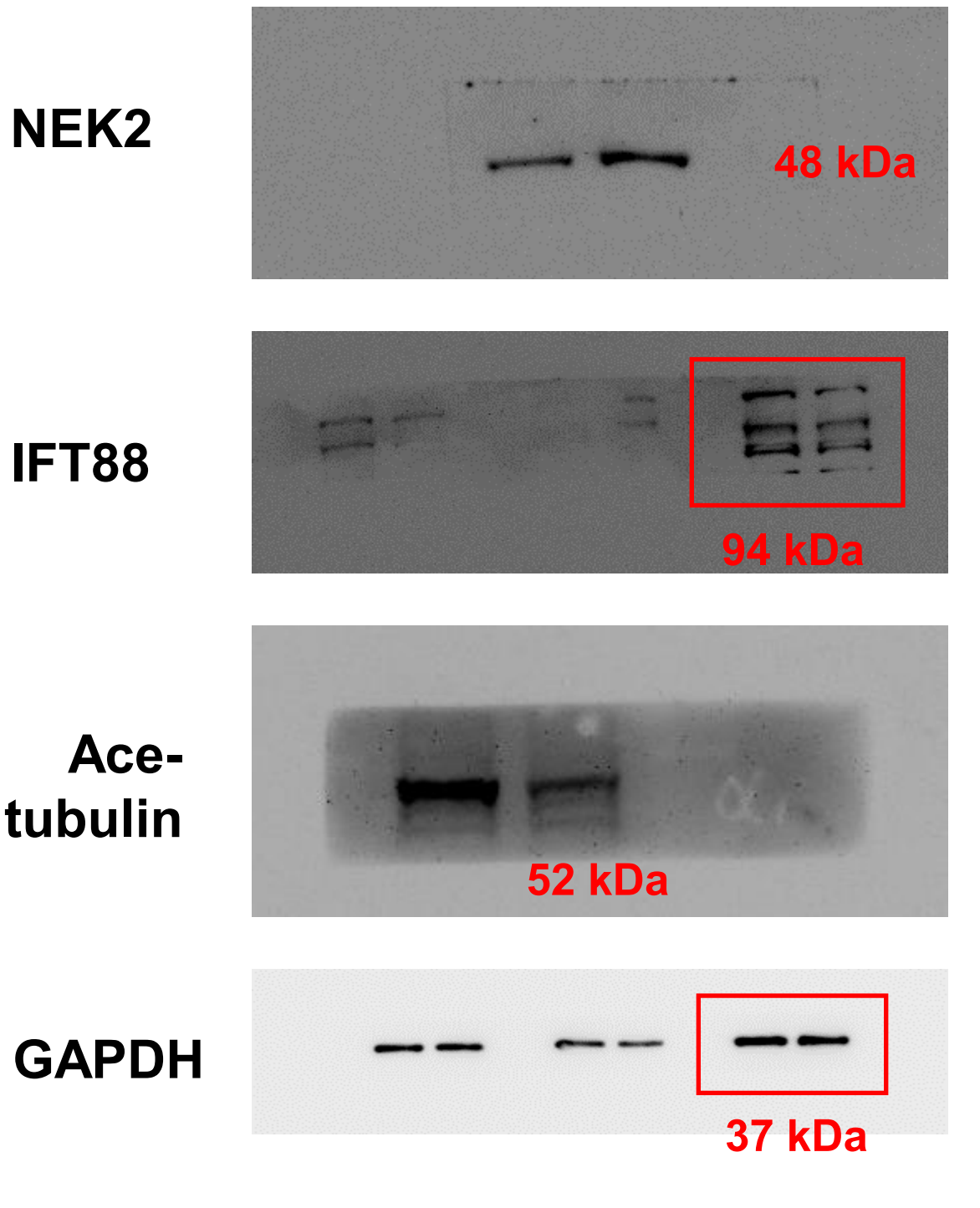

Figure 4B

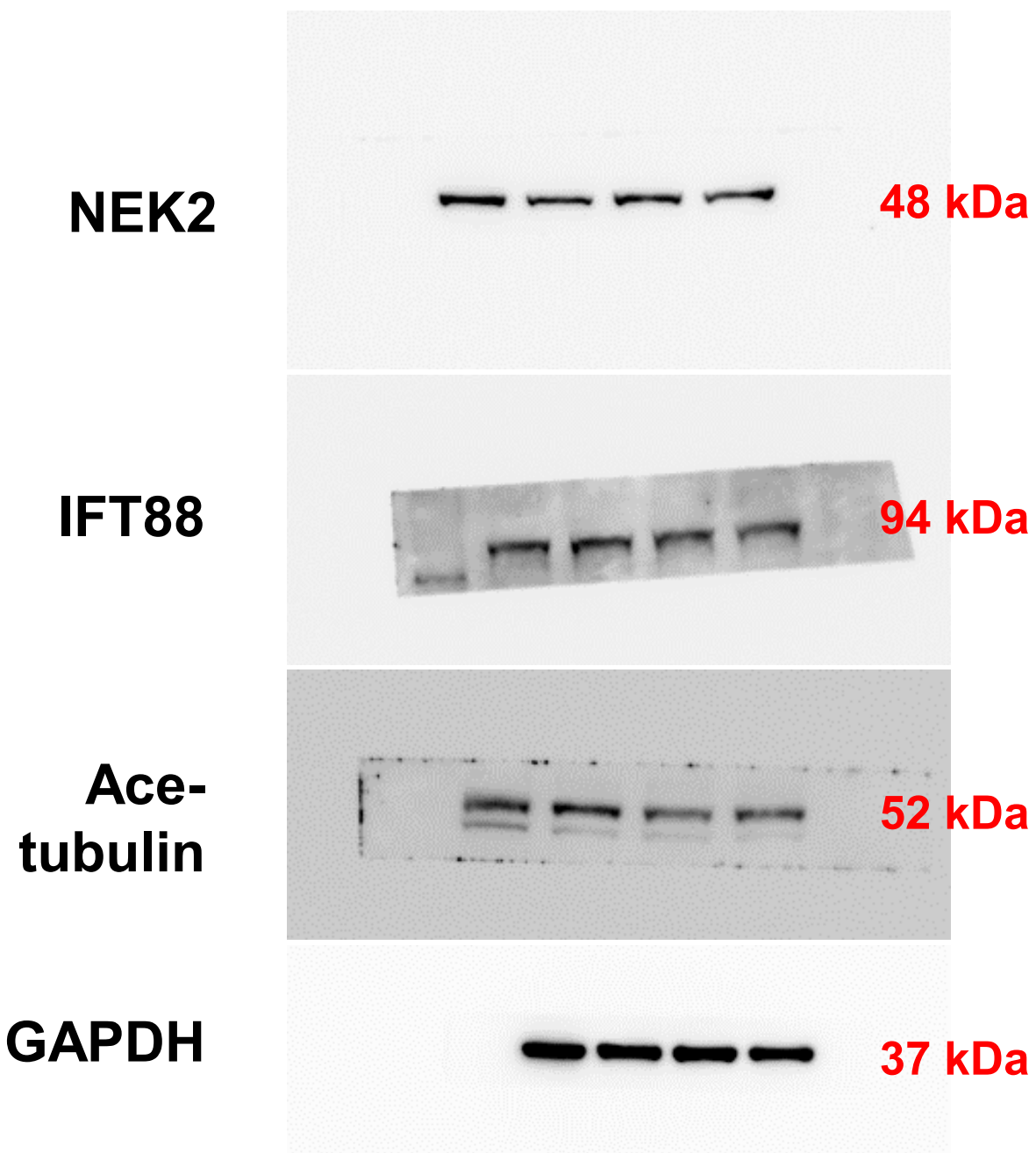

Figure 4D

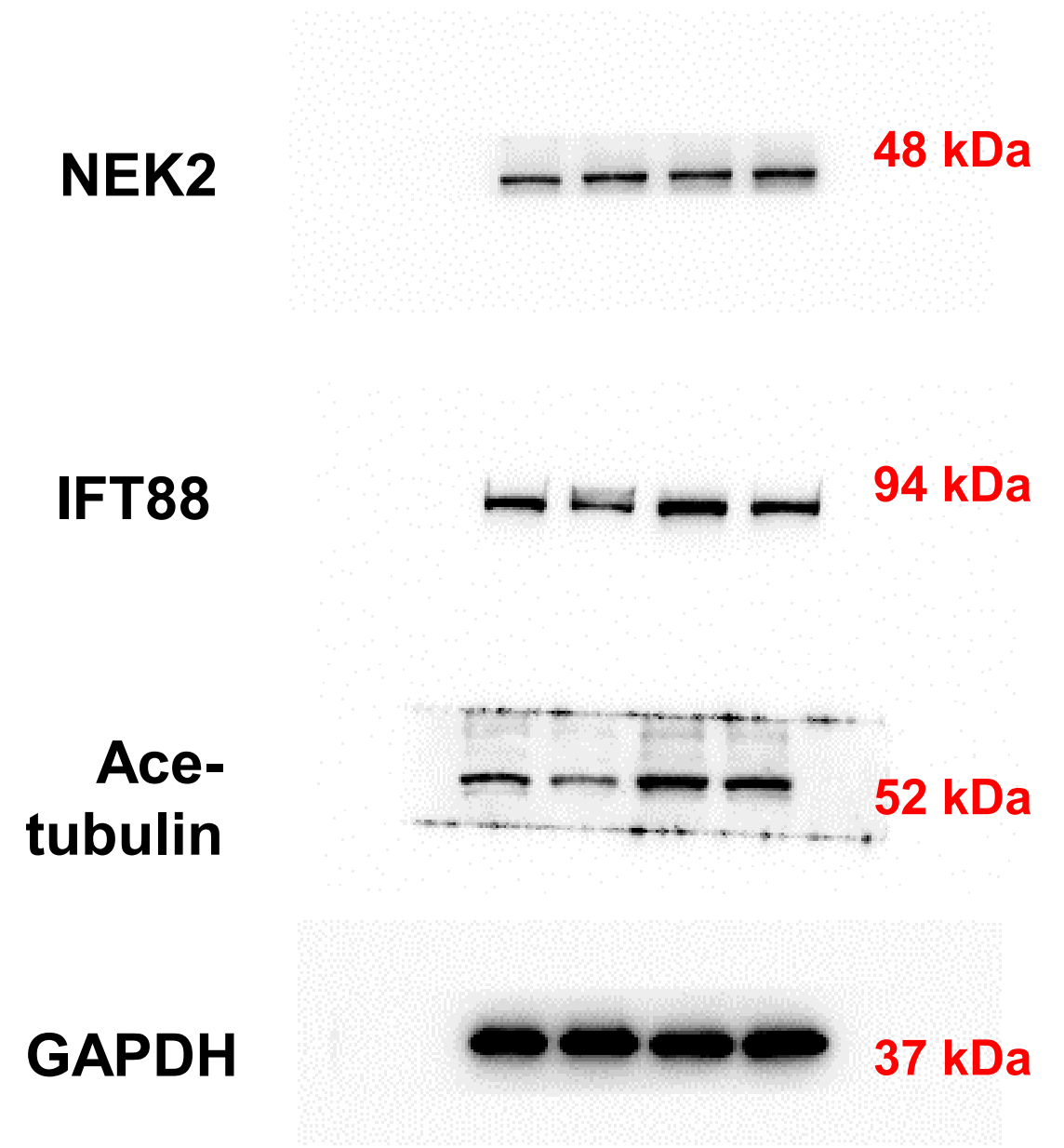

Figure 4J

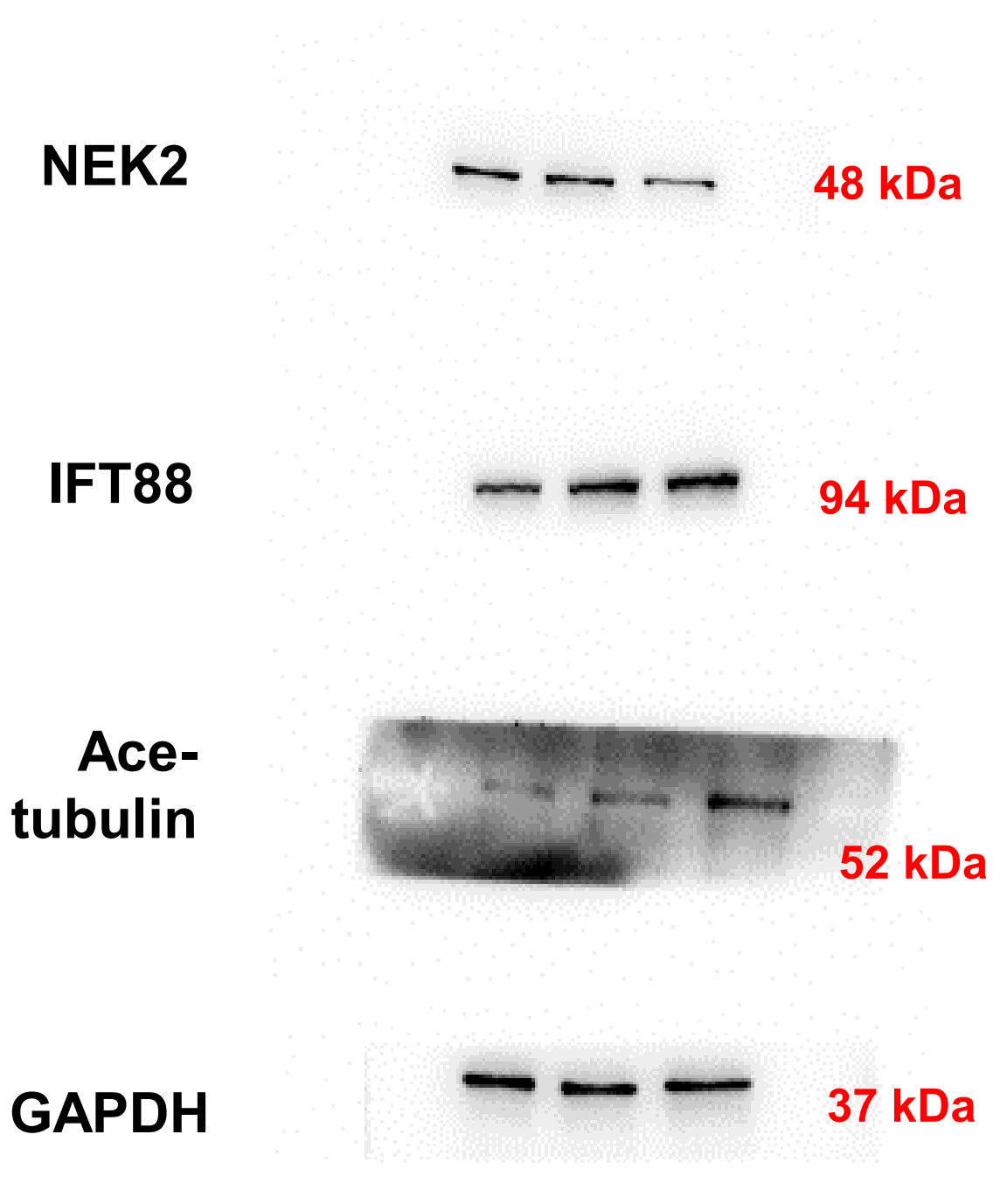

Figure 4K

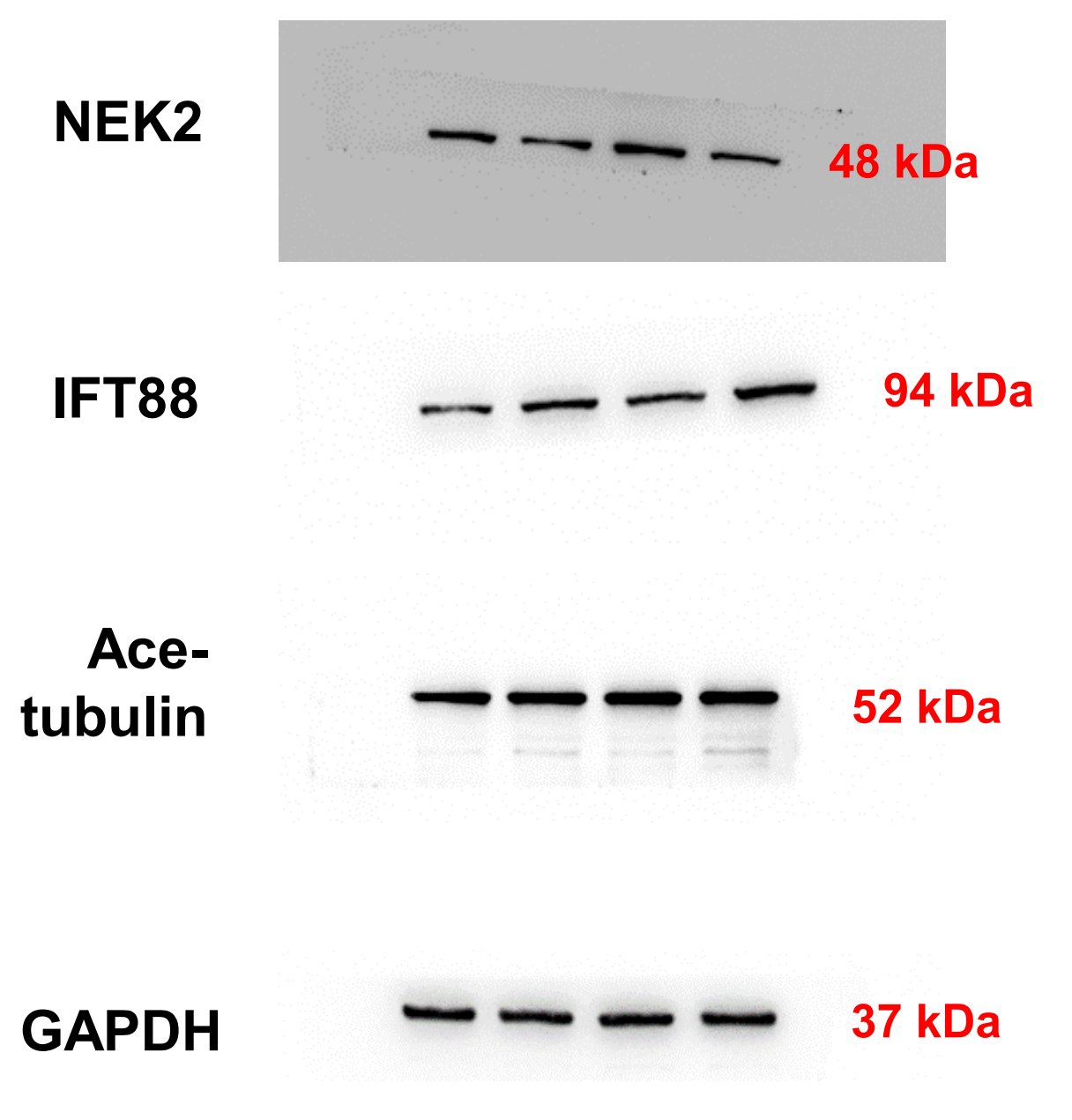

Figure 5A

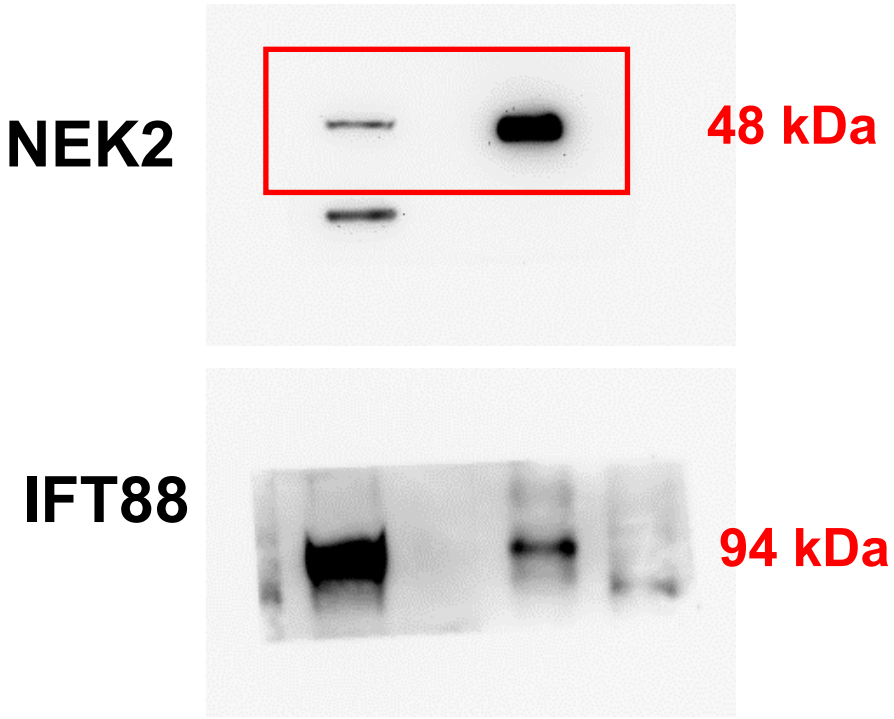

Figure 5B

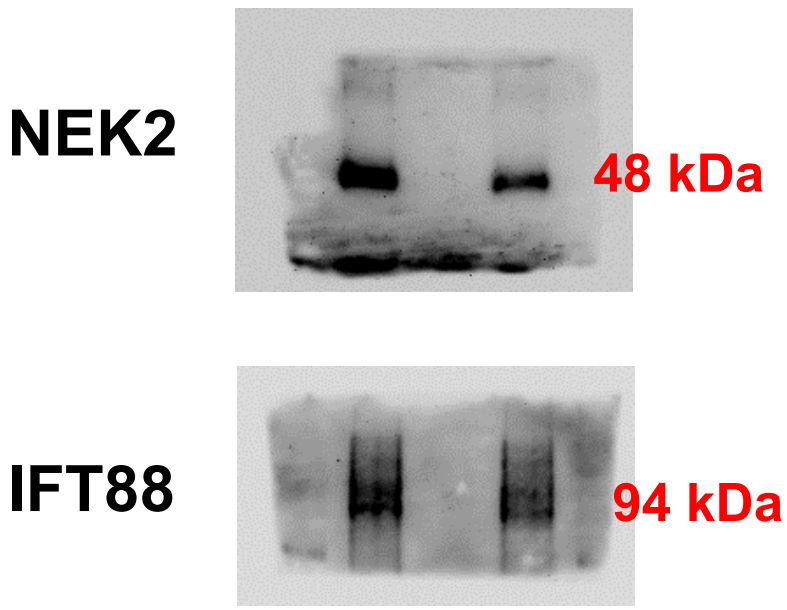

Figure 5C

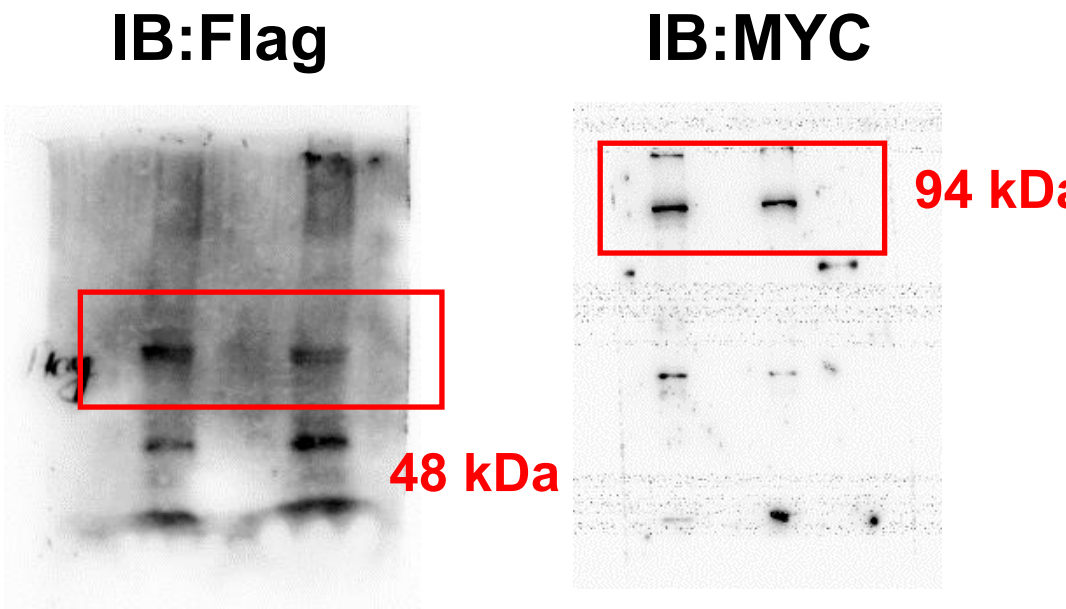

Figure 5D

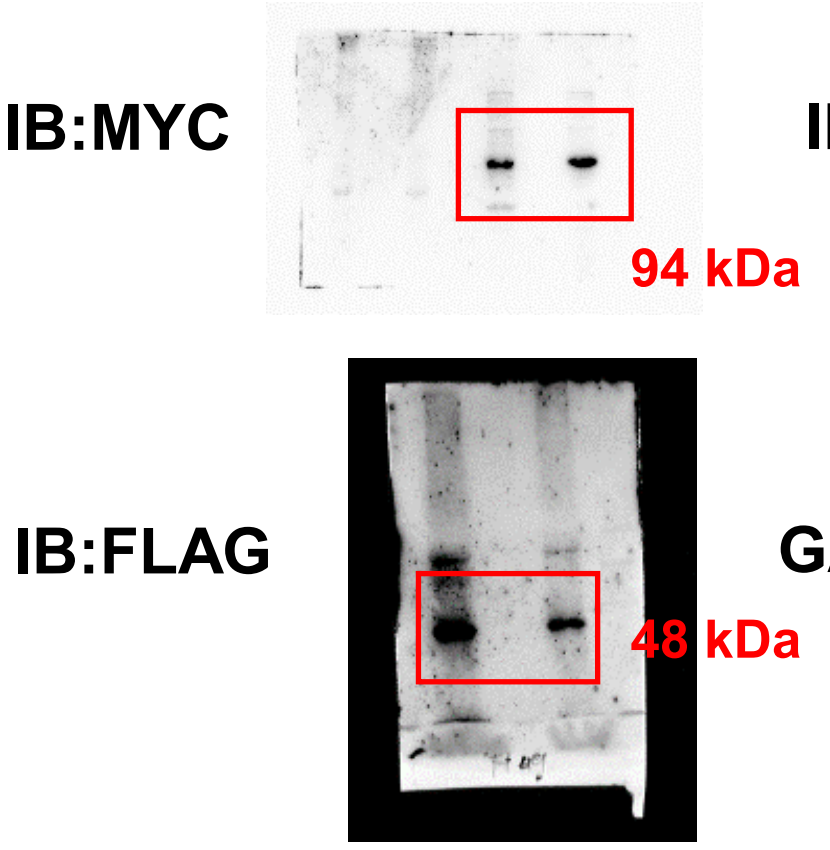

Figure 5E

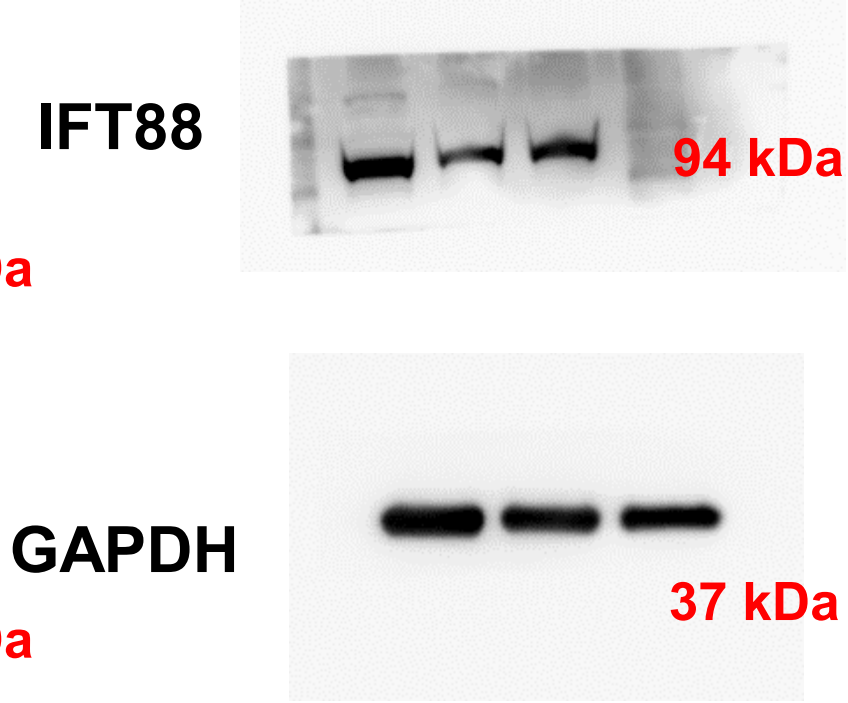

Figure 5F

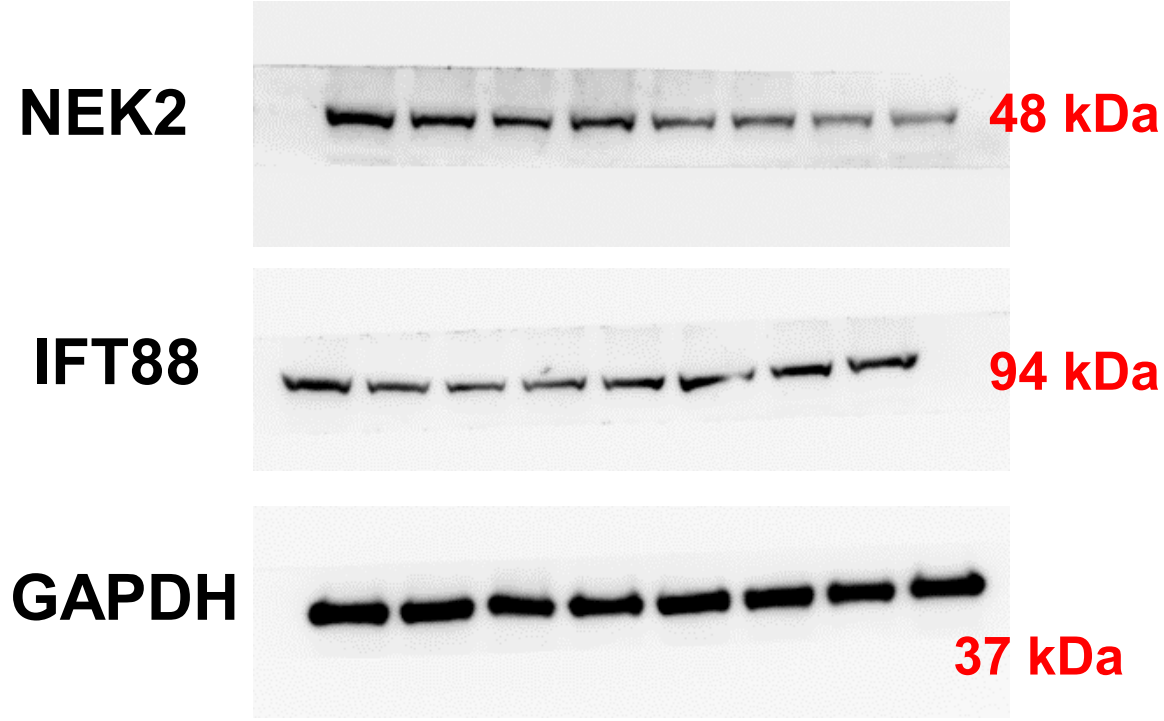

Figure 5H

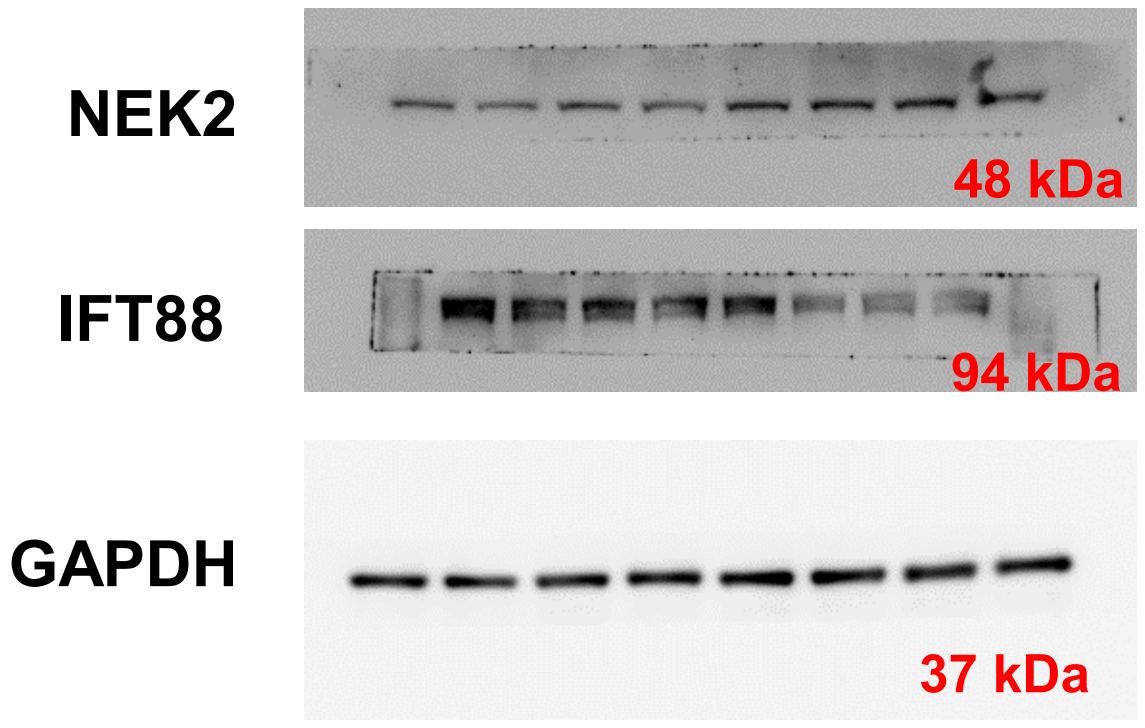

Figure 5J

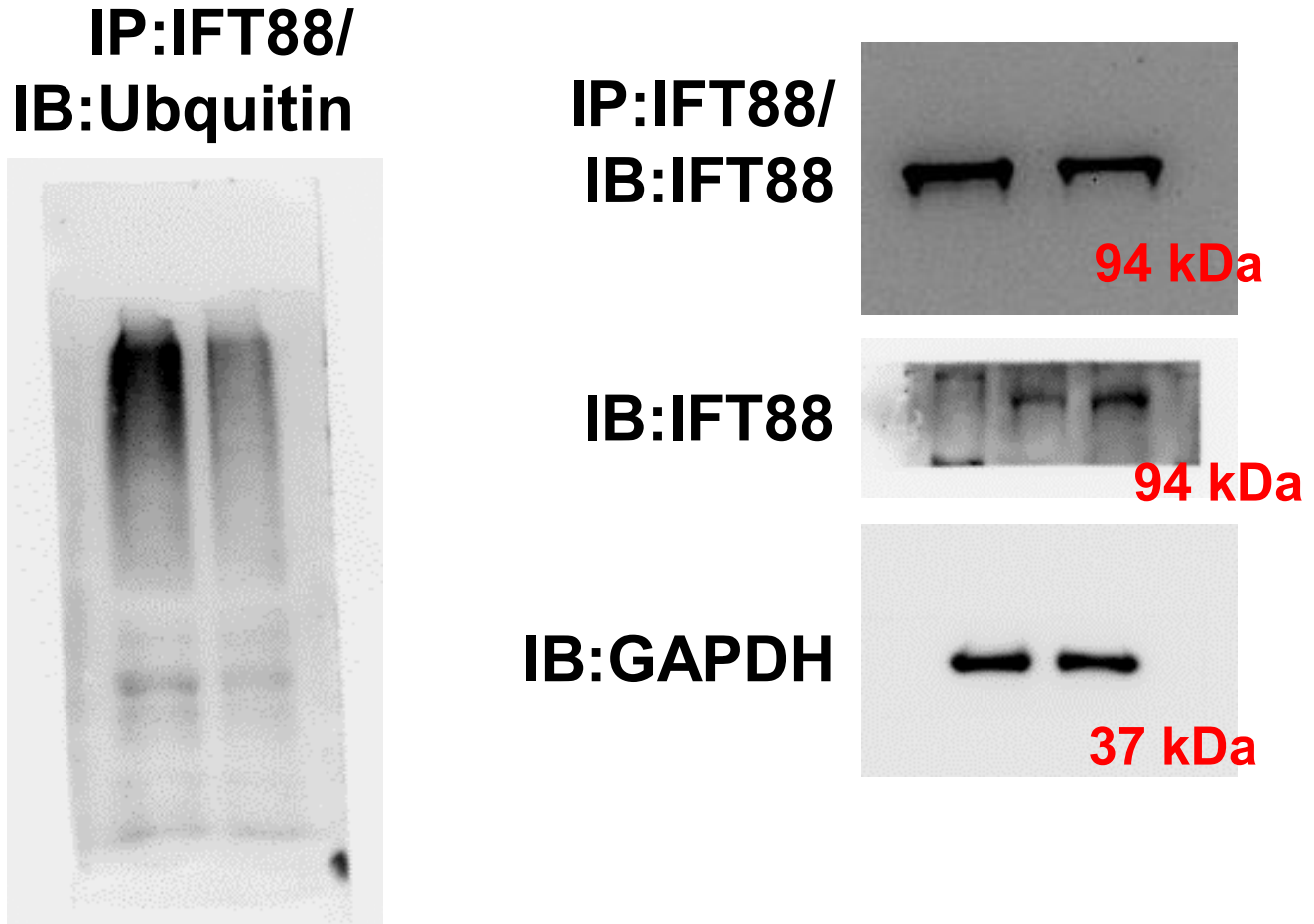

Figure 5K

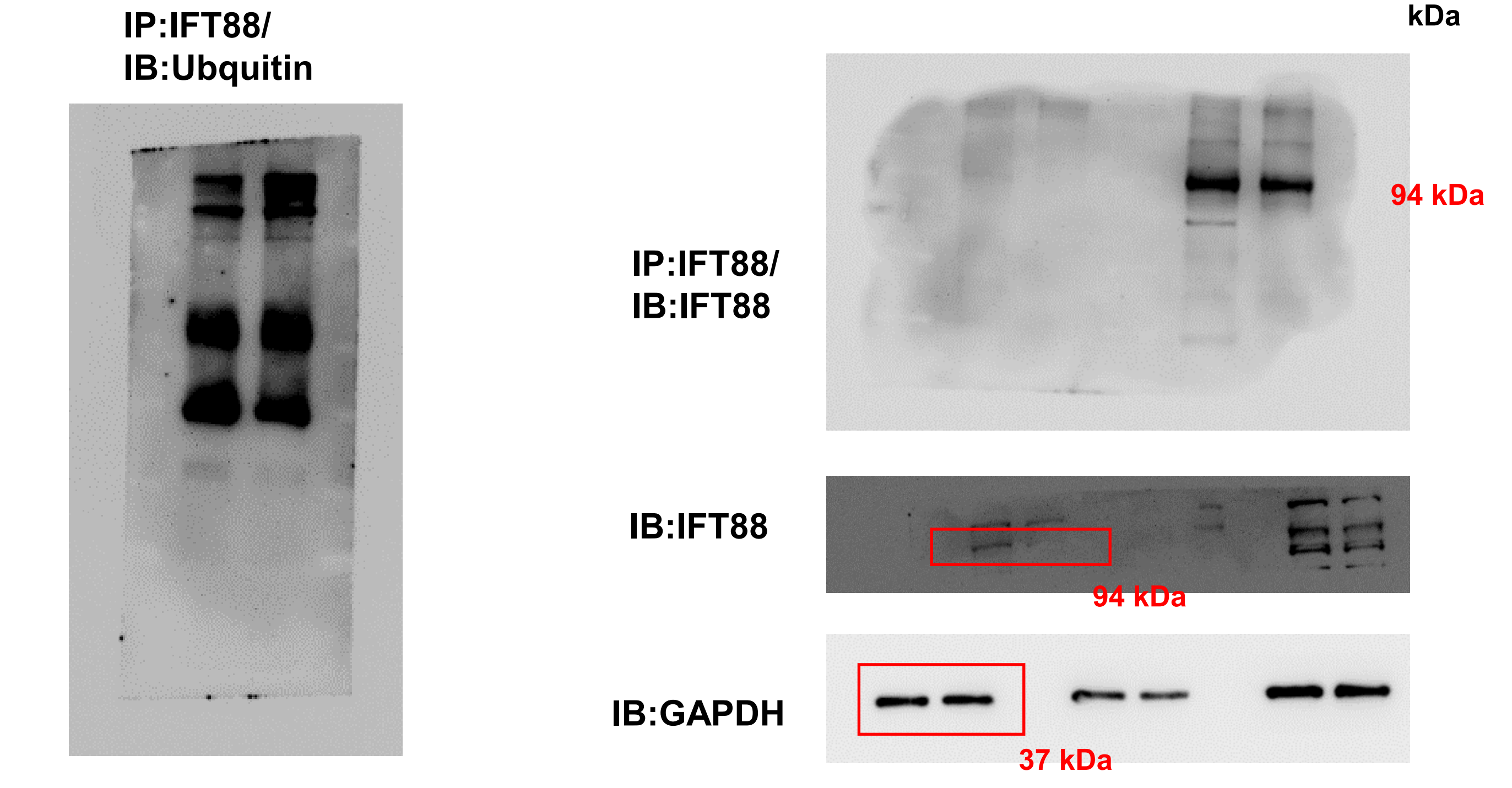

Figure 5L

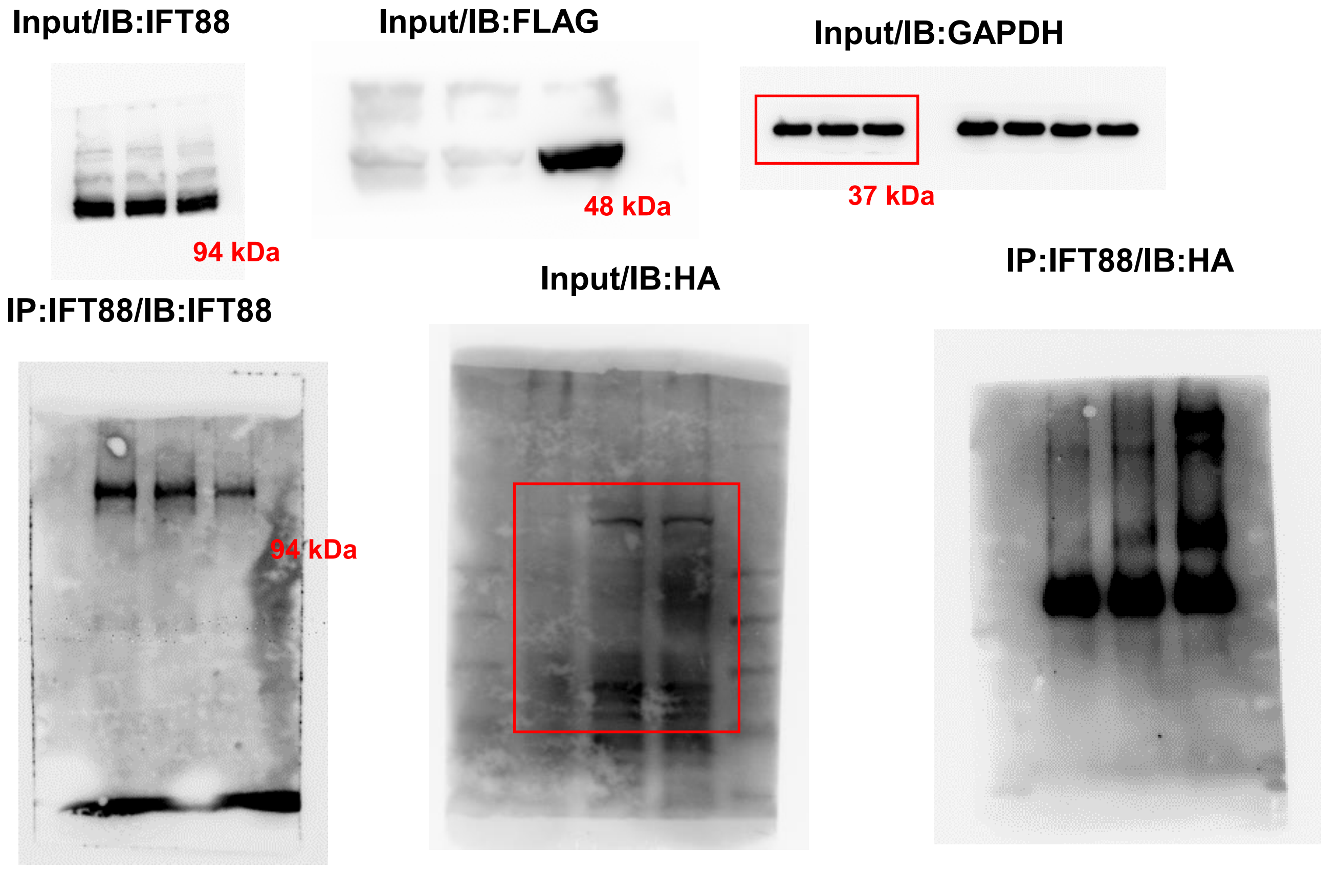

Figure 6F

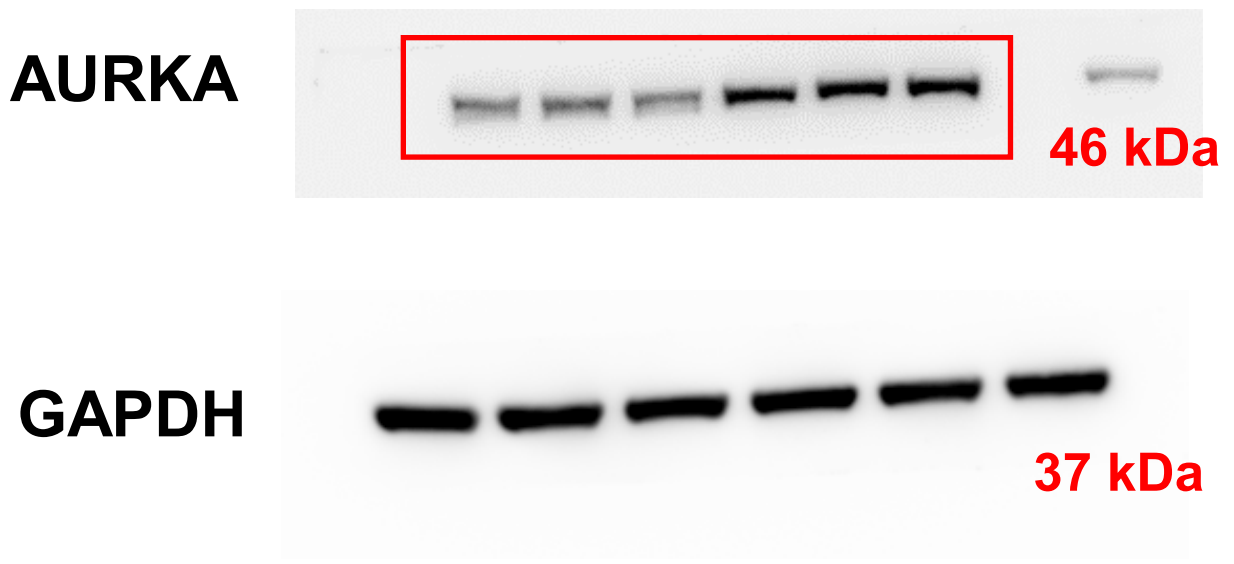

Figure 6G

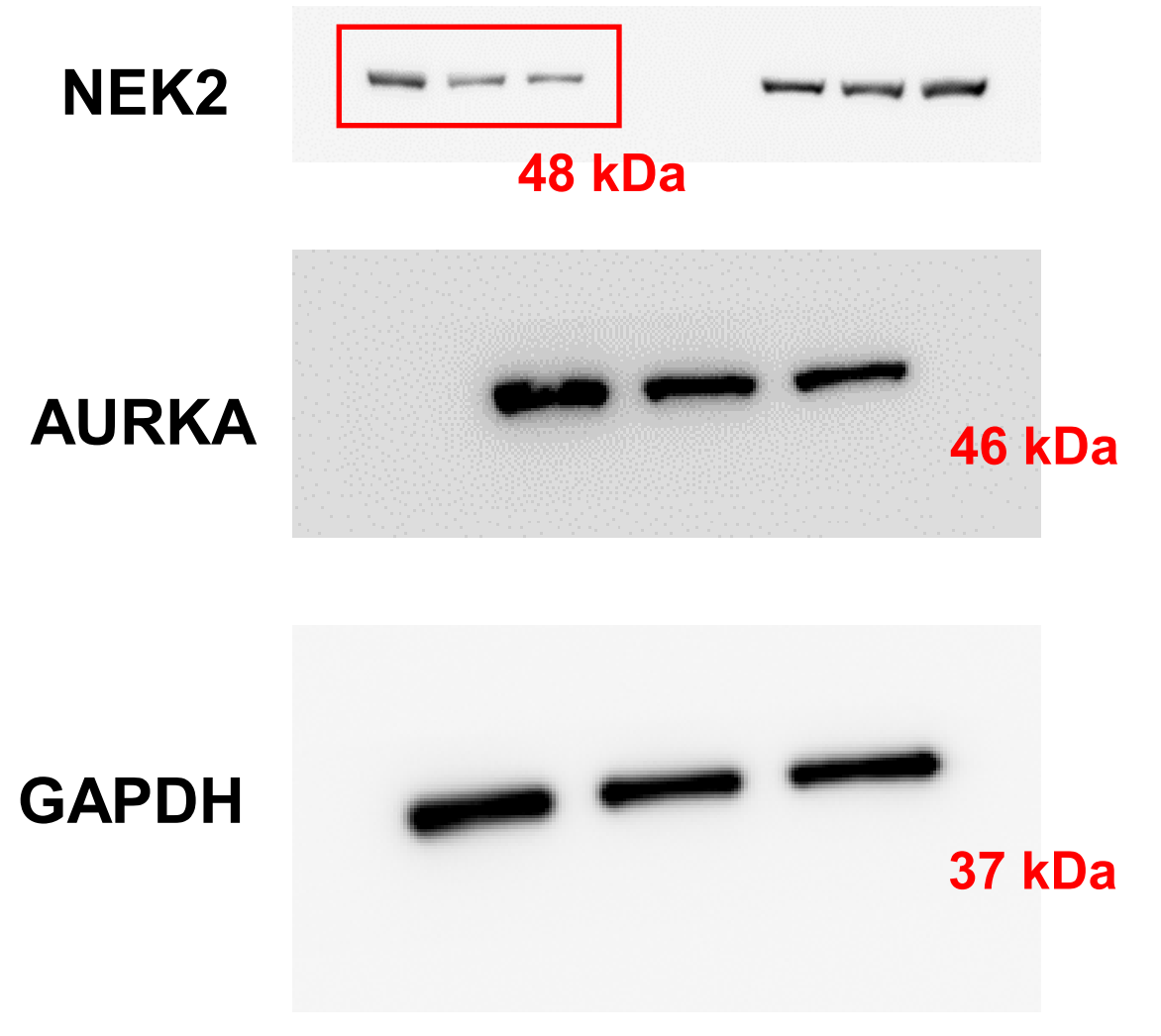

Figure 6H

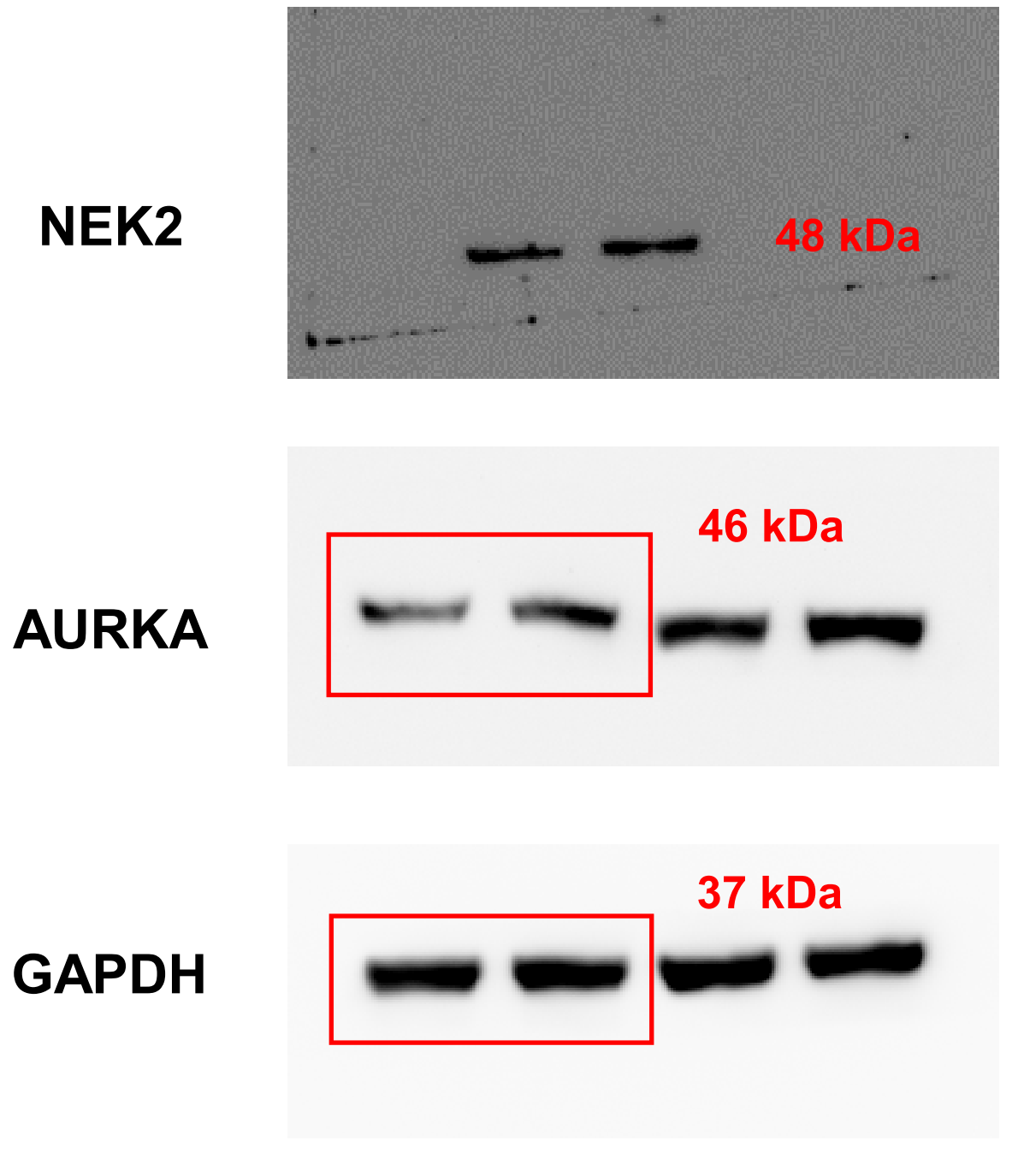

Figure 6I

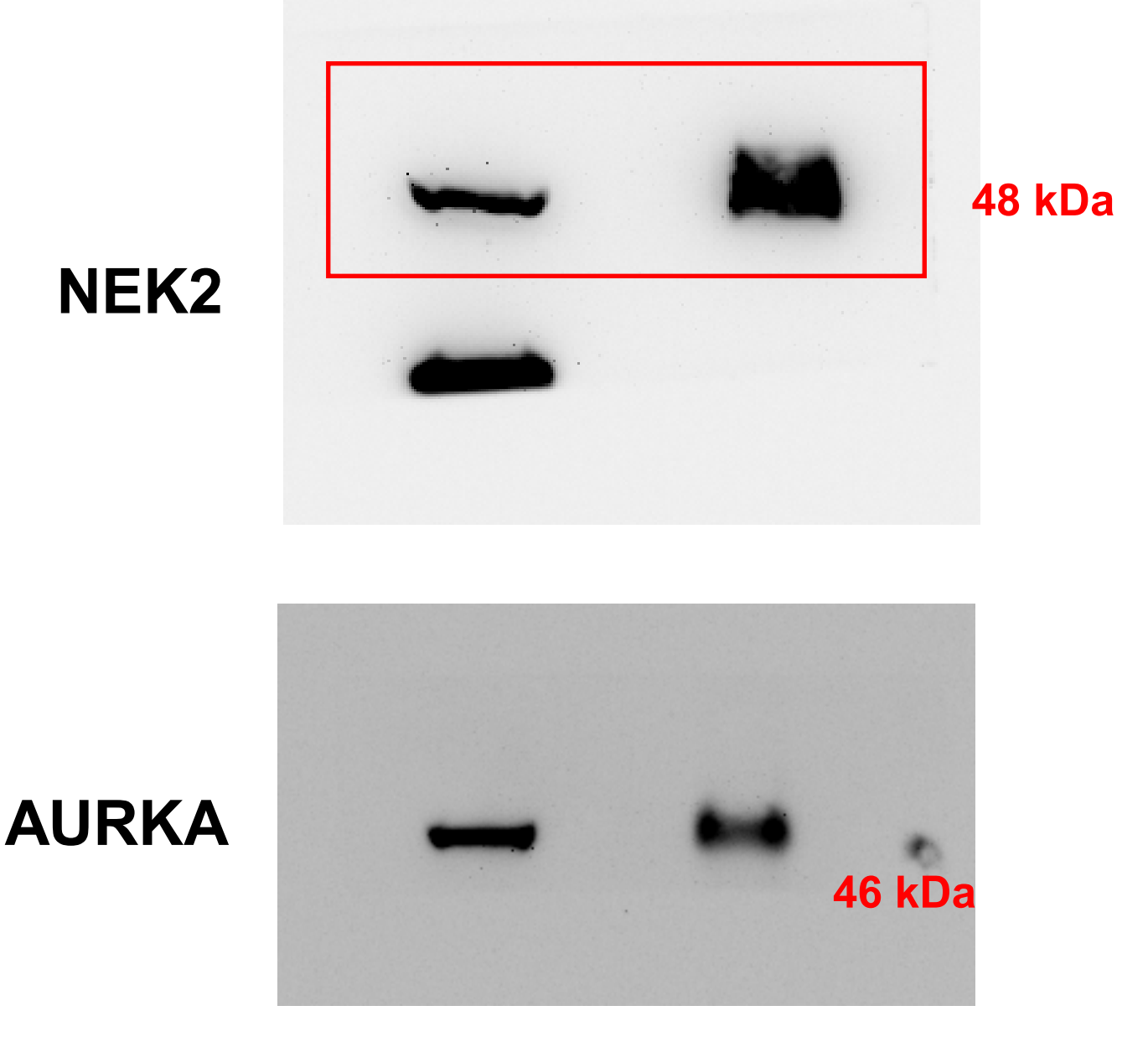

Figure 6J

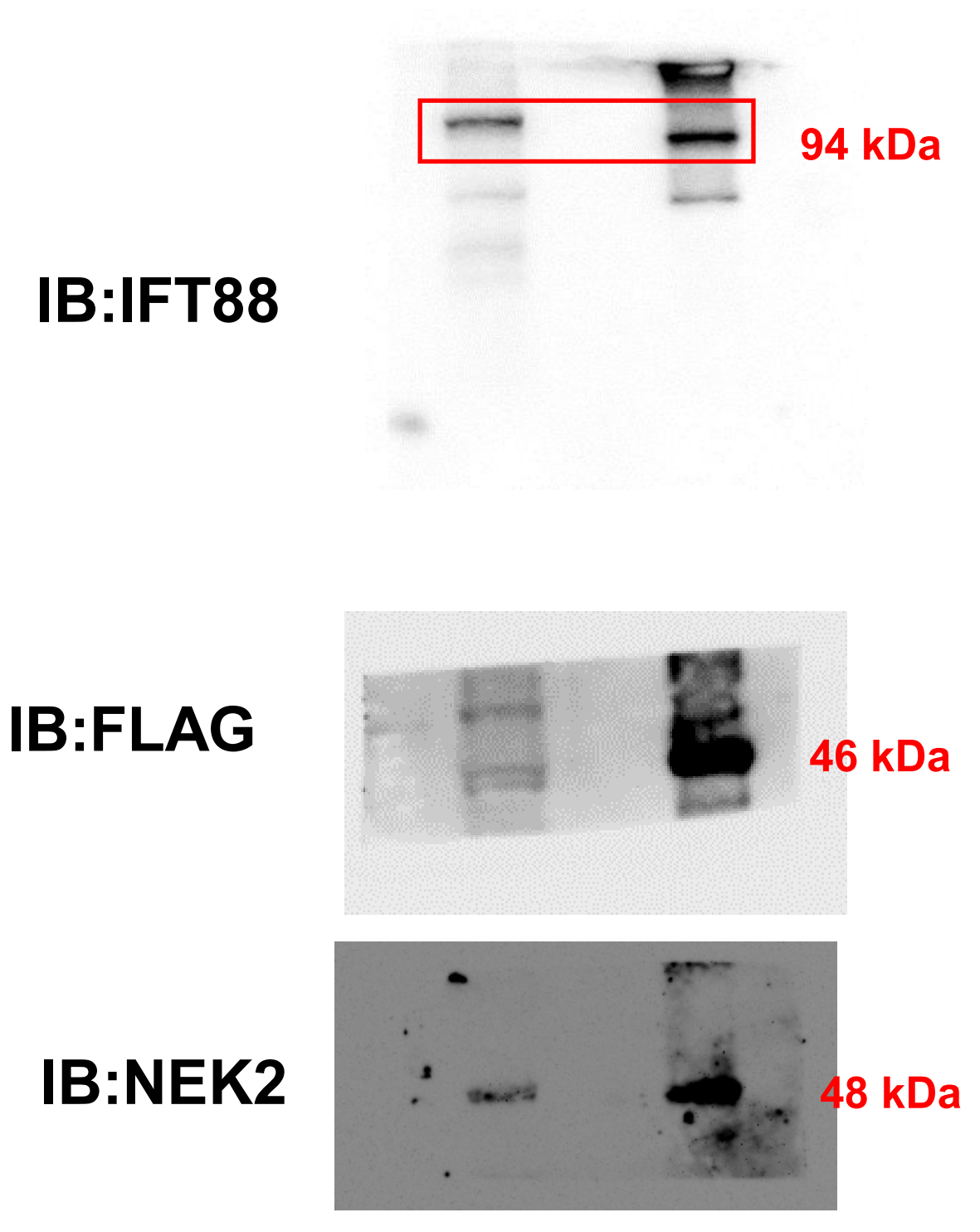

Figure 6K

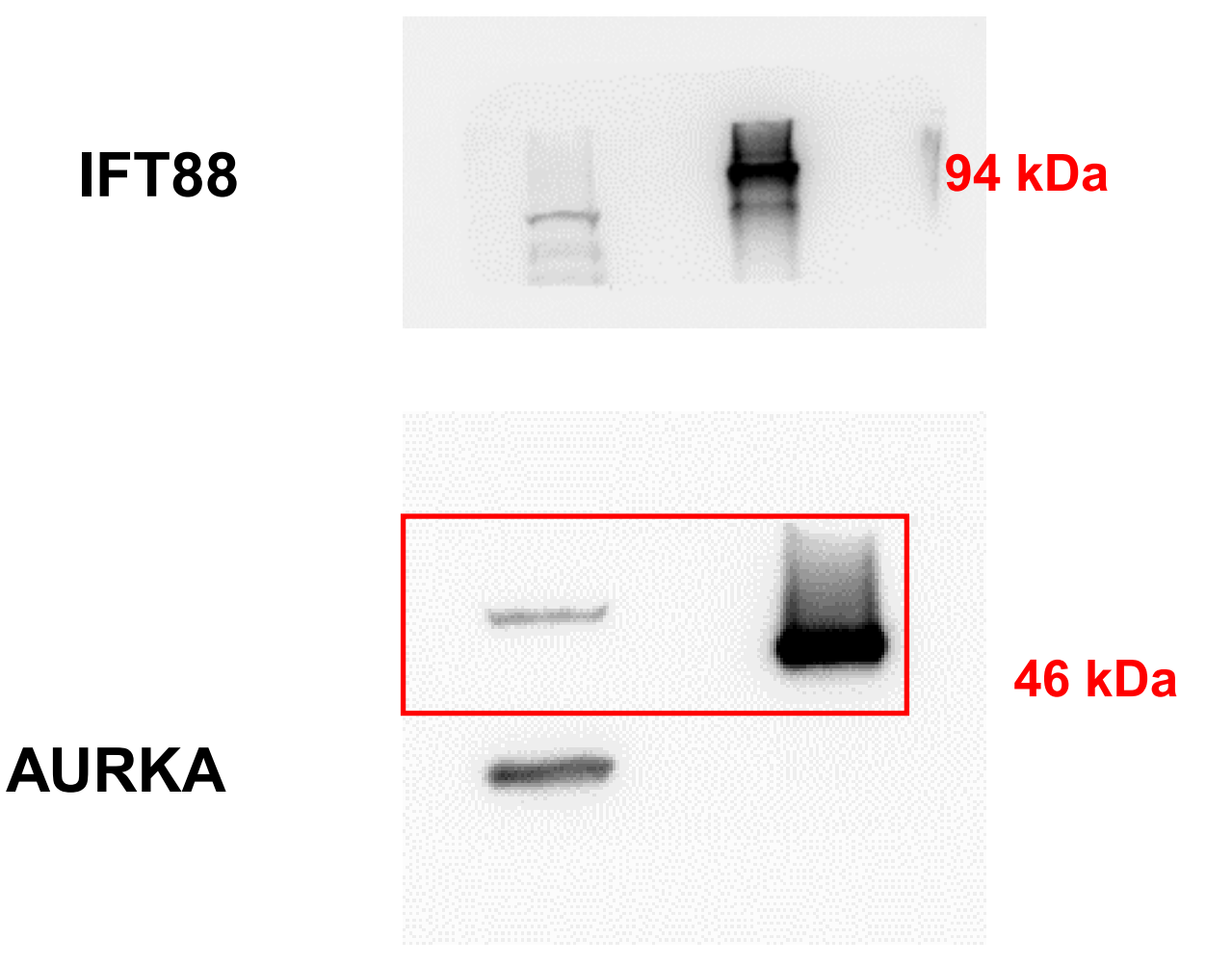

Figure 6M

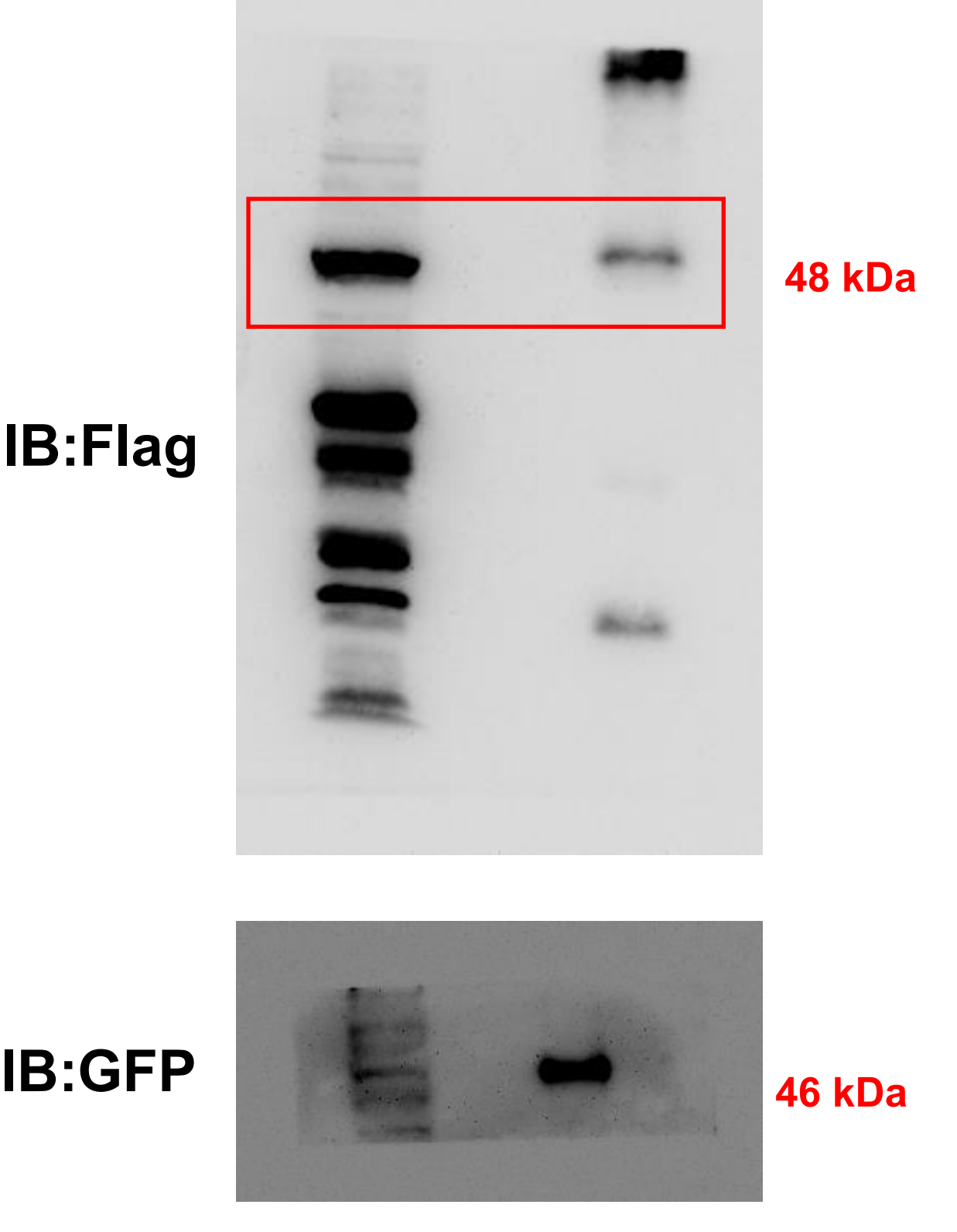

Figure 6L

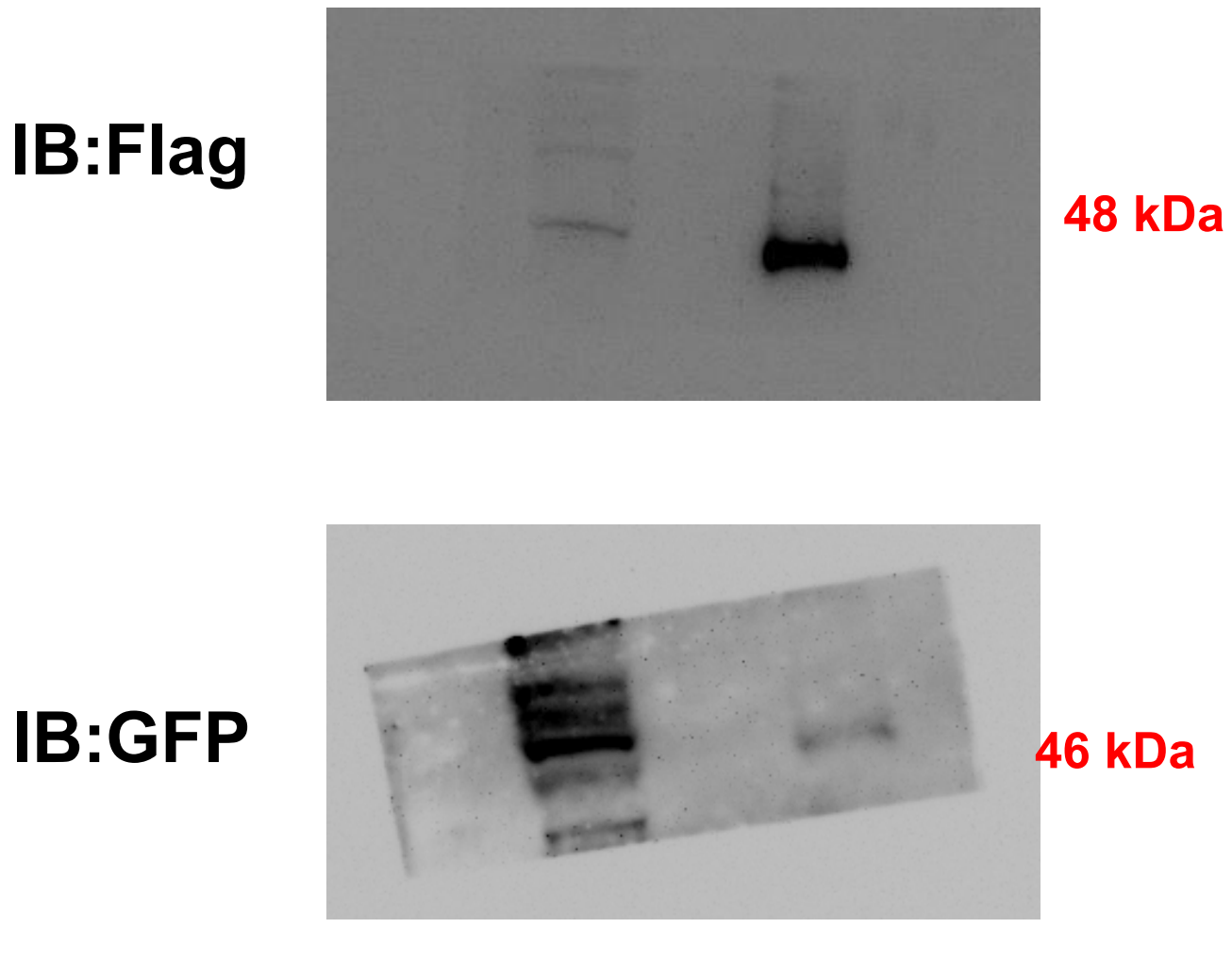

Figure 7I

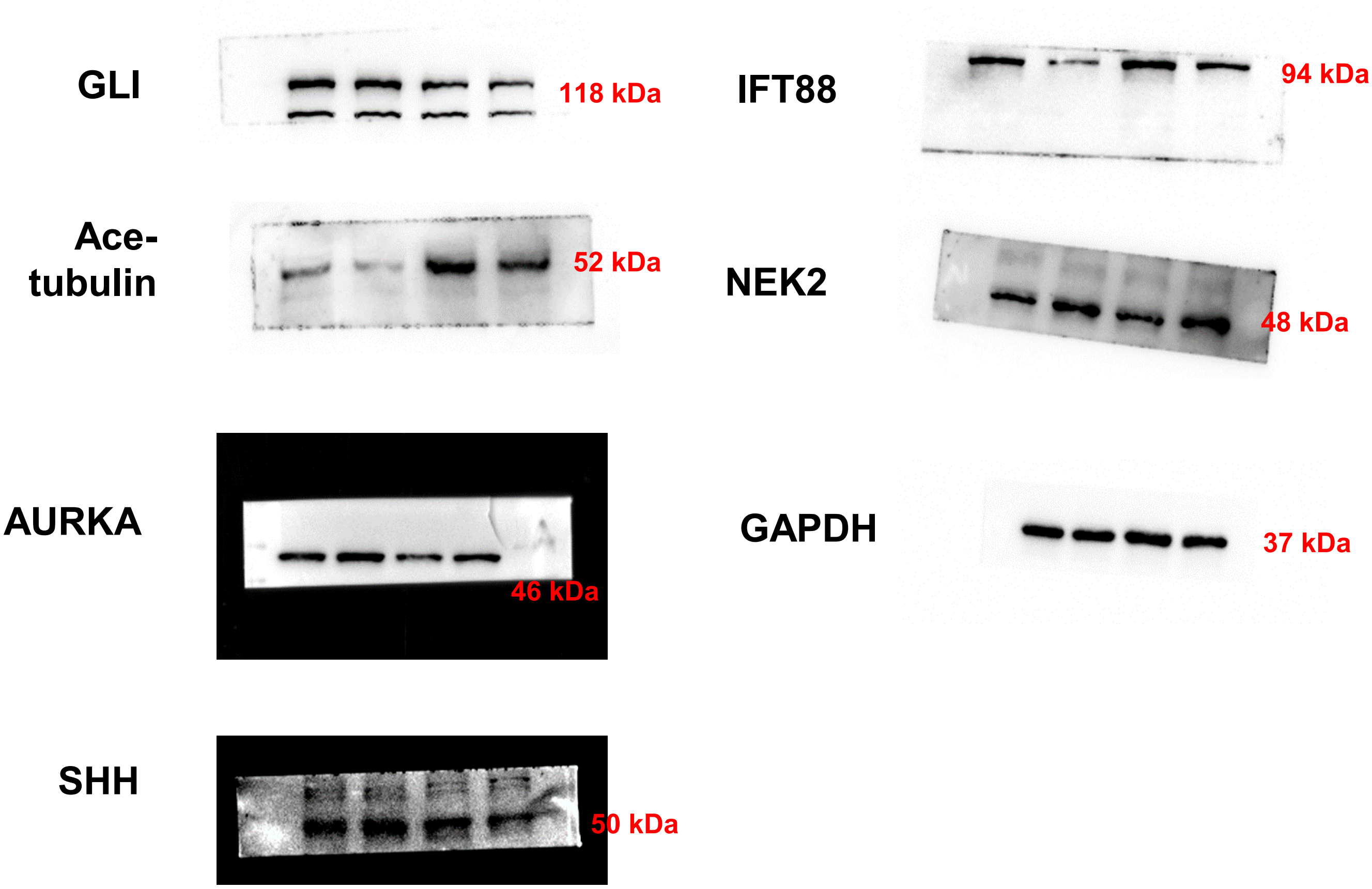

Figure 7J

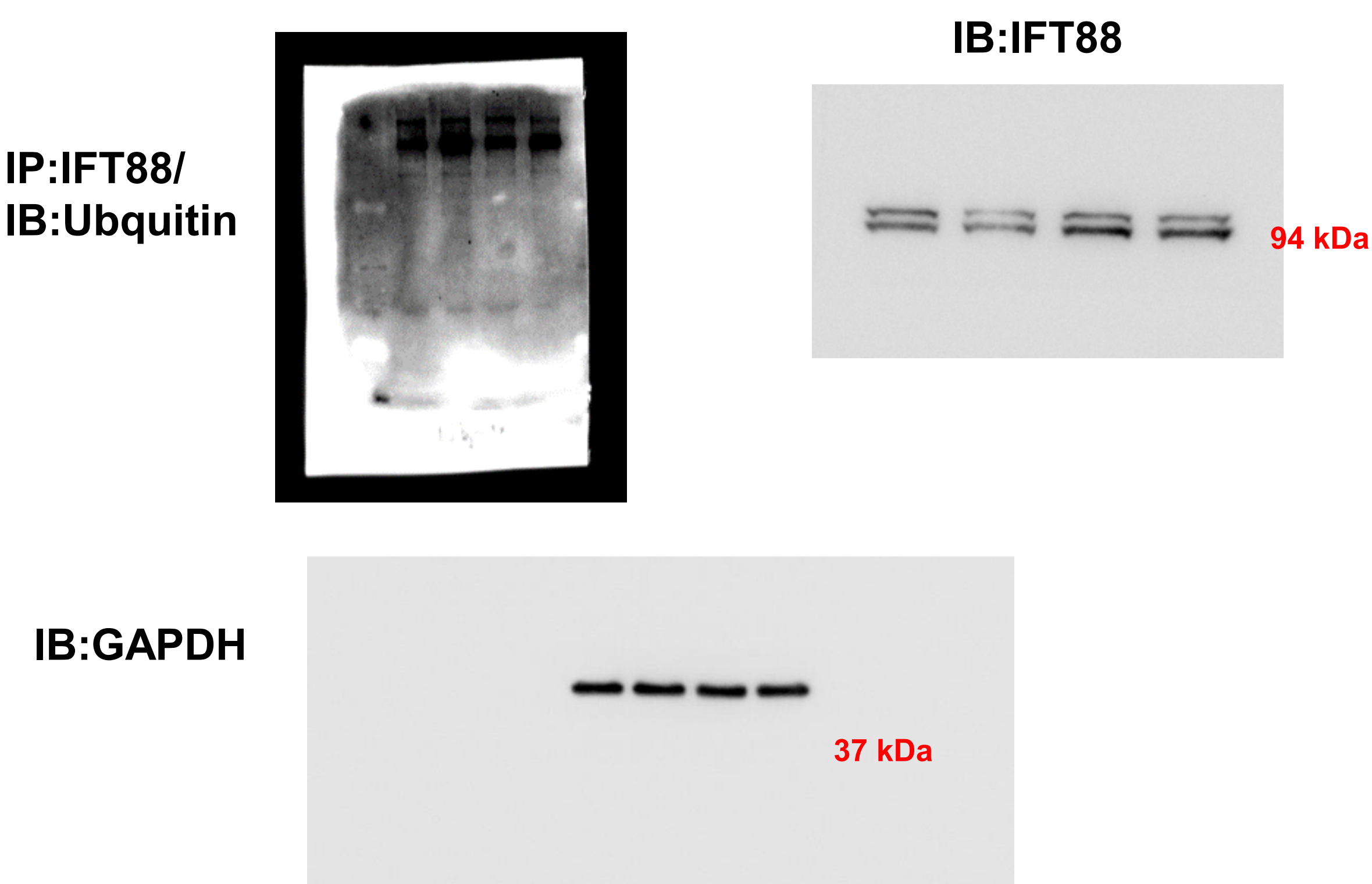

Figure S1G

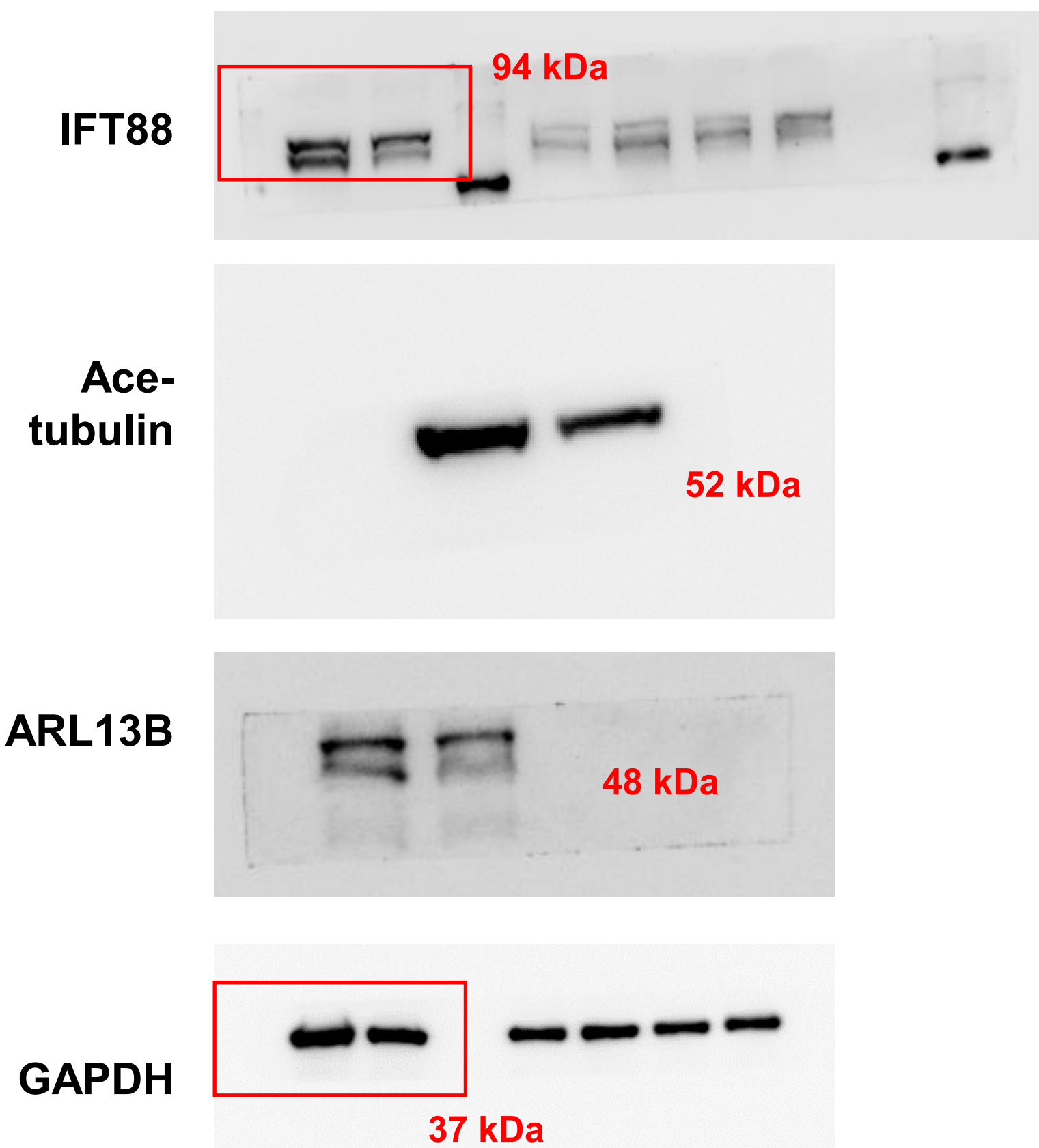

Figure S1H

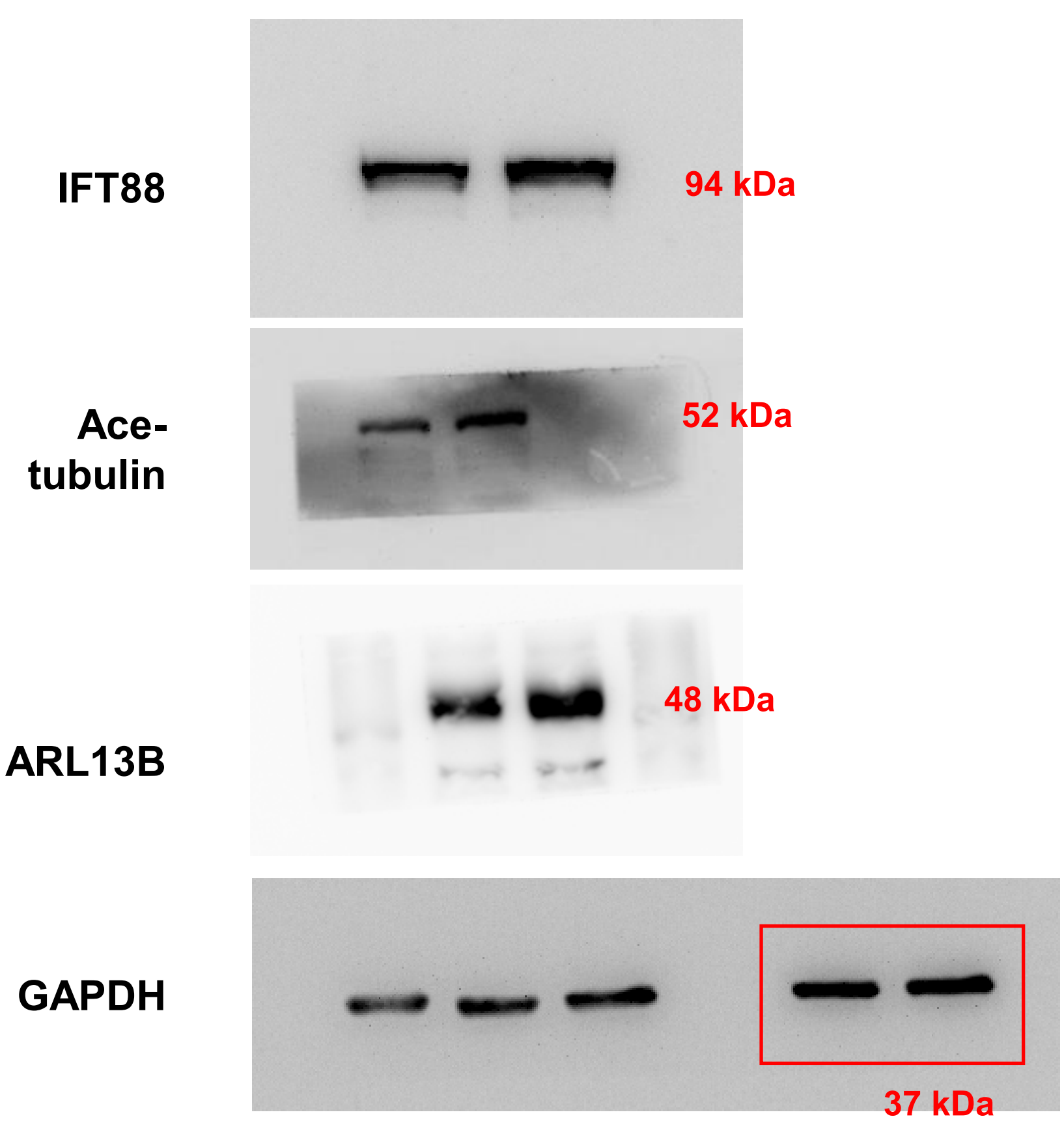

Figure S2A

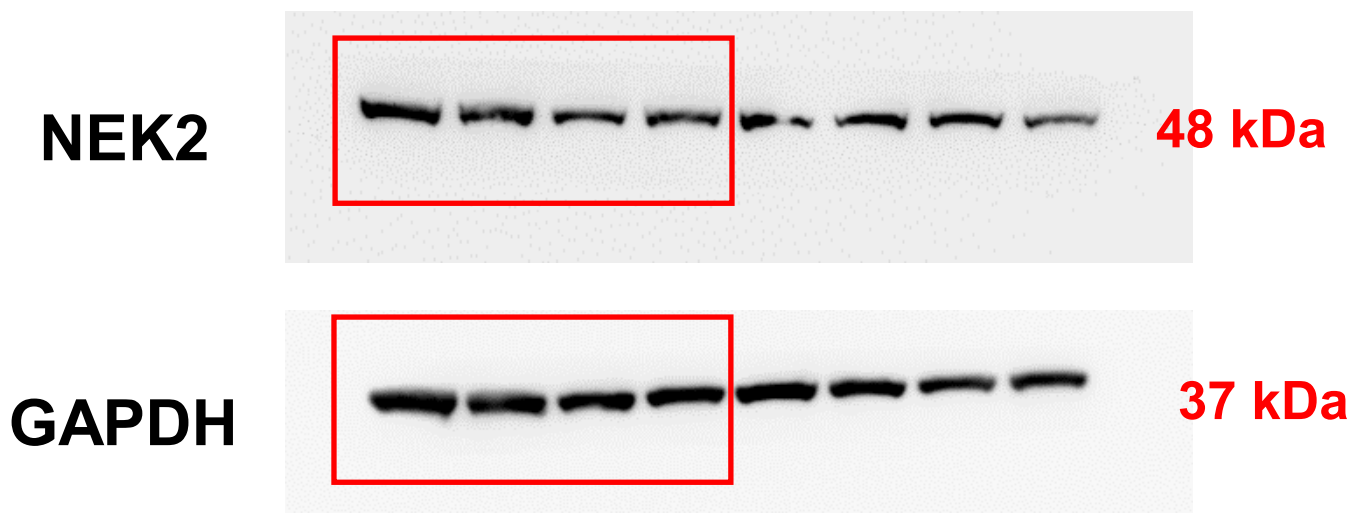

Figure S2B

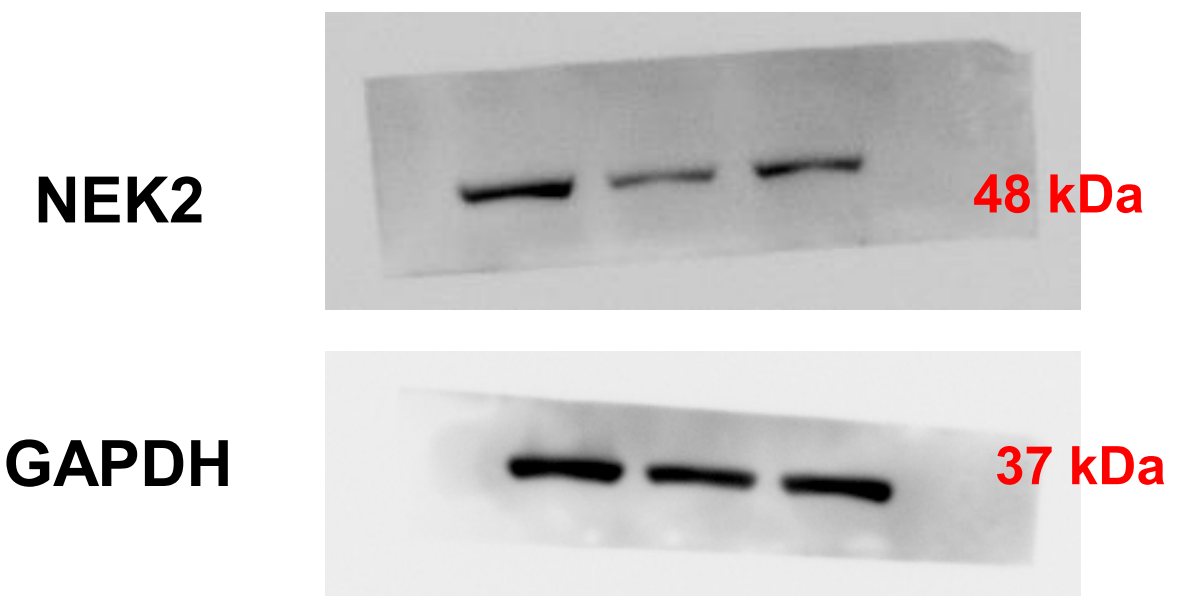

Figure S2H

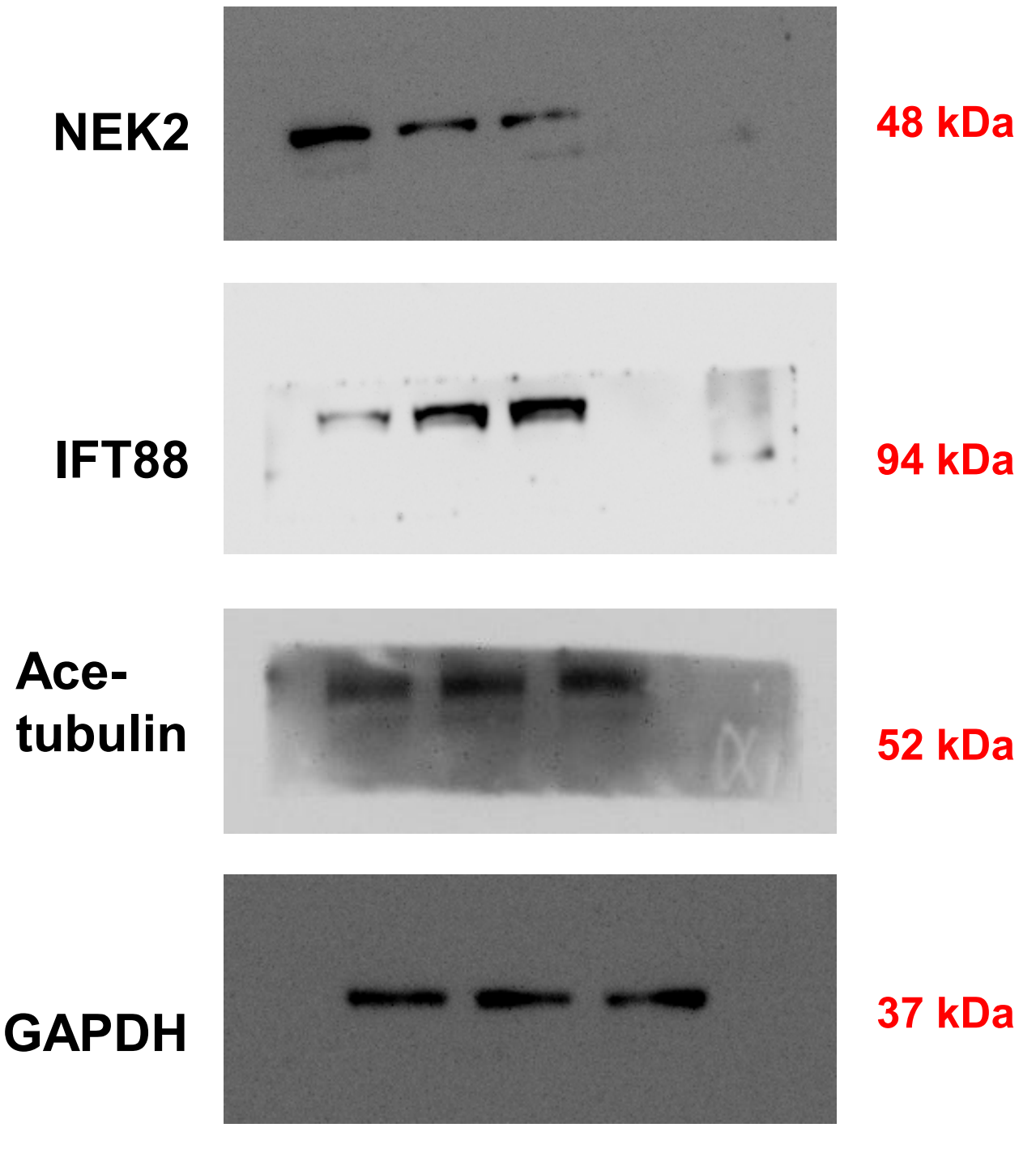

Figure S2I

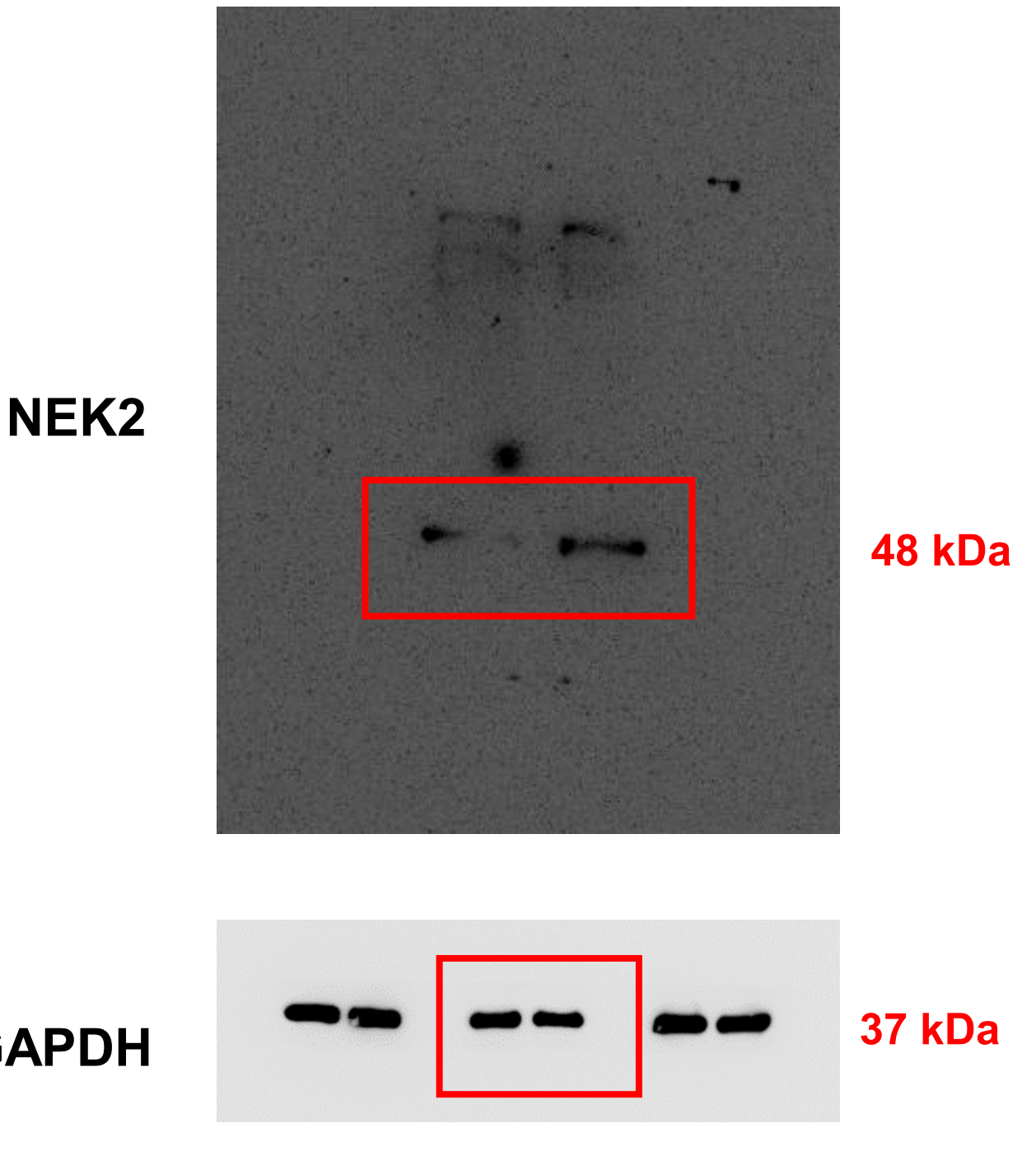

Figure S2O

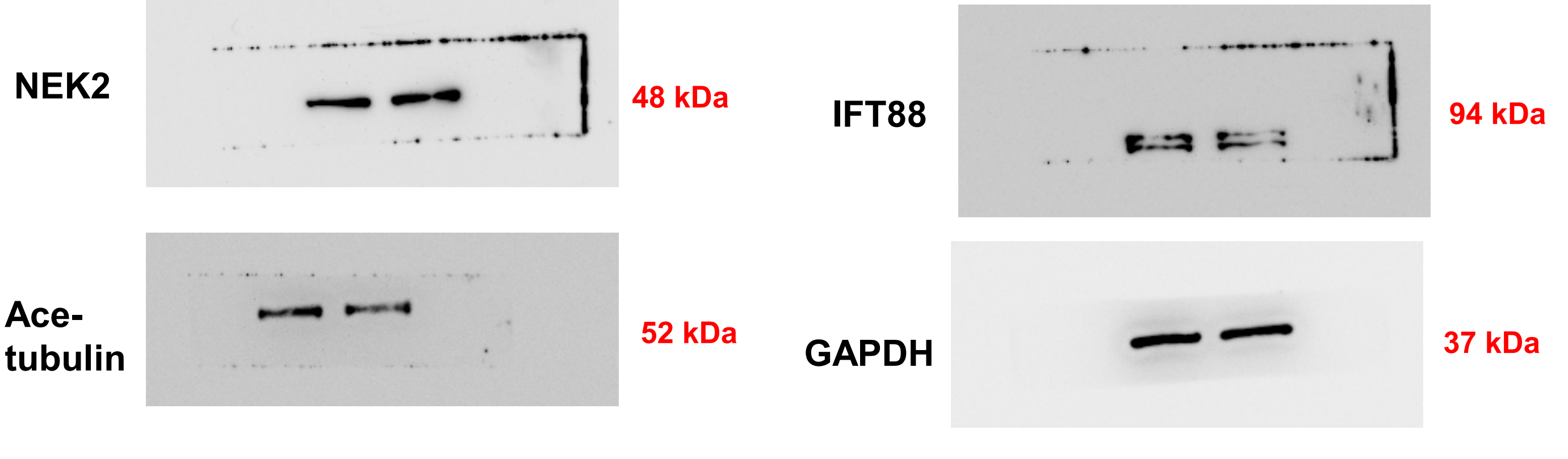

Figure S4B

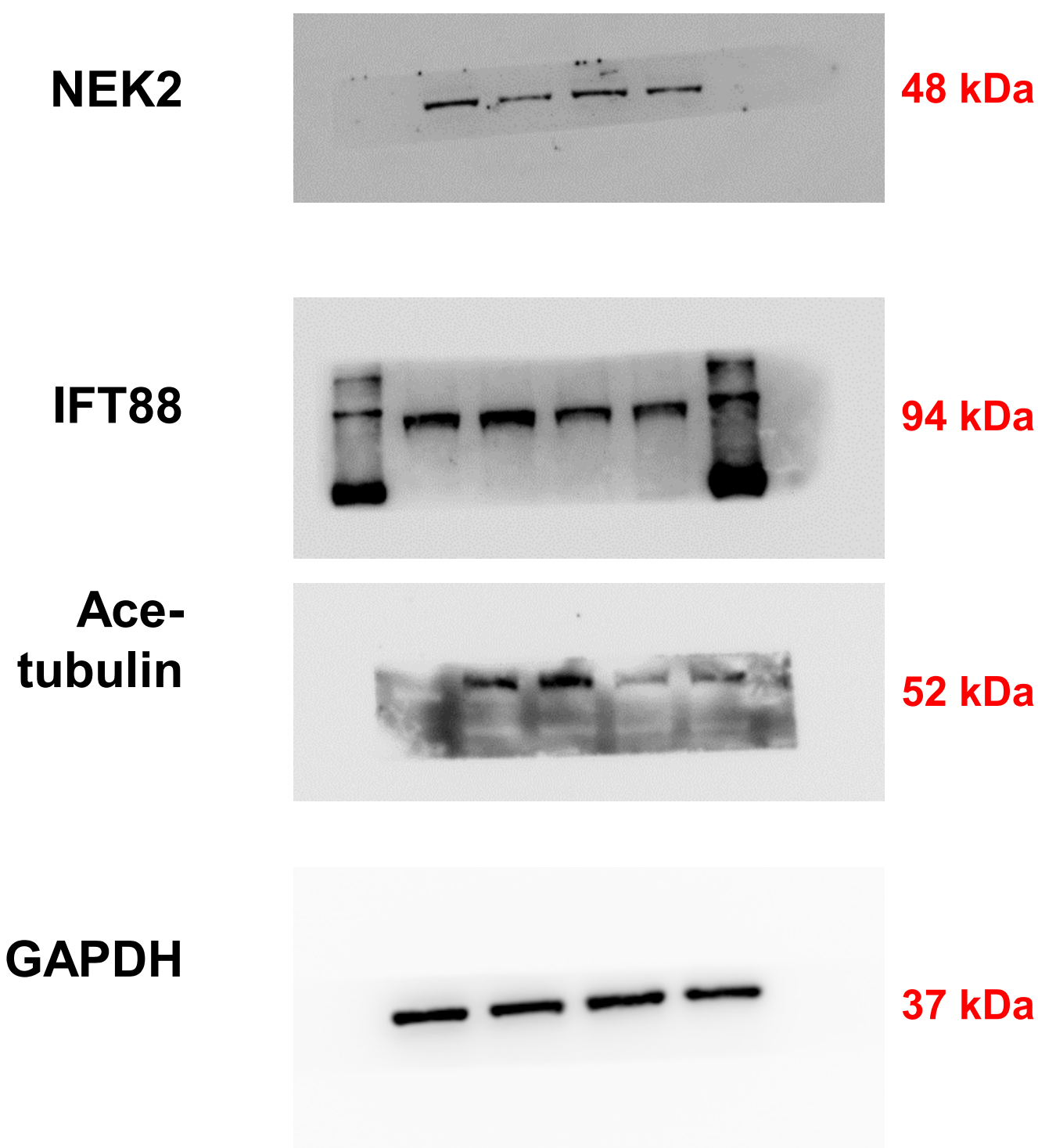

Figure S4D

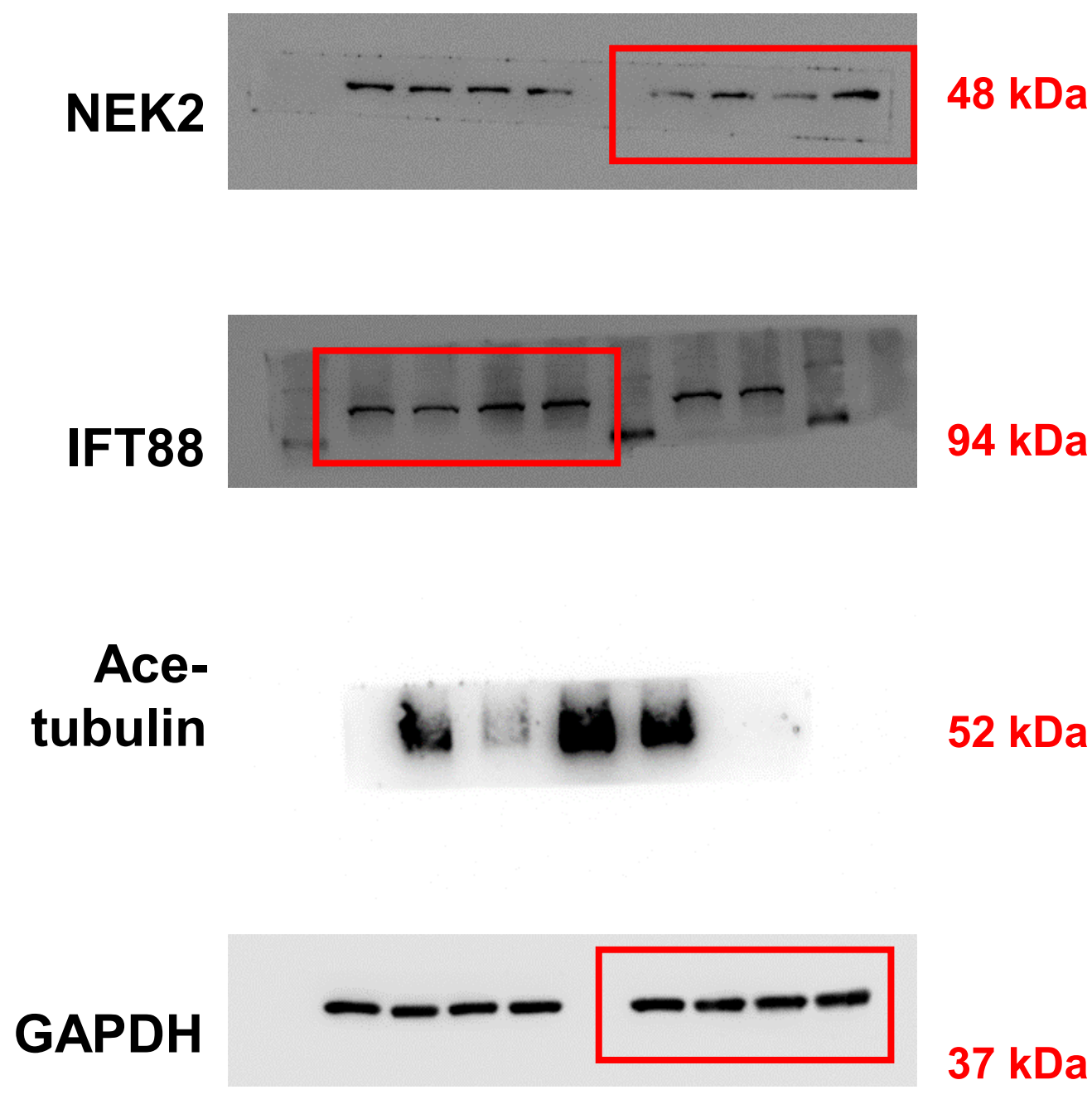

Figure S4H

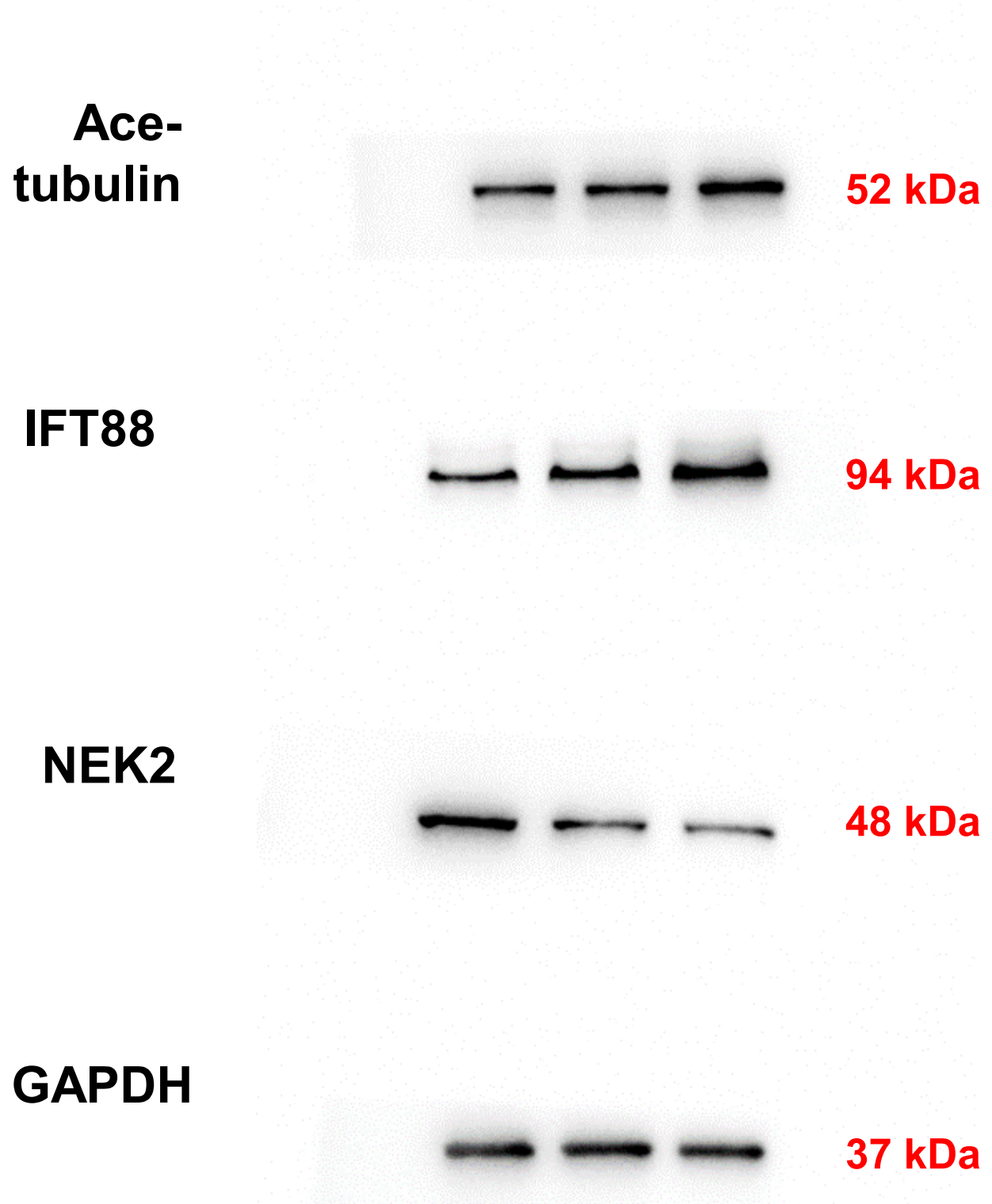

Figure S4I

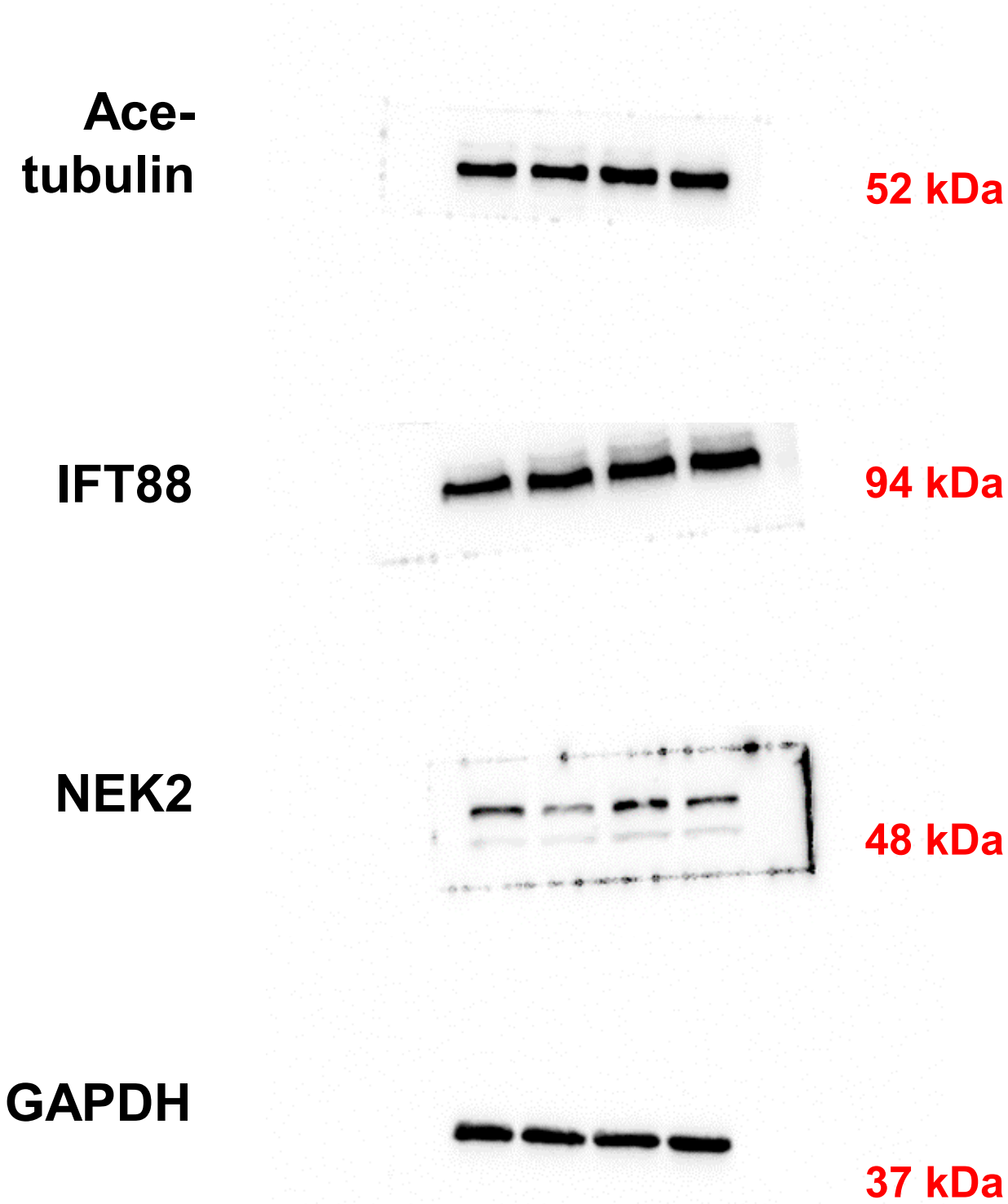

Figure S5A

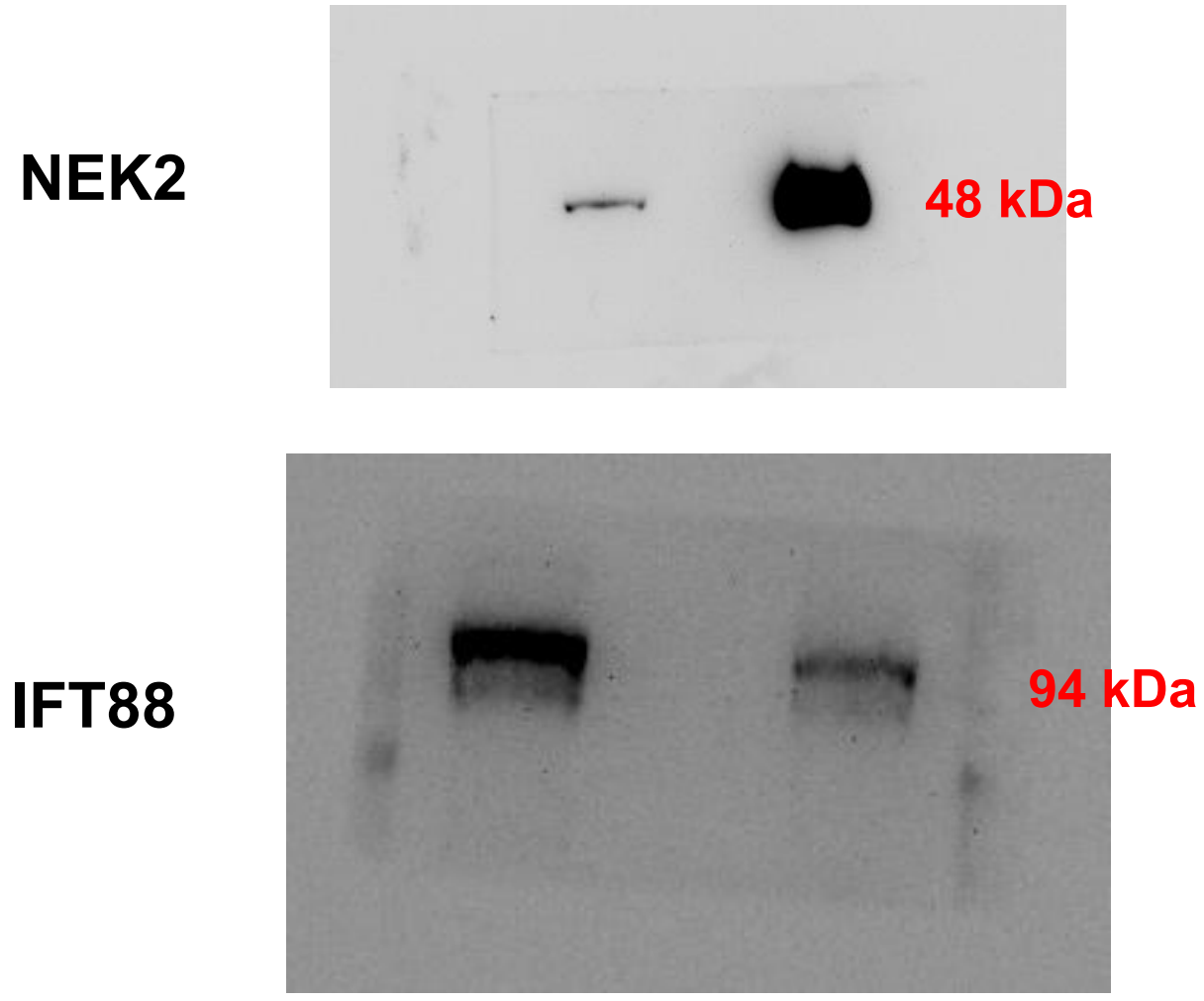

Figure S5B

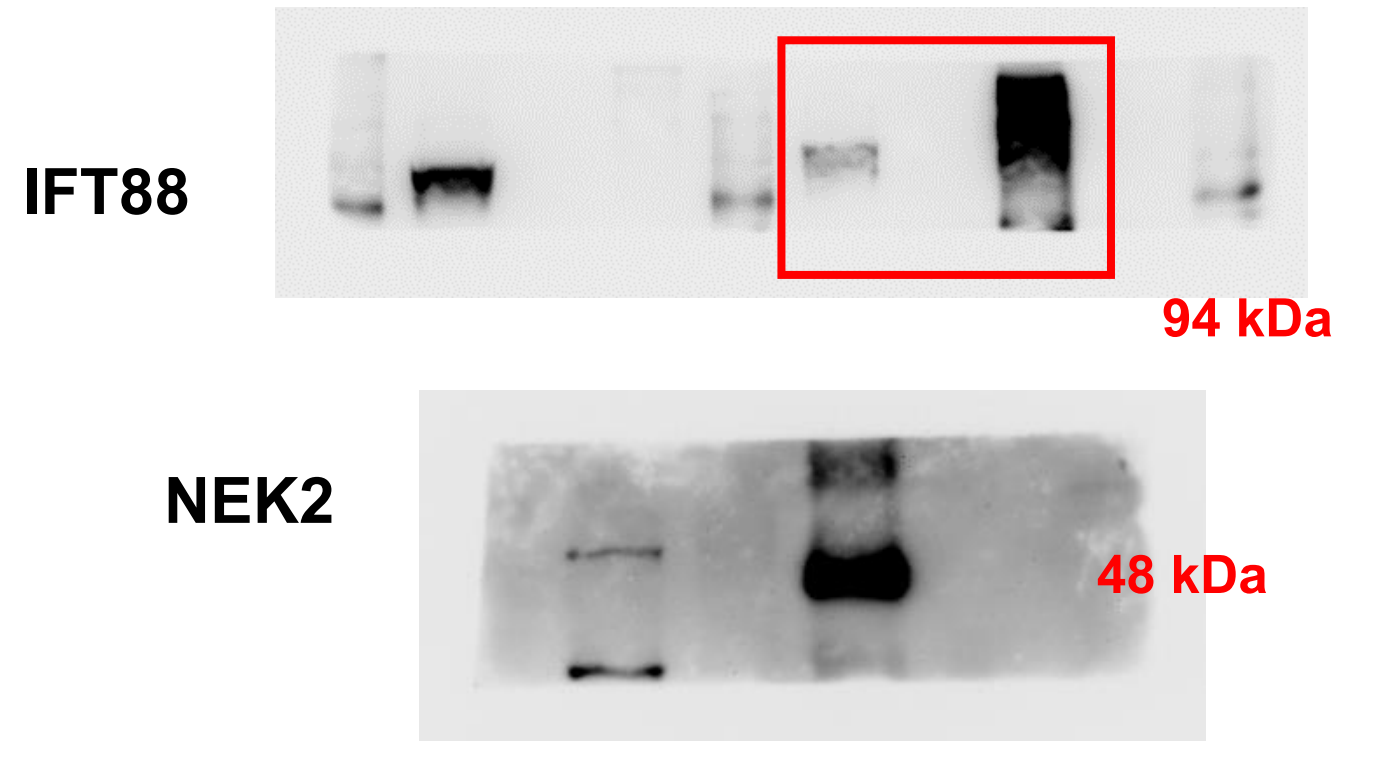

Figure S5C

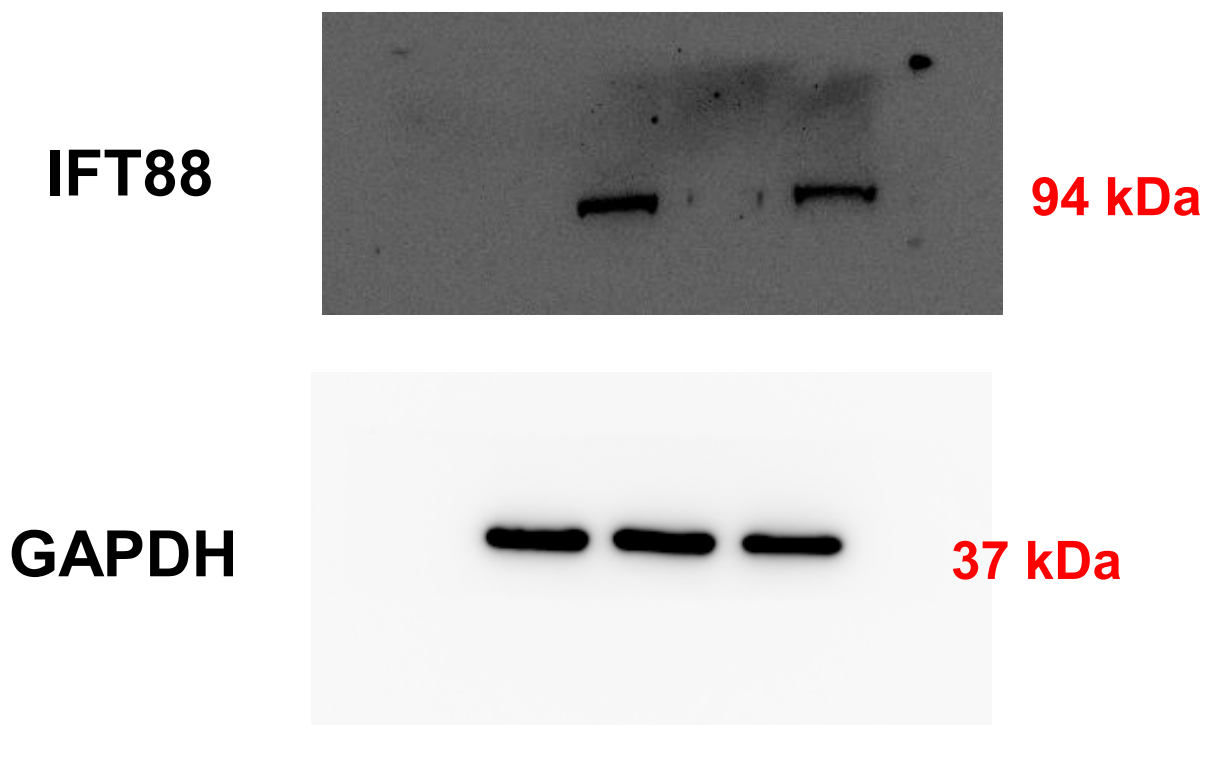

Figure S5D

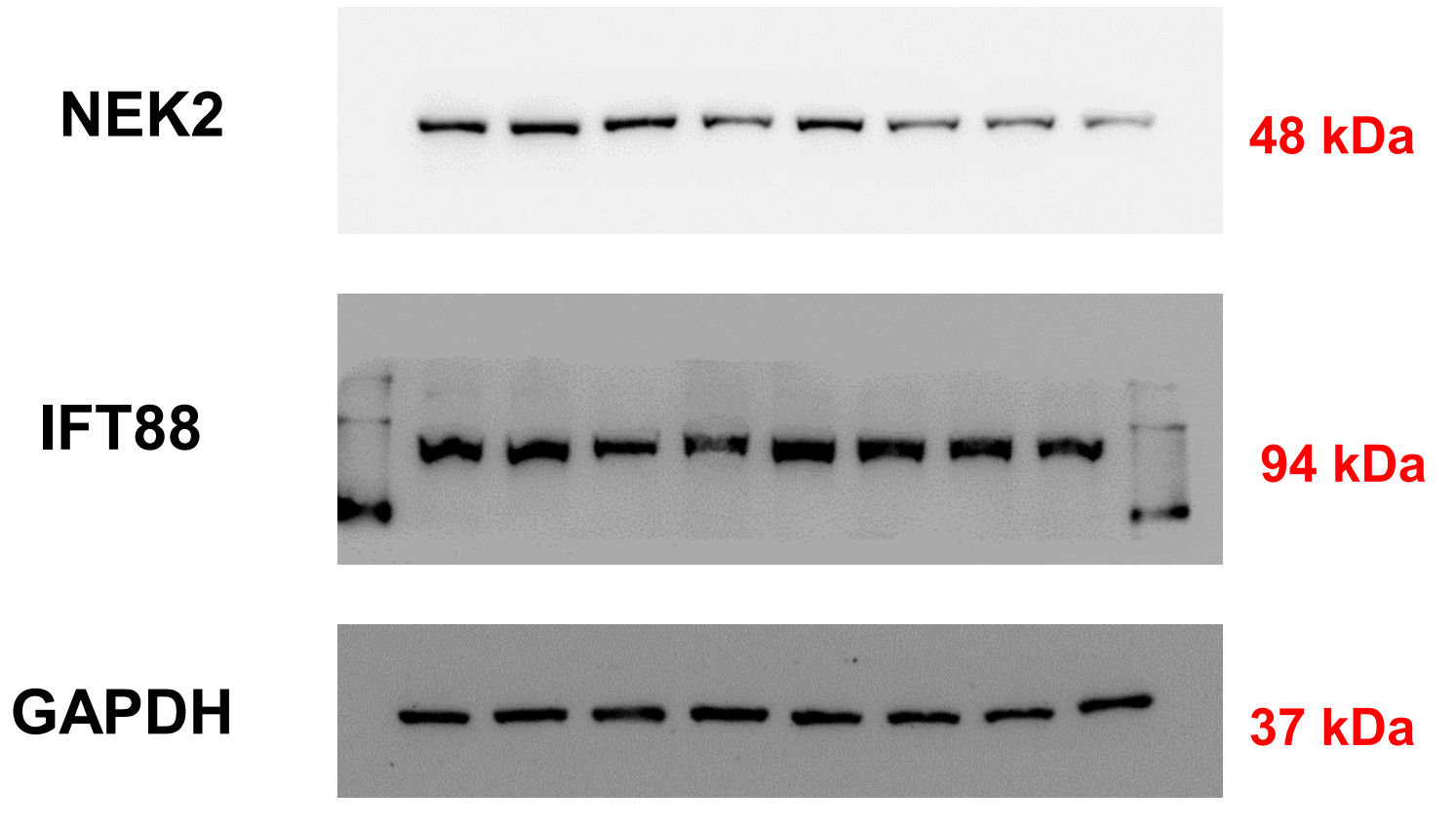

Figure S5F

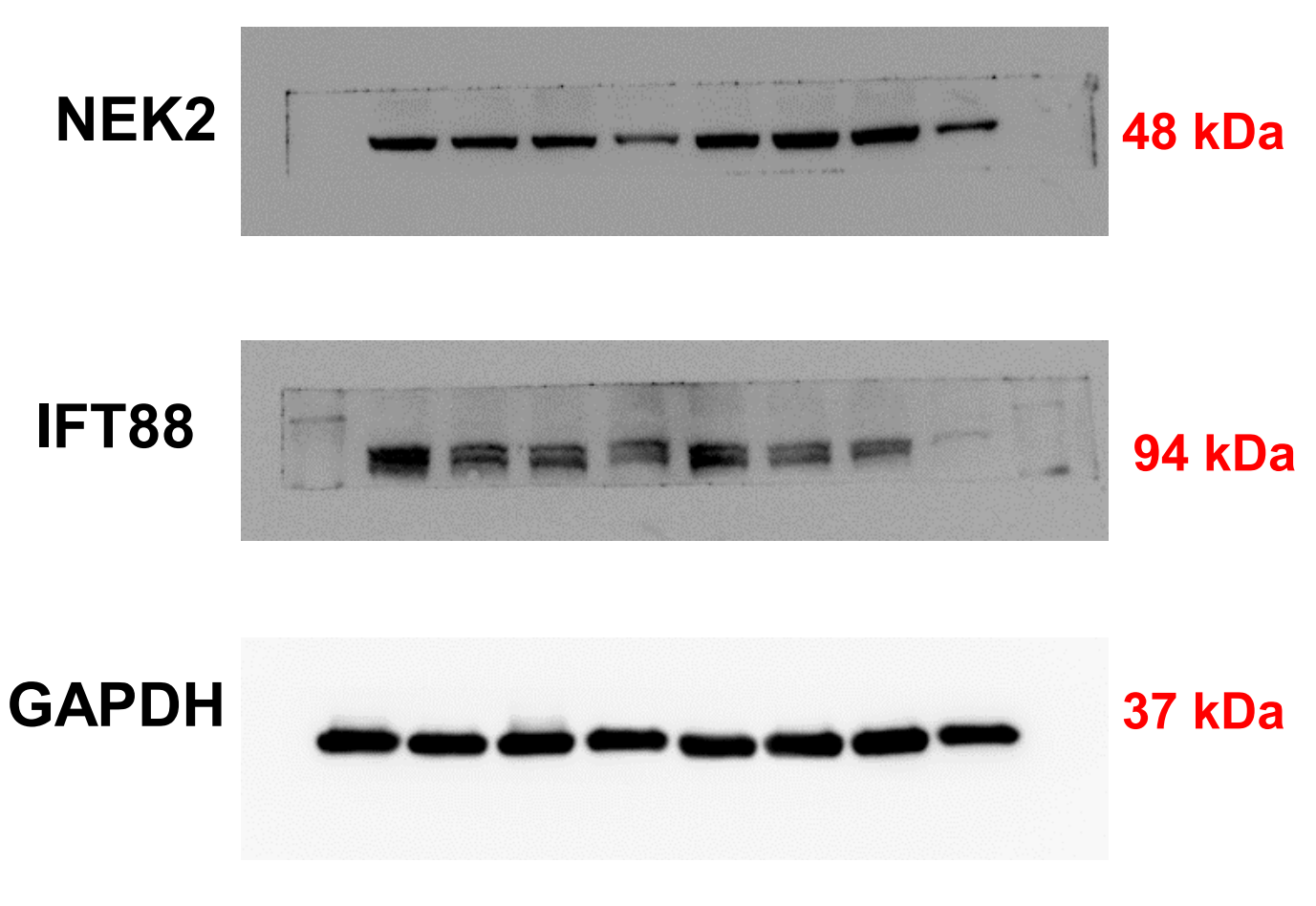

Figure S5H

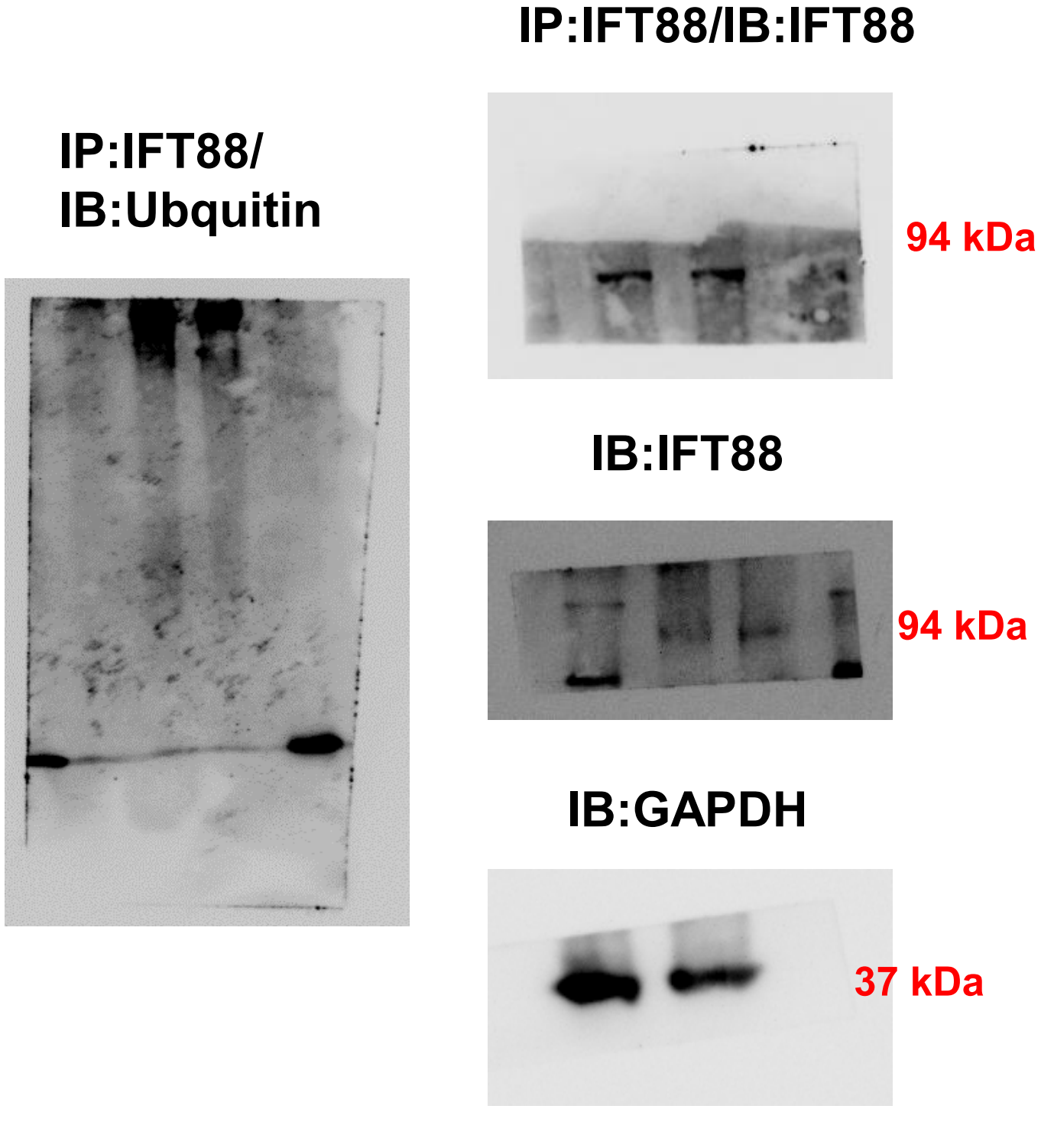

Figure S5I

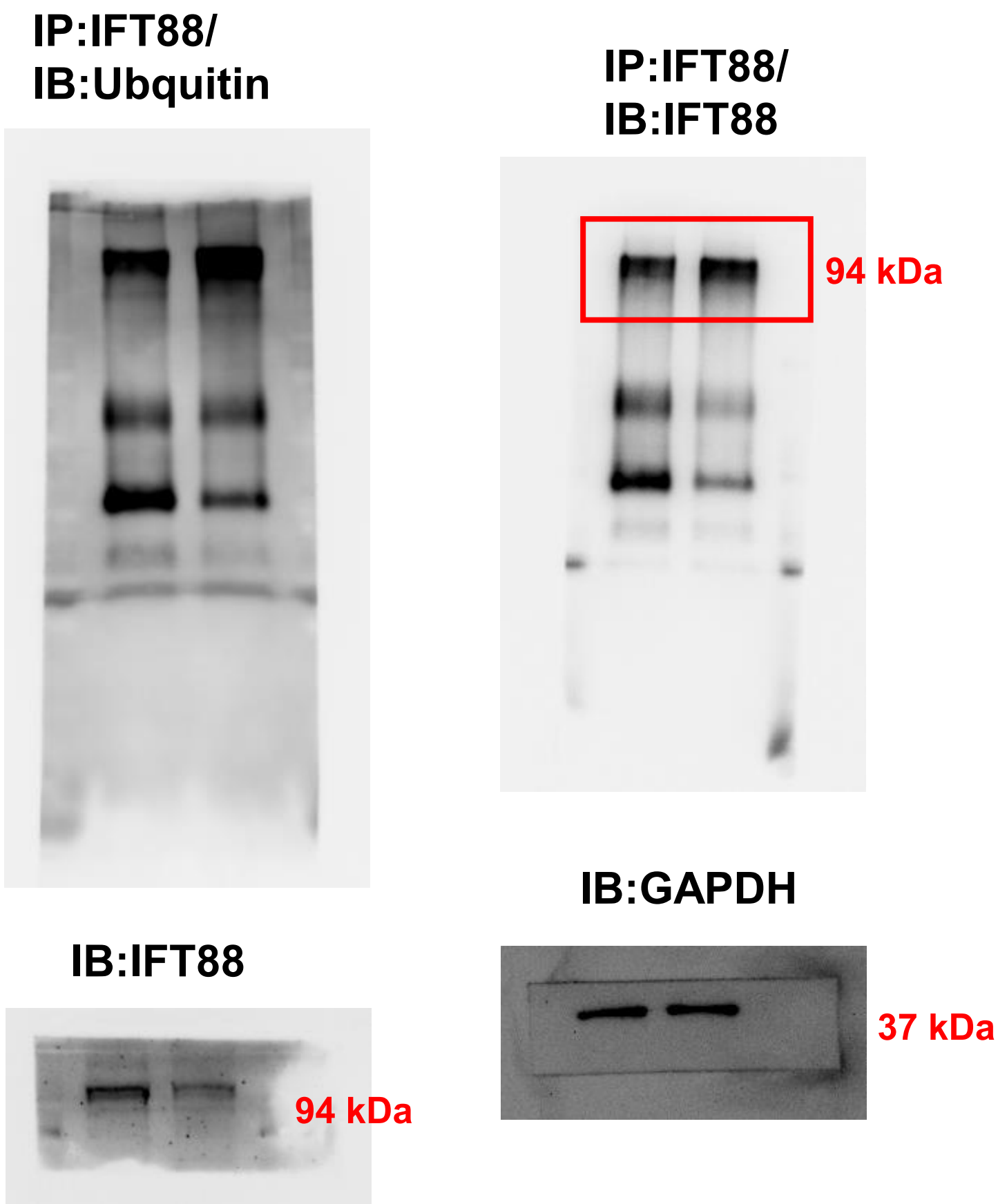

Figure S5J

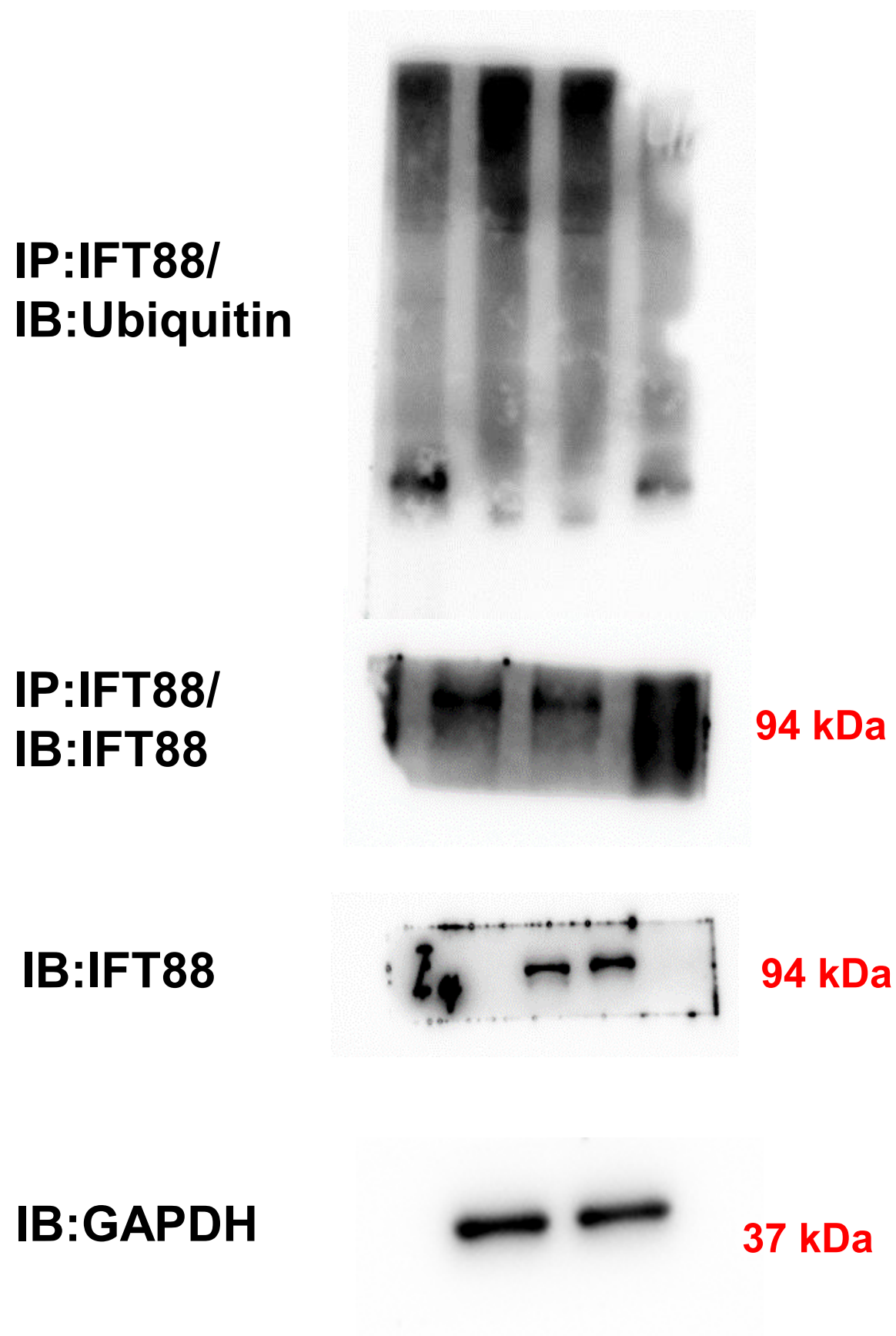

Figure S5K

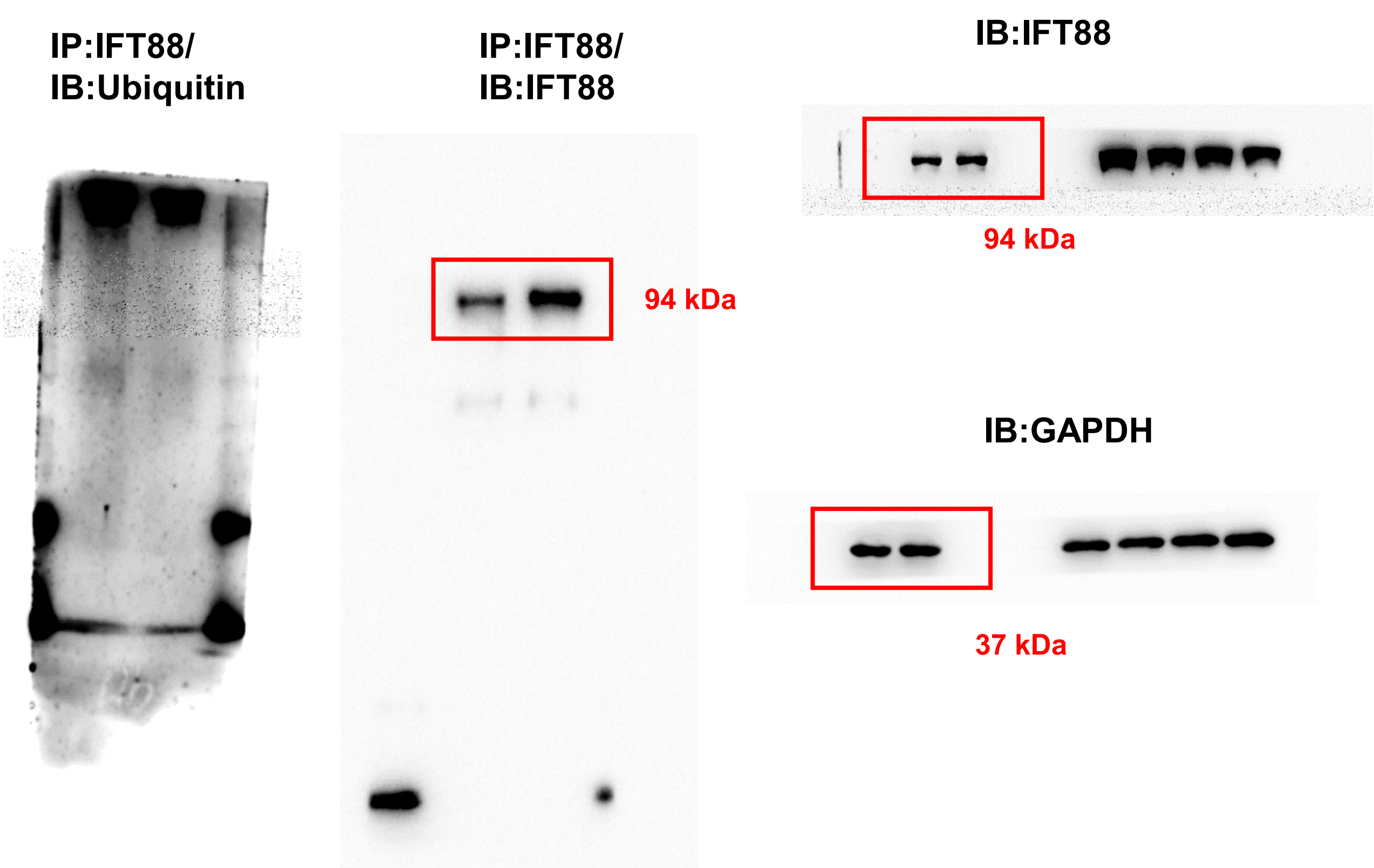

Figure S6A

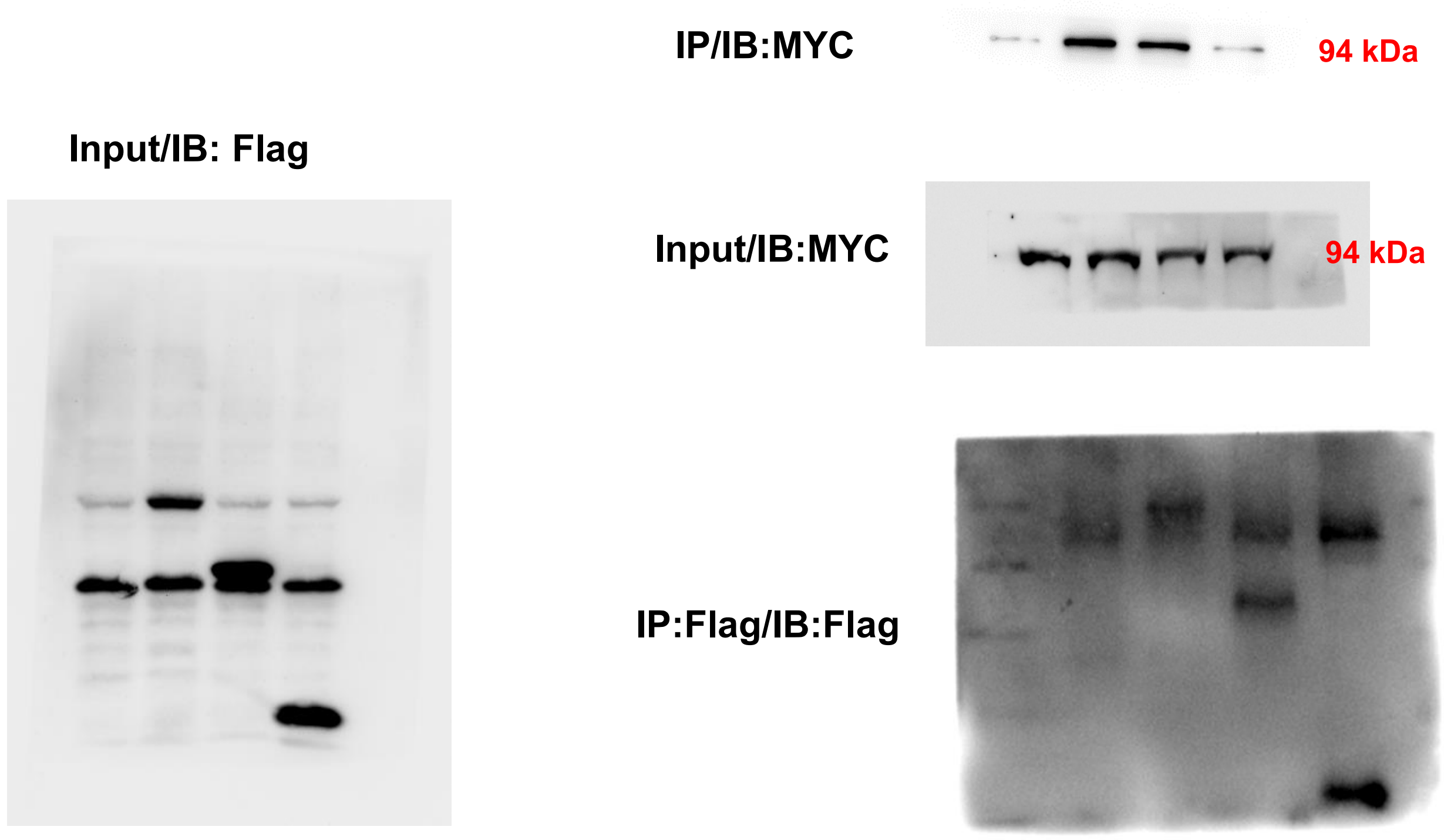

Figure S6B

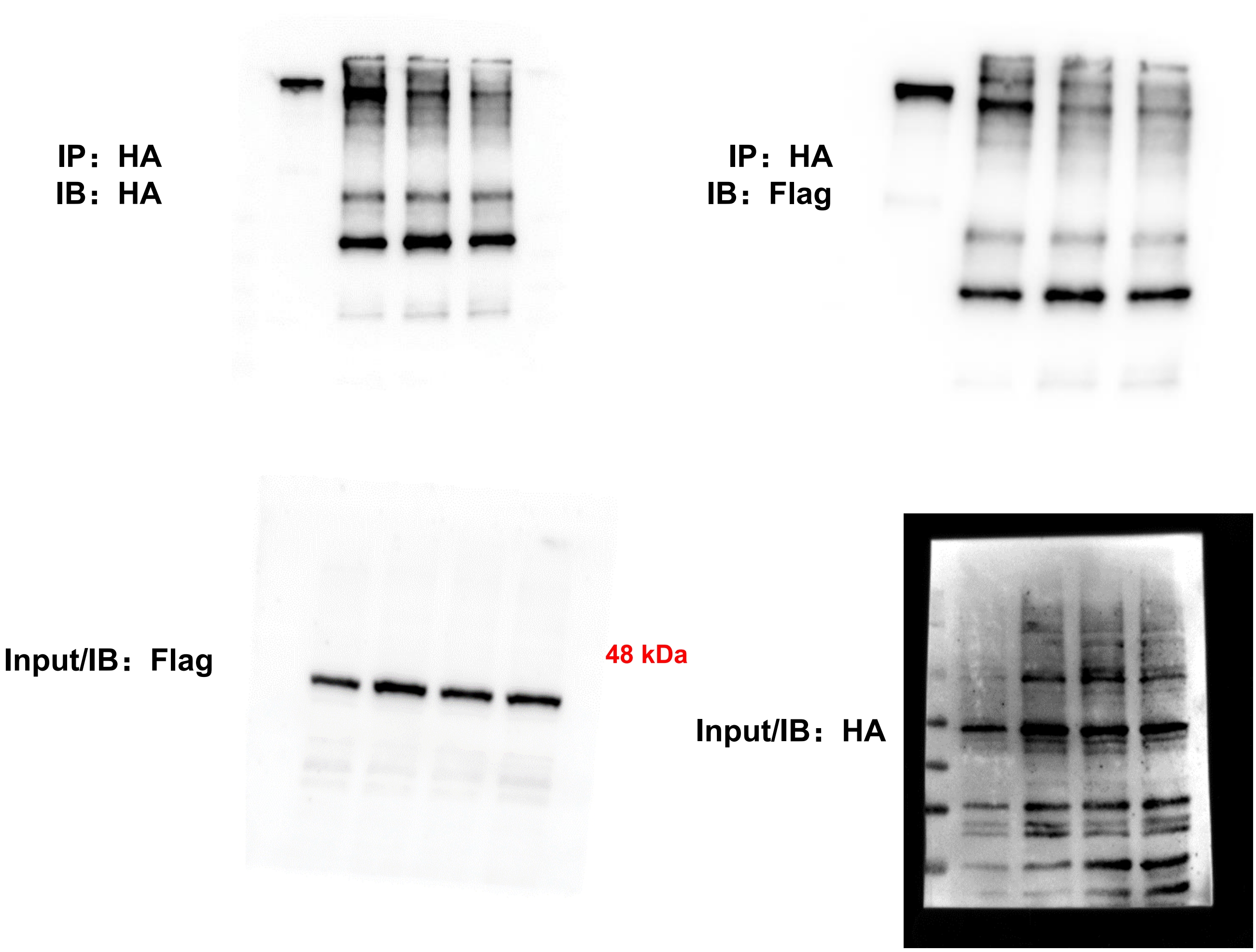

Figure S6C

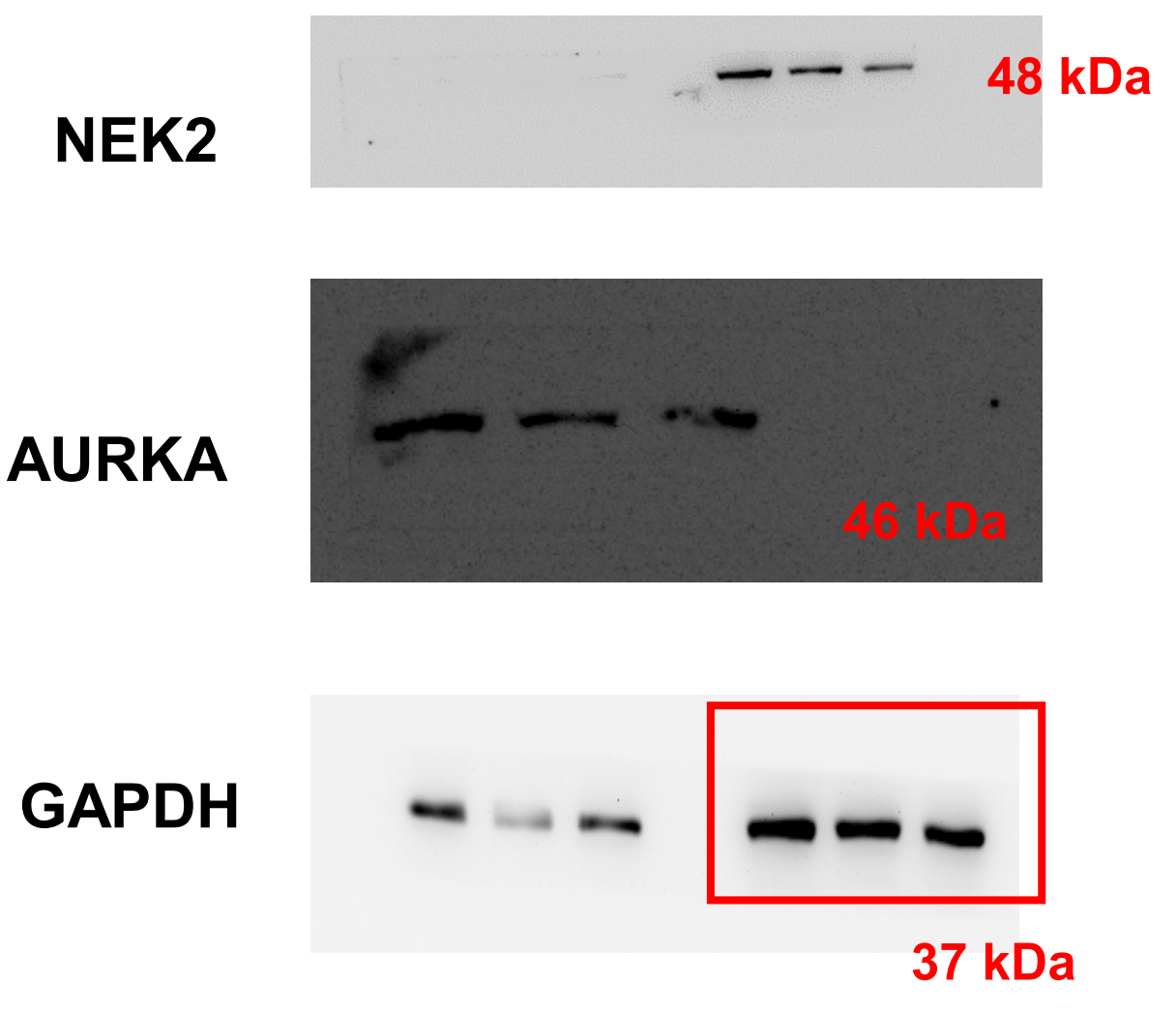

Figure S6D

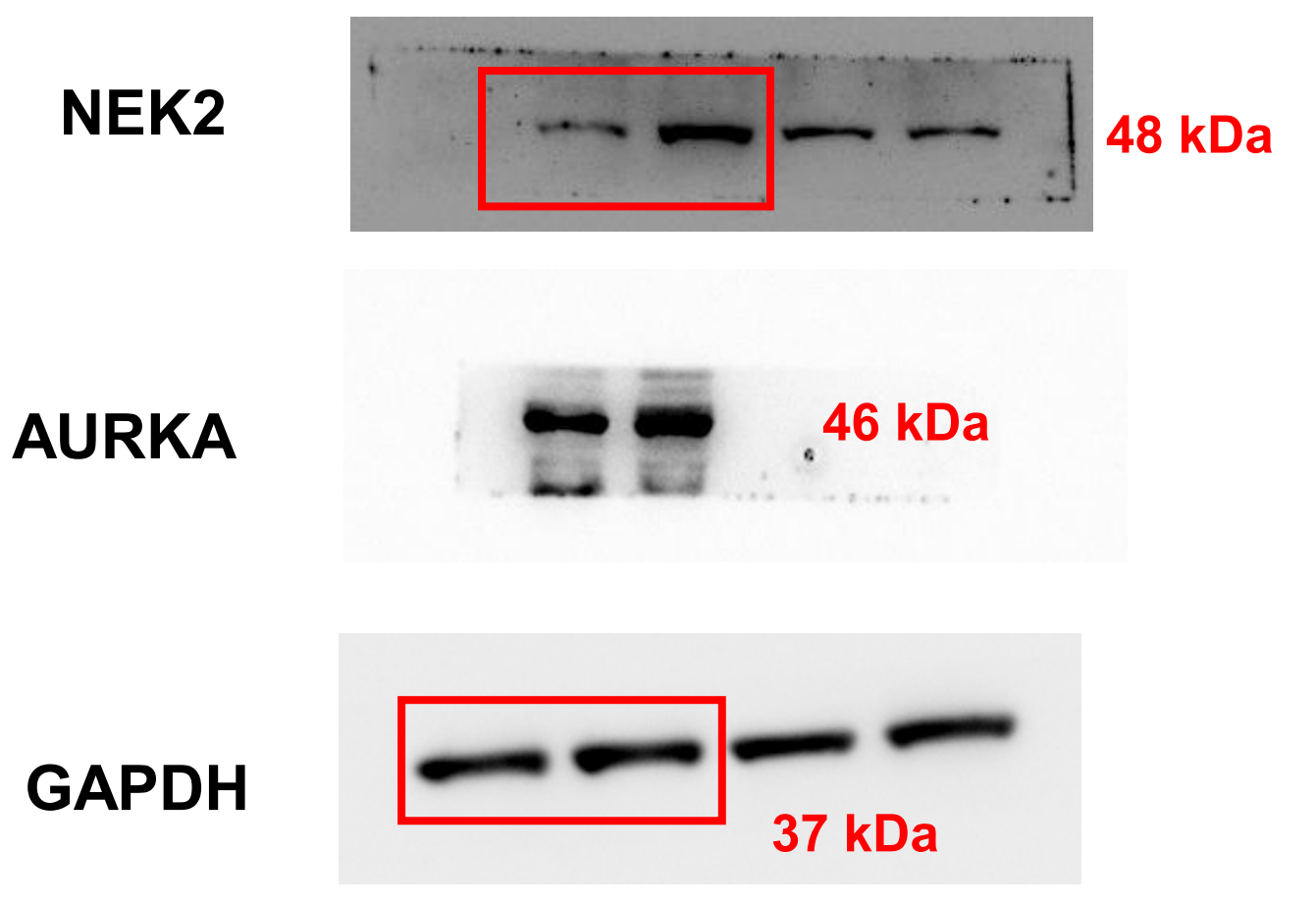

Figure S6E

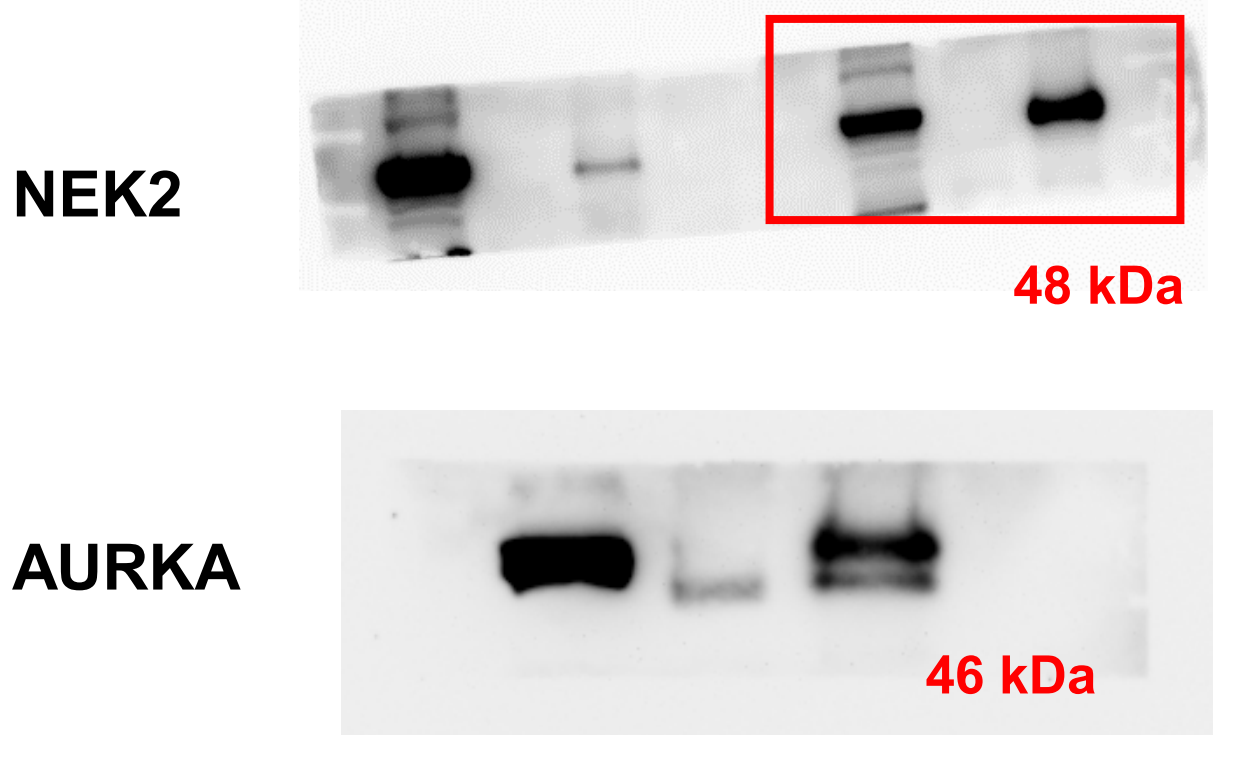

Figure S6F

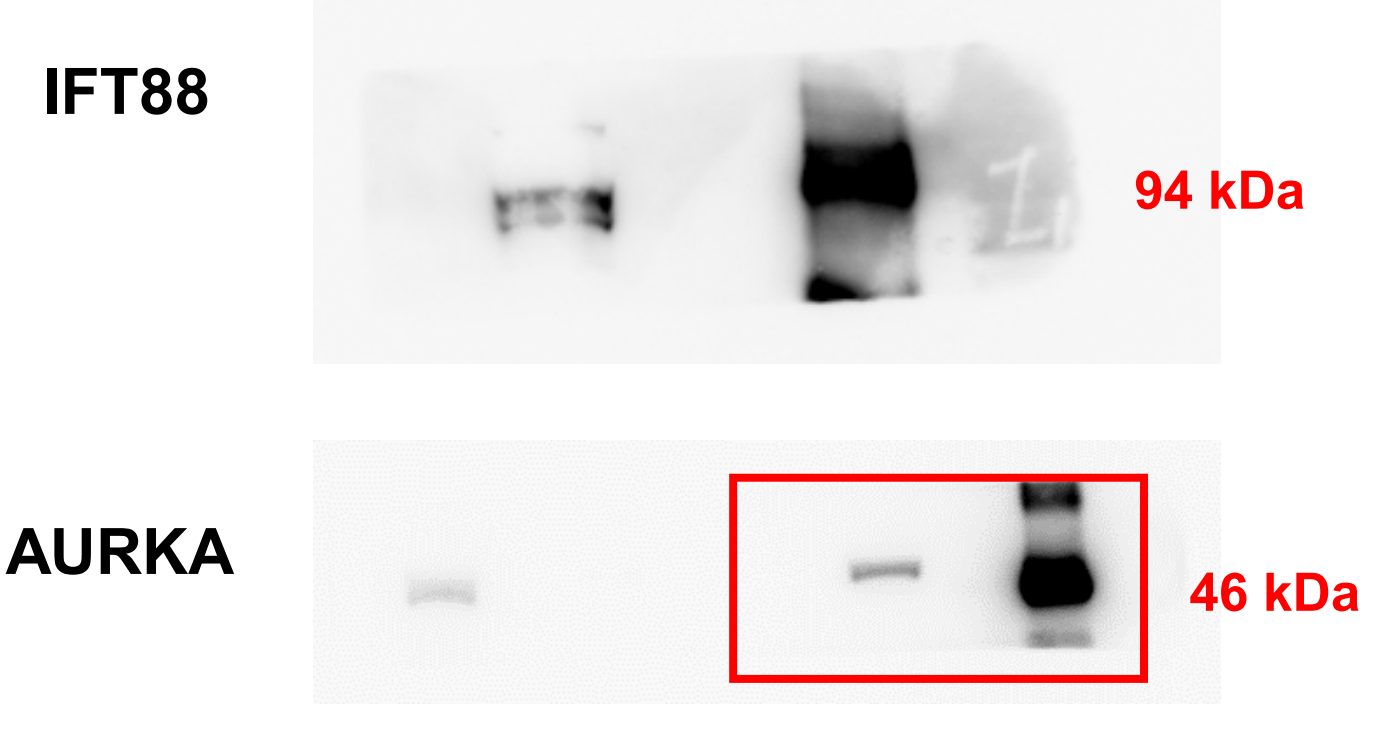

Figure S6G

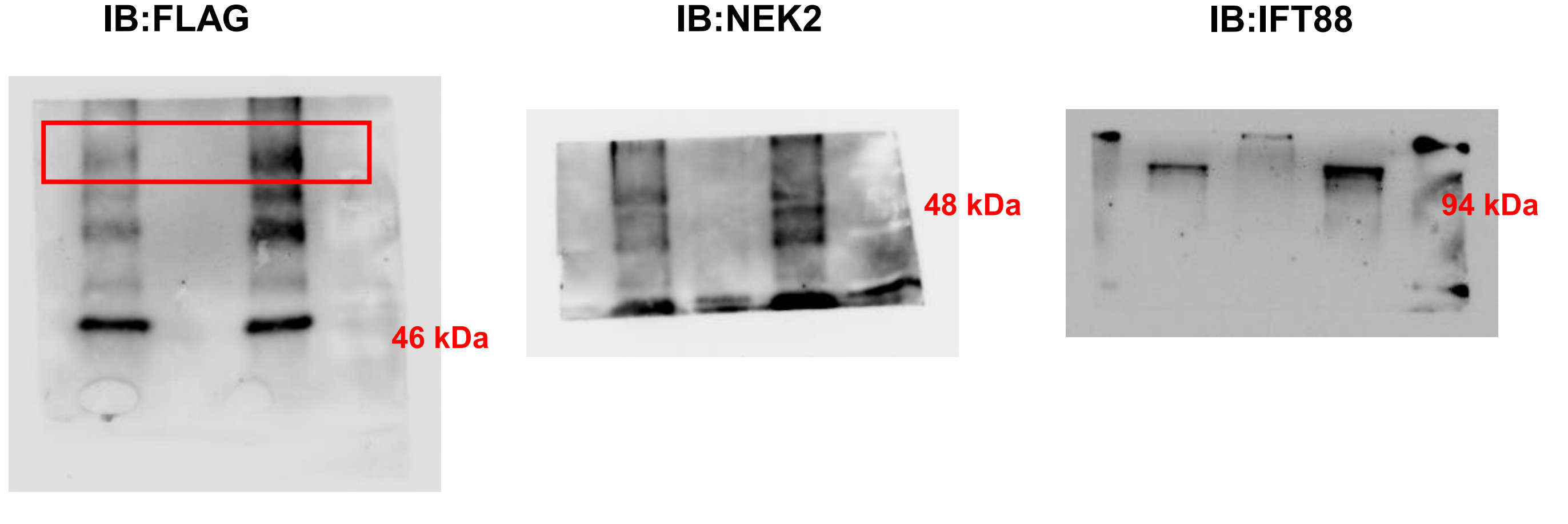

Figure S7K

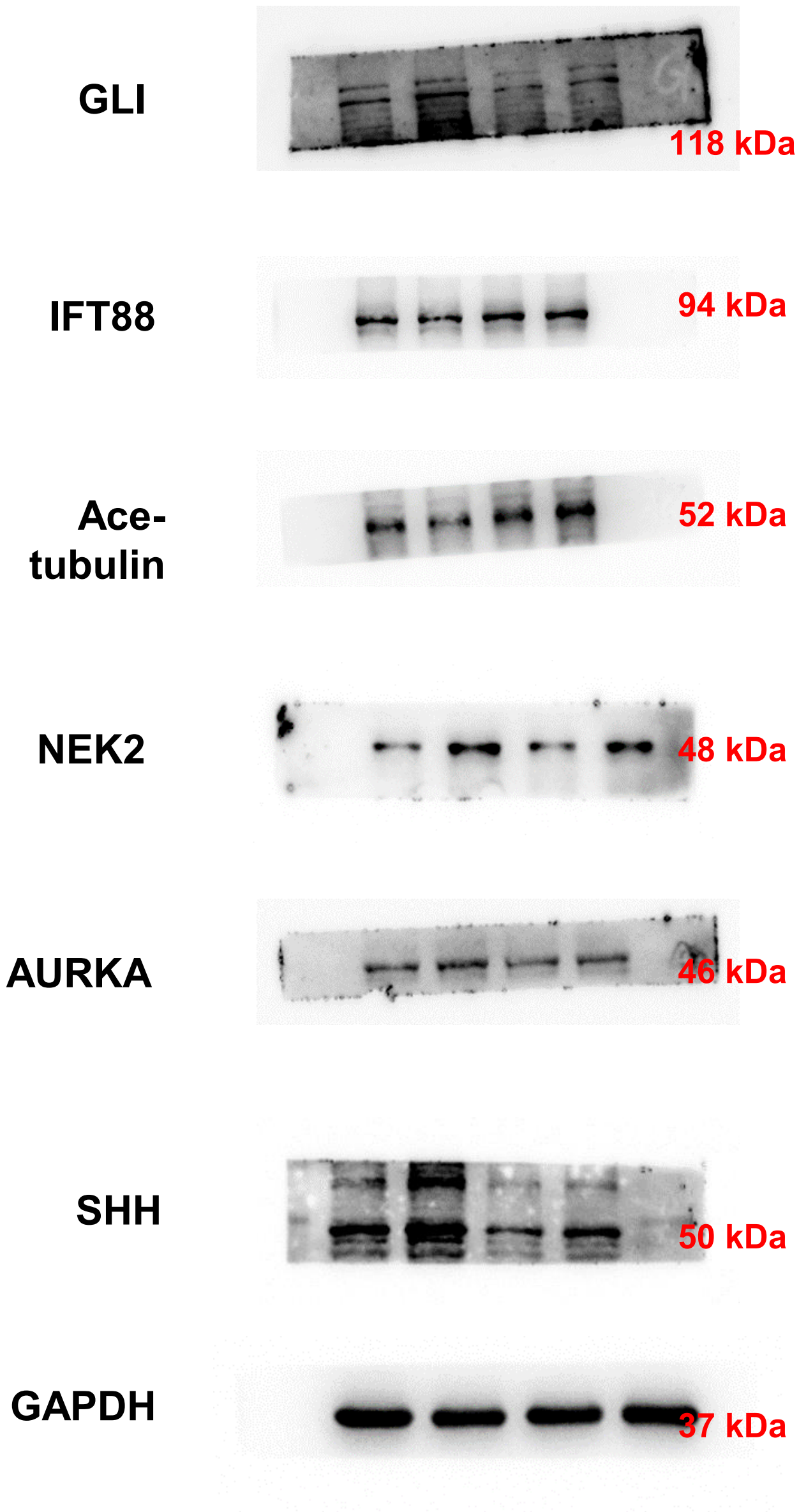

Figure S7L

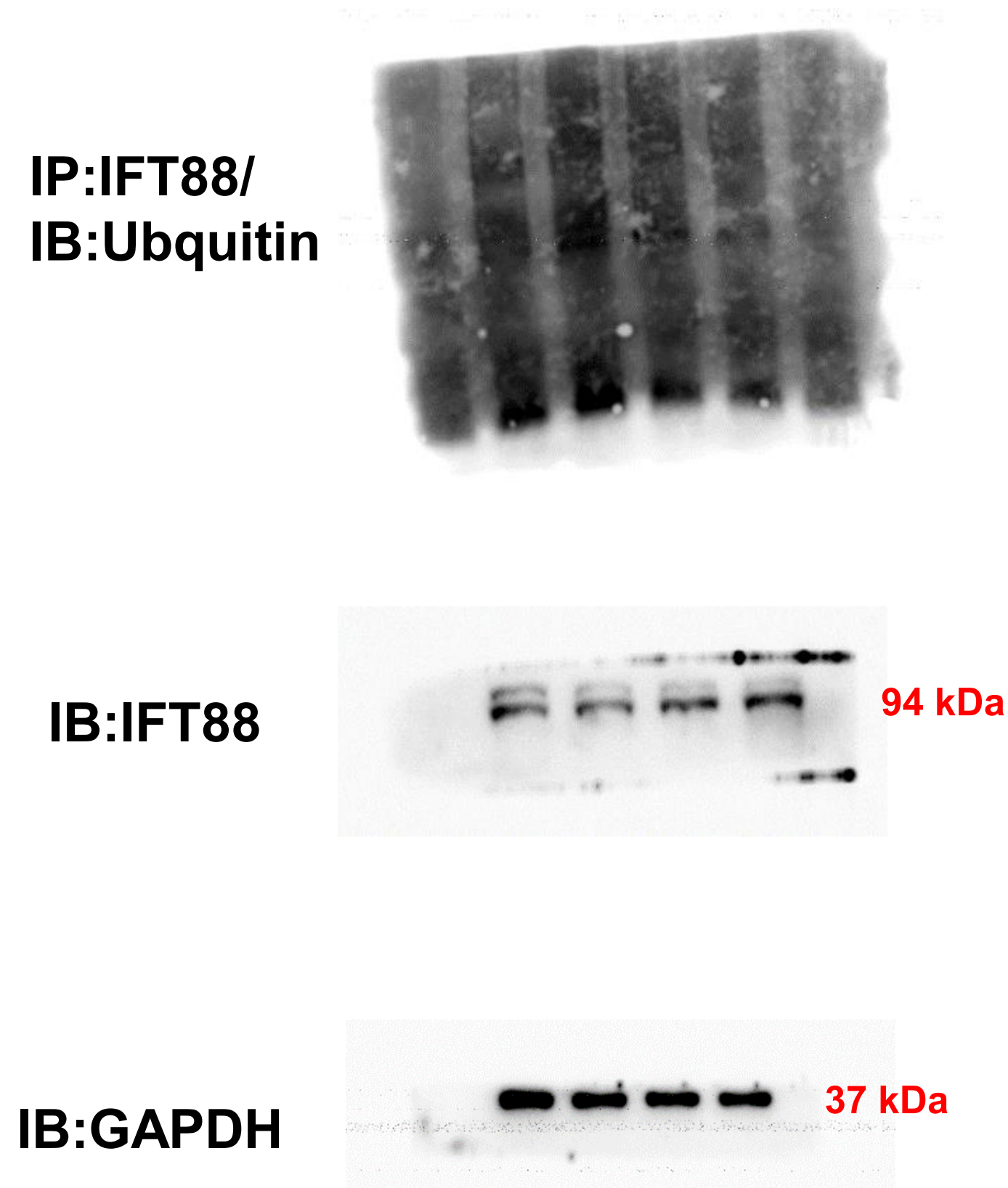

Supplement: Supplementary file 1 [file 41419_2026_9203_MOESM1_ESM.pdf]
