# Supplementary material for: NEK2-AURKA axis drives ubiquitin-dependent IFT88 degradation to suppress ciliogenesis and promote basal-type bladder cancer
Source: Cell Death Dis. 2026 Aug 22;17(1):903. doi: 10.1038/s41419-026-09203-0 (PMC13690740; doi:10.1038/s41419-026-09203-0)
Supplement: Supplementary file 2 [file 41419_2026_9203_MOESM2_ESM.docx]

**Fig. S1 | Loss of primary cilia promotes proliferation and migration in MIBC. (A, B)** mRNA expression levels of genes associated with primary cilia degradation and formation across six consensus MIBC subtypes; statistical significance between groups was assessed by t-test. **(C–F)** Immunofluorescence staining for acetylated tubulin and DAPI in IFT88-silenced and IFT88-overexpressing 5637 cells, with quantification of ciliated cell rates. For each experimental condition, two randomly selected non-overlapping 800× microscopic fields were analyzed from each of three independent glass coverslips, resulting in six analyzed regions per condition. The percentage of ciliated cells was calculated as the number of cells with a visible acetylated tubulin-positive cilium divided by the total number of DAPI-positive cells in the same field. **(G, H)** Western blot analysis of IFT88, acetylated tubulin, and ARL13B protein levels in 5637 control cells and cells with IFT88 knockdown or overexpression. **(I, L)** CCK8 assays assess cell viability of 5637 cells following IFT88 silencing or overexpression. **(J, M)** Colony formation assays evaluate growth of 5637 cells in control, IFT88 knockdown, and IFT88 overexpression groups. **(K, N)** Transwell assays measure migration capacity of 5637 cells in control, IFT88 knockdown, and IFT88 overexpression groups. Statistical significance: *, P < 0.05; **, P < 0.01; ***, P < 0.001; ****, P < 0.0001.

**Fig. S2 | NEK2 inhibits primary cilia formation and promotes tumor progression. (A)** Western blot analysis of NEK2 expression in T24 cell following serum starvation for 24 and 48 hours, and after serum restoration for 24 hours. **(B)** Western blot validates NEK2 knockdown efficiency in 5637 cells. **(C)** CCK8 assay measures cell proliferation in 5637-control and NEK2 knockdown groups. **(D)** Wound healing assay evaluates migration in 5637-siNC and 5637-siNEK2-#1 cells at 0, 24, and 48 hours. **(E)** Colony formation assay quantifies tumor growth in 5637-control and NEK2-silenced groups, with colony numbers compared by t-test. **(F)** Transwell assay assesses migration in 5637-siNC, siNEK2-#1, and siNEK2-#2 groups; relative migration was calculated and compared by t-test. **(G)** Immunofluorescence staining for acetylated tubulin quantifies the percentage of ciliated cells in 5637-siNC, siNEK2-#1, and siNEK2-#2 groups. For each experimental condition, two randomly selected non-overlapping 800× microscopic fields were analyzed from each of three independent glass coverslips, resulting in six analyzed regions per condition. The percentage of ciliated cells was calculated as the number of cells with a visible acetylated tubulin-positive cilium divided by the total number of DAPI-positive cells in the same field. **(H)** Western blot detection of NEK2, IFT88, and acetylated tubulin protein levels in 5637-siNC, siNEK2-#1, and siNEK2-#2 groups. **(I)** Western blot confirms NEK2 overexpression in 5637 cells. **(J)** CCK8 assay evaluates proliferation in 5637-vector and NEK2-overexpression groups over days 1–4. **(K)** Wound healing assay evaluates migration in 5637-vector and NEK2-overexpression groups at 0, 12, and 36 hours. **(L)** Colony formation assay quantifies tumor growth in 5637-vector and NEK2-overexpression groups, with statistical comparison by t-test. **(M)** Transwell assay assesses migration in 5637-vector and NEK2-overexpression groups; relative migration was calculated and compared by t-test. **(N)** Immunofluorescence staining for acetylated tubulin quantifies the percentage of ciliated cells in 5637-vector and NEK2-overexpression groups. For each experimental condition, two randomly selected non-overlapping 800× microscopic fields were analyzed from each of three independent glass coverslips, resulting in six analyzed regions per condition. The percentage of ciliated cells was calculated as described above. **(O)** Western blot detects NEK2, IFT88, and acetylated tubulin protein levels in 5637-vector and NEK2-overexpression groups.

**Fig. S3** **(A)** The percentage of surviving cells following exposure to increasing concentrations of INH1 in two luminal (SW780, RT4) and two basal (5637, T24) cell lines. Statistical significance: NS, not significant; *, P < 0.05; **, P < 0.01; ***, P < 0.001; ****, P < 0.0001. **(B)** Colony formation assays reveal the growth characteristics of luminal (SW780, HT-1376, RT4) and basal (J82, 5637, T24) bladder cancer cells following treatment with varying concentrations of INH1 (0, 5, 10, 20 μM).

**Fig. S4 | NEK2 inhibits primary cilia formation by downregulating IFT88 expression. (A, C)** Immunofluorescence assessed the percentage of ciliated cells in 5637-siNC, siNEK2, siIFT88, and siNEK2+siIFT88 groups **(A)**, and in 5637-vector, NEK2-overexpression, IFT88-overexpression, and NEK2+IFT88-overexpression groups **(C)**. For each experimental condition, two randomly selected non-overlapping 800× microscopic fields were analyzed from each of three independent glass coverslips, resulting in six analyzed regions per condition. The percentage of ciliated cells was calculated as the number of cells with a visible acetylated tubulin-positive cilium divided by the total number of DAPI-positive cells in the same field. **(B, D)** Western blot assays assessed NEK2, IFT88, and acetylated tubulin protein levels in 5637-siNC, siNEK2, siIFT88, siNEK2+siIFT88 groups **(B)**, and in 5637-Vector, NEK2-overexpression, IFT88-overexpression, NEK2+IFT88-overexpression groups**(D)**. **(E)** Transwell assay assessed migration ability in 5637-siNC, siNEK2, siIFT88, and siNEK2+siIFT88 cells. **(F)** Transwell assay evaluated migration in 5637-Vector, NEK2-overexpression, IFT88-overexpression, and NEK2+IFT88-overexpression cells. **(G)** CCK8 assay measured proliferative capacity in 5637-Vector, NEK2-overexpression, IFT88-overexpression, and NEK2+IFT88-overexpression groups. **(H)** Western blot analysis of acetylated tubulin, IFT88, and NEK2 expression following treatment with different concentrations of JH295 (0, 0.25, and 0.5 μM). **(I)** Western blot detection of IFT88, acetylated tubulin, and NEK2 in control and IFT88-overexpression cells with or without JH295 treatment. Statistical significance: *, P < 0.05; **, P < 0.01; ***, P < 0.001; ****, P < 0.0001.

**Fig. S5 | NEK2 interacts with IFT88 and regulates its ubiquitination levels. (A, B)** Co-immunoprecipitation (Co-IP) and western blot analyses demonstrate an interaction between NEK2 and IFT88 in 5637 cells. **(C)** Western blot analysis of IFT88 expression in NEK2-overexpressing cells with or without MG132 treatment, indicating the role of proteasomal degradation. **(D, E)** Western blot analyses of NEK2 and IFT88 expression in 5637-siNC and siNEK2 cells treated with cycloheximide (CHX) for various durations; IFT88 degradation rates were subsequently quantified. **(F, G)** Western blot analyses of NEK2 and IFT88 expression in 5637-vector and NEK2-overexpressing cells treated with CHX over time; IFT88 degradation rates were quantified. **(H, I)** Co-IP and western blot analyses assess the ubiquitination levels of IFT88 following NEK2 silencing or overexpression in T24 cells. **(J, K)** Co-IP and western blot analyses detect the ubiquitination levels of IFT88 after JH295 treatment in T24 and 5637 cells.

**Fig. S6 | AURKA acts downstream of NEK2 and interacts with both IFT88 and NEK2 at the protein level. (A)** Myc-IFT88 was co-transfected with Flag-NEK2 or Flag-NEK2-truncated mutants into 293T cells for 48 h, cell lysates were immunoprecipitated using anti-Flag antibody and immunoblotted with antibodies to Myc or Flag tags. **(B)** Flag-NEK2 was co-transfected with HA-IFT88 or HA-IFT88-truncated mutants into 293T cells for 48h, cell lysates were immunoprecipitated using anti-HA antibody and immunoblotted with antibodies to Flag or HA tags. **(C-D)** Western blot analysis reveals NEK2 and AURKA protein levels in 5637 cells following NEK2 knockdown and overexpression. **(E-G)** Co-immunoprecipitation (Co-IP) and western blot assays demonstrate that AURKA (Flag-tagged AURKA) interacts with NEK2 and IFT88 in 5637 cells.

**Fig. S7 | NEK2 regulates primary cilia formation via AURKA. (A, B)** 5637-control, NEK2-overexpression, siAURKA, and NEK2-overexpression+siAURKA cells were fixed and stained for acetylated tubulin; the percentage of ciliated cells was quantified. **(C, D)** 5637-control, siNEK2, AURKA-overexpression, and siNEK2+AURKA-overexpression cells were similarly stained and analyzed for ciliated cell percentage. **(E, F)** Transwell assays assessed migration ability in 5637-control, NEK2-overexpression, siAURKA, and NEK2-overexpression+siAURKA groups. **(G, H)** Colony formation assays evaluated cell growth in 5637-control, NEK2-OE, siAURKA, and NEK2-OE+siAURKA groups. **(I, J)** Colony formation assays evaluated cell growth in 5637-control, siNEK2, AURKA-overexpression, and siNEK2+AURKA-overexpression groups. **(K)** Western blot analysis of SHH pathway markers (GLI and SHH), acetylated tubulin, IFT88, NEK2, and AURKA in 5637-control, NEK2-overexpression, siAURKA, and NEK2-overexpression+siAURKA groups. **(L)** Co-IP and western blot assays determined ubiquitination levels and expression of IFT88 in 5637-control, NEK2-overexpression, and NEK2-overexpression+siAURKA groups. Statistical significance: *, P < 0.05; **, P < 0.01; ***, P < 0.001; ****, P < 0.0001. OE, Overexpression.
